# Integrated Transcriptome and Binding Sites Analysis Implicates E2F in the Regulation of Self-Renewal in Human Pluripotent Stem Cells

**DOI:** 10.1371/journal.pone.0027231

**Published:** 2011-11-04

**Authors:** Hock Chuan Yeo, Thian Thian Beh, Jovina Jia Ling Quek, Geoffrey Koh, Ken Kwok Keung Chan, Dong-Yup Lee

**Affiliations:** 1 Bioprocessing Technology Institute, Agency for Science, Technology and Research (A*STAR), Singapore, Singapore; 2 Department of Chemical and Biomolecular Engineering, National University of Singapore, Singapore, Singapore; Keio University, Japan

## Abstract

Rapid cellular growth and multiplication, limited replicative senescence, calibrated sensitivity to apoptosis, and a capacity to differentiate into almost any cell type are major properties that underline the self-renewal capabilities of human pluripotent stem cells (hPSCs). We developed an integrated bioinformatics pipeline to understand the gene regulation and functions involved in maintaining such self-renewal properties of hPSCs compared to matched fibroblasts. An initial genome-wide screening of transcription factor activity using *in silico* binding-site and gene expression microarray data newly identified E2F as one of major candidate factors, revealing their significant regulation of the transcriptome. This is underscored by an elevated level of its transcription factor activity and expression in all tested pluripotent stem cell lines. Subsequent analysis of functional gene groups demonstrated the importance of the TFs to self-renewal in the pluripotency-coupled context; E2F directly targets the global signaling (e.g. self-renewal associated WNT and FGF pathways) and metabolic network (e.g. energy generation pathways, molecular transports and fatty acid metabolism) to promote its canonical functions that are driving the self-renewal of hPSCs. In addition, we proposed a core self-renewal module of regulatory interplay between E2F and, WNT and FGF pathways in these cells. Thus, we conclude that E2F plays a significant role in influencing the self-renewal capabilities of hPSCs.

## Introduction

Human embryonic stem cells (hESCs) are pluripotent, with the capability to differentiate into almost any cell type, unlike differentiated cells or lineage-committed cells [1]. In addition, hESCs are able to proliferate indefinitely by circumventing regulatory processes such as apoptosis and replicative senescence while retaining their pluripotent state, i.e. self-renewing. To do so, hESCs require both intrinsic and extrinsic molecular signals.

The role of certain intrinsic factors as intracellular master determinants of hESC properties was demonstrated when human fibroblasts (hFs) were successfully reprogrammed into human induced pluripotent stem cells (hiPSCs) by ectopic expression of the transcription factors (TFs), OCT4 and SOX2, together with either KLF4 and c-MYC [2], or NANOG and LIN28 [3]. Among the TFs, OCT4, SOX2 and NANOG constitute a conserved core transcriptional regulatory network that is essential for specifying the undifferentiated state of both hESCs and mouse embryonic stem cells (mESCs) [4], [5]. In this work, hESCs and hiPSCs are collectively referred to as human pluripotent stem cells (hPSCs).

Along with the intrinsic factors, extracellular molecular cues are also required to maintain the undifferentiated state of hESCs. For example, transforming growth factor β (TGF-β)/Activin A signaling activates the TFs, SMAD2/3, which in turn induce the expression of OCT4 and NANOG [6], [7] as well as component genes of the self-renewal associated fibroblast growth factor (FGF) pathway (FGF2, FGFR1/2/3) [8]. As another example, Wnt signaling promotes self-renewal through the activation of T cell factors, e.g. Tcf3, which regulate gene expression of Sox2, Oct4 and Nanog, and co-occupy promoters with these pluripotent factors [9], [10] while extracellular bone morphogenetic proteins (BMP) signaling induces differentiation through the activation of the TFs SMAD1/5/8. Thus, the transduction of various extracellular signals activates relevant TFs, thereby regulating the expression of master determinants and associated ESC properties [11]–[13].

**Figure 1:**
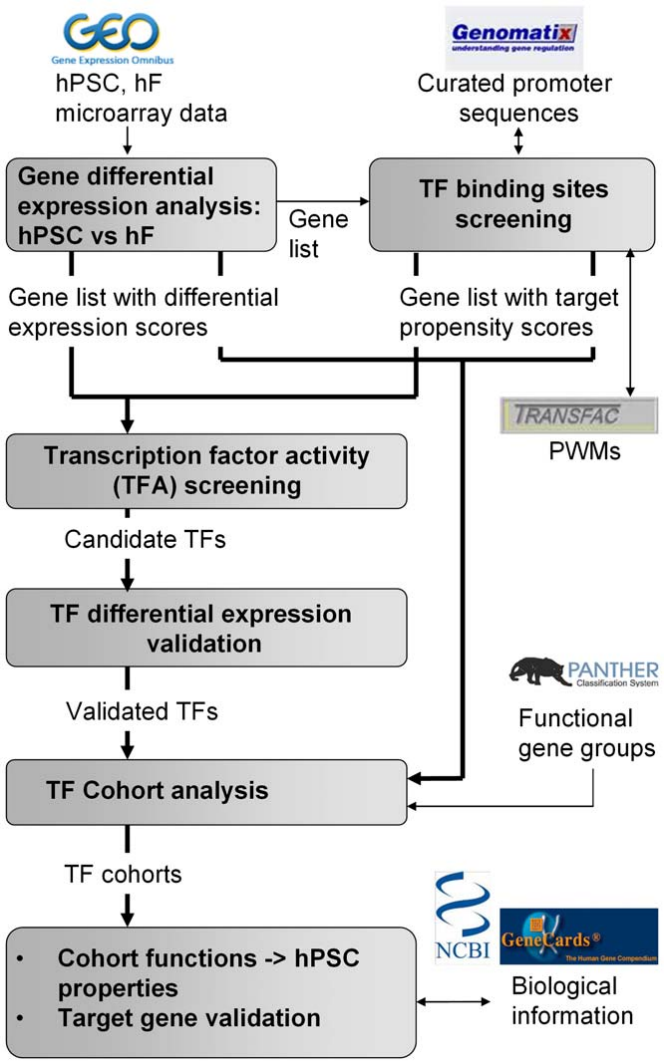
Integrated analysis pipeline. Differential expression analysis between hPSCs and hFs and the *in silico* screening of transcription factor (TF) binding sites in gene promoters, returned a gene list with corresponding scores. In screening for TFA, the enrichment of high target propensity genes in differentially-expressed genes was assessed. Significant TFs were further assessed for their transcript and protein levels. Subsequently, functional gene groups were evaluated for regulation by the validated TFs (target-cohort analysis). hPSC properties were then elucidated in terms of the functions of regulated gene groups as well as biological information from databases and literature. Trans-activation of interesting target-cohorts by the TF was also validated using luciferase assay.

Despite the key role of signaling pathways and other cellular participants in determining hESC properties, much remains to be done to understand their transcriptional regulation [13]. With the advent of high-throughput technologies generating genome-wide gene expression and TF binding data (such as ChIP-seq and ChIP-on-chip), in conjunction with the development of bioinformatics and computational methods to analyze them, it is now possible to shed more light on condition-specific transcriptional regulation and the corresponding functions at the systems-level. Thus, we developed an integrated bioinformatics pipeline to study the differential gene expressions between hPSCs and their differentiated counterparts, i.e. hFs, allowing us to better elucidate the transcriptionally regulated functions associated with self-renewal and/or reprogramming (Figure 1). At the outset, a list of genes with differential expression score was obtained by comparing microarray gene expression between the two cell types. The promoter sequences of the genes were then screened for enrichment of TF binding sites as reflected in the target propensity scores of the genes. Subsequently, the over-representation of high target propensity genes in sets of differentially-expressed genes is then utilized as a proxy to detect direct gene regulation by the candidate TF globally, which we termed transcription factor activity (TFA).

Remarkably, among a preliminary list of TFs with consistently significant TFA in various hPSC-hF comparisons, E2F was selected in this work based on its significantly smaller P-values compared to other candidates and consistent, elevated expressions in hPSCs which indicate its potential importance in genome-wide regulation. (See discussion for other candidates.) The E2F family of TFs is known as key regulator of cell proliferation and differentiation in eukaryotes, with target genes involved in apoptosis, DNA replication, cell cycle control, etc [14]–[16]. Although E2F regulation of these canonical functions may bring forth the notion that the TFs facilitate self-renewal, it remains strikingly undemonstrated in the pluripotency-coupled context. Furthermore, there are incentives to understand their global pleiotropic effects as they have the ability to target overlapping gene subsets with varying degree of antagonism, cooperativity and redundancy. Consequently, the E2F family has the highly precise roles of driving versus braking the cell cycle, promoting versus inhibiting programmed cell death, as well as maintaining stemness versus inducing differentiation, depending on their relative expressions and the stage of differentiation [14]–[17]. For example, both activator E2F1 (see introduction result) and suppressor E2F8 (data not shown) are highly expressed in hPSCs and it is unclear what are their net effects on cellular functions. In addition, the recently reported roles of new members (E2F7-8) mainly in early development [18], [19] suggests more unexplored regulatory mechanisms in the embryonic stage.

Thus, it would be helpful to qualify the effects of the TFs on individual gene groups and in the process, also uncovers new biological functions and mechanisms with the help of bioinformatics in a systematic manner. This would give us a better appreciation of the intricacies involved in the precise E2F control of self-renewal propensity in hPSCs, noting that the pluripotent state may present unique requirements during the development of an organism. Toward this end, we newly designed a methodology for identifying TF-regulated functional gene groups based on both regulatory and gene expression information. In the initial step, a novel test, termed target-cohort analysis, detects the presence of target-cohorts, or genes that have specific properties of potential target genes, compared to others in the group. Next, their reliability and relevance can be augmented with biological information from the literature and databases, and then used to help us explain hPSC properties. The trans-activation of selected target-cohorts by E2F was evaluated using a luciferase reporter assay with E2F over-expression. Taken together, it suggests that E2F transcriptionally coordinate signaling pathways (e.g. WNT, FGF), metabolism (e.g. energy generation pathways and molecular transports) and its canonical functions to promote self-renewal of hPSCs.

## Materials and Methods

### Gene expression data

Microarray data with accession number GSE9440 and GSE9832 were obtained from the Gene Expression Omnibus (GEO) database [20] to make the following hPSC-hF comparisons: (1) T3 hESCs vs fibroblast-like cells (T3 differentiated), (2) H1-OGN hESCs vs H1-OGN fibroblasts, (3) H1-OGN fibroblast iPS vs H1-OGN fibroblasts, (4) H1-OGN cloned fibroblasts iPS(cf16) vs H1-OGN cloned fibroblasts, (5) H1-OGN cloned fibroblasts iPS(cf32) vs H1-OGN cloned fibroblasts, (6) fetal lung fibroblast iPS vs fetal lung fibroblasts, and (7) neonatal fibroblast iPS vs neonatal fibroblast. Note that transcript probe sets with IDs ending with ‘_a_at’, ‘_s_at’ and ‘_x_at’ were omitted while the same gene at different genomic loci is treated as separate genes.

### Differential expression measures

For each hPSC-hF comparison, a significance analysis of microarray (SAM) [21] score was computed for the differential expression of individual genes and scaled to mean = 0 and standard deviation = 1. To pool information from different comparisons, the differential expression (score) for each gene is defined as the mean of the absolute value of the scaled SAM scores for different comparisons.

The dot product and covariance metrics were used as measures of similarity for differential expression scores, un-scaled and scaled by its average value respectively. To illustrate their usage, ***a*** and ***b*** are defined as arrays of scaled SAM scores of gene *A* and gene *B* respectively, under *n* different conditions and denoted as ***a*** = [*a_1_, a_2_, …, a_n_*] and ***b*** = [*b_1_, b_2_, …, b_n_*]. Then, *dp_B_* and *cov_B_* are the respective dot Product and covariance measures of similarity between the differential expression score of cellular regulator *A* (E2F) and gene *B*:
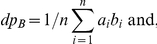


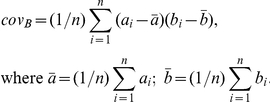
The corresponding similarity measures of differential expression between a cellular regulator and a group of *m* genes are defined as:
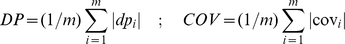



### Target propensity

TRANSFAC MATCH algorithm [22] was employed to identify putative binding sites in curated gene promoter sequences (∼−500 bp and +100 bp transcription start site) downloaded from Genomatix URL (http://www.genomatix.de/), using all 594 position weight matrices (PWMs) and a minimum false negative profile.

Next, let I and l_i_ be the total number of global promoters and the sequence length of promoter i respectively and, 
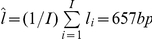
. It follows that the target propensity of a gene = 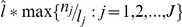, where J is the total number of alternative promoters for the gene, n_j_ is the number of binding sites in promoter j and l_j_ is the length of promoter j. Other than TFA evaluation, all E2F analyses were conducted based on the model ‘V$E2F1_Q6_01’ (NTTT[C/G][C/G]CG[C/G][C/G]) which reported the highest average −log P-value ( = 58.8) for TFA.

### Transcription factor activity (TFA) evaluation

Transcription factor activity refers to the state of direct gene regulation by the associated TF at a system-level. This can be suggested by the over-representation of high target propensity genes in sets of differentially-expressed genes, reported in the form of chi-square statistic. The maximum chi-square value among all threshold combinations of SAM scores and target propensity is used to infer the statistical significance of over-presentation. A linear relationship based on randomized rankings, exists between −log (P-value) and the maximum chi-square, allowing for extrapolation of significance on the basis of a relatively small number of simulations. More details can be found in Figure S1.

### Target-cohort analysis

The analysis screens for gene groups regulated by a cellular regulator, e.g. E2F. It evaluates if differential expression score and score similarities with the regulator increases significantly across iterative target propensity thresholds in each group. If so, it can be concluded that regulator activity can partly explain the greater scores of the higher target propensity subset (enriched in true targets), ceteris paribus. The interim P-value at each target propensity threshold is defined as the proportion of sampled groups that have score increment larger than the tested gene group. The final P-value is the proportion of sampled groups that have their minimum interim P-value among all thresholds, smaller than the tested gene group. Gene groups composed of 140 pathways, 244 molecular functions and 229 biological processes from the PANTHER database [23] were screened for this analysis. Details on pseudo-algorithm can be found in Supporting Information S1.

### Plasmids

Human promoter-firefly luciferase (FLuc) reporter constructs (Switchgear, Menlo Park, USA) were obtained for FRZB, SMAD1 and WNT5A. Mutations were made on the E2F motifs in the promoter regions of these constructs (Table S1) using the QuikChange Mutagenesis kit (Stratagene, La Jolla, CA, USA), and subsequently sequence-verified.

### Cell culture

hESC line (HES-3) (ES Cell International, Singapore), hiPSC lines hiPS (IMR-90) and hiPS (foreskin) [24] were cultured in medium conditioned by mitomycin-C-inactivated immortalized mouse embryonic fibroblast (DE-MEF) feeder supplemented with 4 ng/ml of FGF-2 (Invitrogen, Carlsbad, CA, USA) on Matrigel (BD Bioscience, San Diego, USA)-coated plates as previously described [25]. hF cell lines IMR-90, Hs27 and Hs68 (all from ATCC) were cultured at 37°C/5% CO2 in complete medium containing Dulbecco's modified Eagle's medium (DMEM; high glucose) and 10% fetal bovine serum (FBS) according to the manufacturer's protocol.

### RNA extraction and real-time PCR analysis

RNA samples were extracted from HES-3, hiPS (IMR-90), hiPS (foreskin), IMR-90, Hs27 and Hs68 using QIAGEN RNAeasy kit according to the manufacturer's instructions and reverse transcribed into cDNA using Superscript III reverse transcriptase (Invitrogen). The cDNA were used for quantitative real-time PCR analyses with gene-specific primer pairs. All samples were run in triplicates at a reaction volume of 25 µl containing Power SYBR Green PCR Master Mix (Applied Biosystems, Foster City, CA, USA), and 200 nM primers. The reaction was run on the ABI PRISM 7500 Sequence Detection System (Applied Biosystems) using the following amplification parameters: 2 min at 50°C, 10 min at 95°C, and 40 cycles of 15 sec at 95°C and 1 min at 60°C. Data were analyzed using the ΔΔCT method to obtain expression levels relative to endogenous GAPDH control in each sample as previously described [25]. Primers used for these analyses are provided in Table S2.

### Western Blot analysis

Cell lysate prepared using 1% Igepal lysis buffer were resolved on 4–12% NuPAGE gels (Invitrogen) and transferred onto polyvinylidene fluoride (PVDF) membranes (BioRad). Membranes were blocked in PBS with 5% low-fat milk and probed overnight with primary antibody mouse anti-E2F-1 (Millipore, Billerica, Massachusetts, USA). Loading consistency was determined with mouse anti-actin (1∶3000; Santa Cruz Biotechnology, Santa Cruz, CA, USA). The membrane was then followed by incubation with infrared fluorescent (IRDye)-labeled secondary antibodies (LI-COR Biosciences, Lincoln, Nebraska, USA) and signals were visualized using Odyssey Infrared Imaging System (LI-COR Biosciences).

### Luciferase reporter assay

0.6 µg of either wild-type (WT) or mutant promoter FLuc-reporter construct (See ‘Plasmid’ section, Materials and Methods) was co-transfected along with the E2F1/2/3 over-expression vectors (Open Biosystems, Huntsville, AL, USA) and pRL-TK internal control vector (0.012 µg) (Promega, Fitchburg, Wisconsin, USA) into hESC lines (HES-3) using Lipofectamine 2000 reagent (Invitrogen) according to manufacturer's instruction. 24 hours after transfection, medium was changed to either DE-MEF conditioned medium or embryoid body differentiation medium (KO-DMEM supplemented with 20% FBS, 1% nonessential amino acids, 1 mM L-glutamine, and 1% penicillin-streptomycin [all from Invitrogen] and 0.1 mM β-mercaptoethanol [Sigma, St. Louis, USA]) and cultured for two days with daily medium changing. Cells were harvested 72 h after transfection and assayed for luciferase activity using the Dual-Luciferase assay system (Promega) according to manufacturer's protocol and as previously described [26]. Briefly, cells were washed three times with PBS buffer and lysed with 1×Passive Lysis Buffer (PLB). Cell lysate (50 µl) was mixed with 100 µl of Luciferase Assay Reagent II and subsequently with 100 µl of Stop & Glo Reagent in a microplate. Luciferase activities were measured for luminescence by Infinite M200 microplate reader (Tecan, Switzerland). The luciferase activity of each construct was calculated relative to that of the vector control (pcDNA3.1) without over-expression. All transfection experiments were performed at least thrice using different batches of cells with different preparations of plasmid DNAs and similar results were obtained. Data were illustrated as mean ± standard deviation (SD) of a minimum of three experiments, each performed in triplicates.

## Results

### E2F is identified as global transcriptome regulator in hPSCs

We made global gene expression comparisons between hPSCs and their corresponding hFs or differentiated fibroblast-like cells (two hESC vs differentiated hESC, one hiPSC vs differentiated hESC, two hiPSC vs cloned differentiated hESC and two hiPSC vs hF) using two public microarray datasets (See Materials and Methods). The cell line variations in gene expression provided a stringent criterion to evaluate the relevance of TF candidates to gene regulation in hPSC, by requiring screened TF to show significant TFA in at least 6 hPSC-hF comparisons.

**Figure 2:**
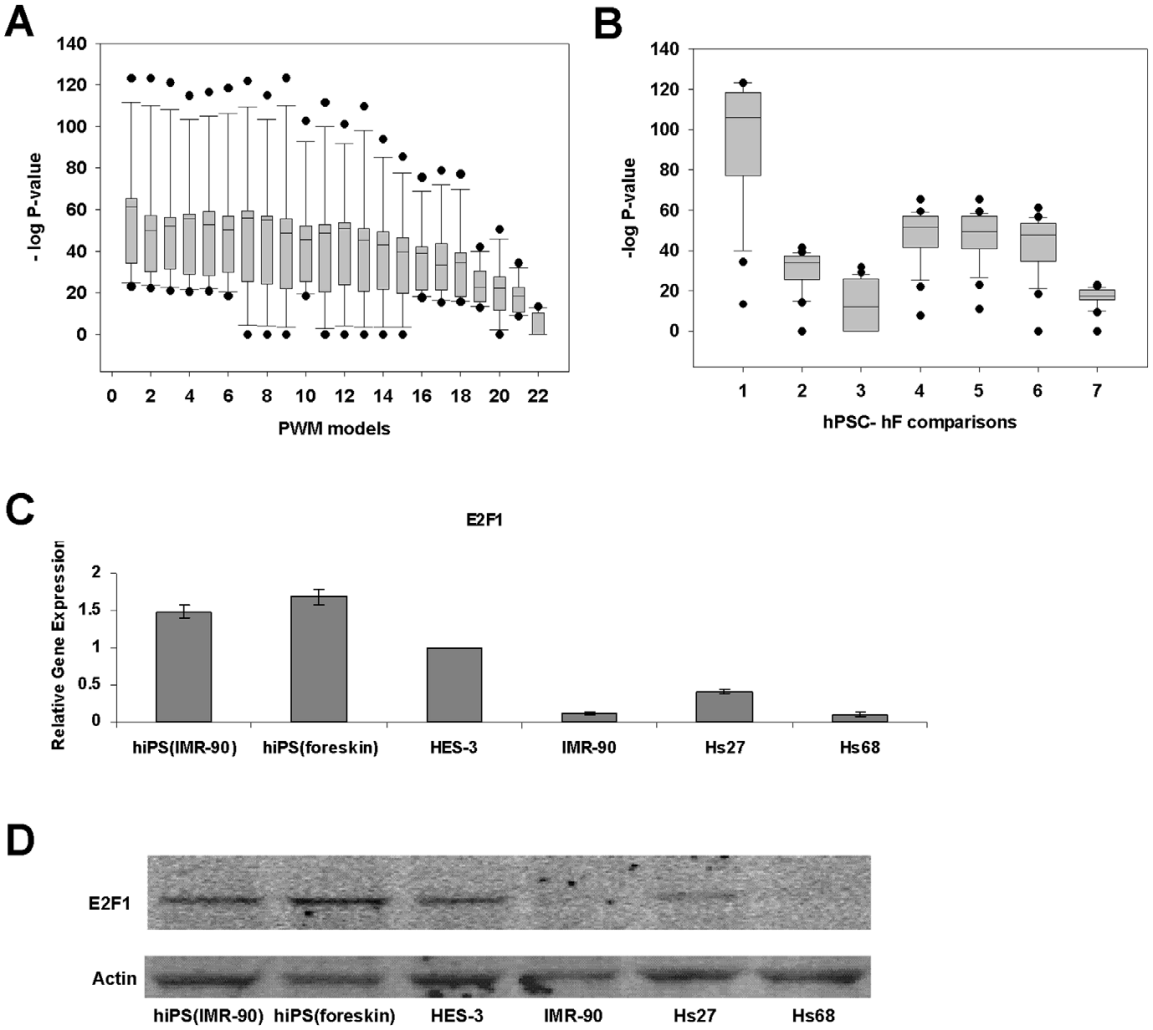
Significant E2F activity and E2F1 differential expression between hPSCs and hFs. (**A**) Box plots depicting statistical significance of TFA for all 22 TRANFAC PWMs, based on 7 hPSC-hF comparisons. (**B**) Box plots summarizing statistical significance of TFA for 7 hPSC- hF comparisons, based on 22 PWMs. Median −log_10_(P-value) is greater than 10 for most hPSC-hF comparisons. The whiskers represent 10^th^ and 90^th^ percentiles while the circles outside them are outliers. (**C**) Quantitative Real-time PCR analysis of gene expression in hPSCs [iPS(IMR90), iPS(foreskin) and HES-3] and hFs [IMR-90, Hs27 and Hs68]. Gene expression was normalized to that of GAPDH and expressed as fold change relative to HES-3. The values shown are mean ± SD of a representative experiment performed in triplicate and repeated twice for each biological replicate (cell line). (**D**) Verification of up-regulation of the TF in hPSCs compared to hFs. Actin served as loading control.

To accelerate evaluation of TFA in large datasets, an algorithm to compute the statistical enrichment of high target propensity genes in global sets of differentially expressed genes was developed (Figure S1), based on an efficient dynamic programming procedure. From a screening of 594 PWMs which model the preferred DNA-binding sequences of TF candidates, those of E2F result in consistently high statistical significance for up-regulated genes in every hPSC-hF comparison (Figures 2A and 2B). This is in contrast to the lack of evidence for down-regulated genes (median −log P-value = 0.0). Hence, E2F target genes are dominantly up-regulated in hPSCs. This is in good agreement with a recent study which revealed that the role of E2F1-3 is switched from an activator in progenitor cells to a repressor in differentiating cells [17], implying up-regulated target genes in progenitor cells.

Interestingly, there are clues pointing to expanded functions of E2F-activated genes, beside their specific canonical roles related to proliferation. Firstly, E2F-responsive genes responded to cell-cycle periodicity in differentiated cells but the expressions of many such genes in ES cells are cell cycle-independent [27]. Further support for its role in hPSC functions comes from the fact that the localization of pRb on E2F-responsive promoters facilitates replicative senescence [28], indicating that E2F may regulate replicative senescence in these cells.

### E2F further regulates its canonical functions in hPSCs compared to differentiated fibroblasts

We further investigated E2F1 activity, which is experimentally supported by E2F1 up-regulation in hPSCs over hFs at the gene expression (>2.5 time up-regulation) and protein levels by quantitative real-time PCR and Western Blot analyses respectively (Figure 2C–D). As E2F may regulate key phenotypic differences between hPSCs and hFs, the next step is to characterize the functions of target genes between these cells. To do so, the expression profile of potential E2F-regulated genes was first explored from a plot of differential expression (score) vs target propensity ranking (Figure S2A) at a genome-wide level, which showed a clear relationship between them, i.e., large differential expression values for high target propensity rank. For a complex transcriptional network, the correlation is considered large (Spearman's Rho = 0.168; two-tailed P-value = 1.0E-97) and significant enough to recover canonically regulated gene groups as described later. If the genes with both high target propensity and differential expression represent true E2F targets, their expression profiles should be similar to E2F's among various hPSC-hF comparisons. We quantified this similarity using two metrics, the dot product and covariance, and observed their increase with higher target propensity rank (Figure S2B–C). Clearly, gene regulation by E2F is detectable, resulting in the distinctive profiles of high target propensity genes. Motivated by this discovery, a novel method, TF target-cohort analysis, was developed to identify functional gene groups regulated by E2F. Unlike other gene group analyses which only consider their expression changes [29], our method evaluates the effect of TFs on gene expression using binding site data. In our study, it detects E2F regulation by testing if the expression properties of high target propensity genes are significant compared to others in the group, through random assignment of expression profiles from the experimental data (Figure S2D–E). Subsequently, we could identify E2F target-cohorts (genes) that have (a) high target propensity, (b) significantly higher differential expression, and (c) similarities to E2F expression profiles, in comparison to other gene members. As such, these genes are enriched in true targets.

Target-cohort analysis showed much more diverse functional regulation by E2F than previously identified. Figure S3A shows the distribution of the P-values for the tested functional groups. A balance between the coverage of E2F functions and the stringency of q-value cut-off could be adjusted by choosing a value of 0.267 for the latter (See ‘significant tests vs q-value cut-off’ plot of FDR analysis based on the method of Storey and Tibshirani [30] in Data S1). It indicated that about 73% of uncovered groups were truly E2F-regulated (True discovery rate = 1-FDR = 0.733, where FDR or false discovery rate = 0.267). We speculated that genes are mostly regulated according to their specific molecular functions which resulted in good agreement between all differential expression-associated metrics, compared to other gene ontologies (Figure S3B). With an eye to recover canonical as well as novel functions of E2F, identified gene groups were classified into eight broad functional categories (Figure S3C). They include signaling, energy generation, transports, protein/lipid/carbohydrate metabolism, adhesion, oncogene-related, development-related and cell cycle-related. Only the last three categories were known canonical functions of E2F. In discussing notable gene groups from the new categories in the next section, we place more emphasis on larger groups (more than 10 members if there is no supportive biological information on their relevance) as they are less influenced by outliers [29], and if supported by differential expression metric and one similarity score. The findings were also carefully augmented with biological information from the literature to mitigate the somewhat large q-value threshold used, noting that target-cohort analysis is designed to report conservative P-values (Supporting Information S1).

We recovered canonical functions of E2F in the form of its targeted gene groups as discussed in Supporting Information S1. These were primarily associated with cellular multiplication and growth, apoptosis as well as differentiation, and can therefore disrupt self-renewal if inappropriately regulated. It therefore implied that the increased expression of E2F and its regulatory activities in hPSCs indeed mediate self-renewal. Interestingly, the other broad categories of E2F functions though largely unknown, can also be interpreted with similar importance as discussed below.

### E2F significantly regulates self-renewal signaling in hPSCs

One of the largest categories of gene groups detected with E2F target-cohorts was signaling pathways and related gene groups (Figure S3C), prompting us to look in more detail for evidence of this much uncharacterized global regulation of signaling in hPSCs by a TF. The list of pathways includes quite a number active in stem cells: WNT, FGF, EGF, Hedgehog, p38 MAPK and FAS. Of high interest with respect to hPSCs, Wnt signaling promotes cell proliferation [31], [32], participates directly in mitosis [33]–[35], maintains pluripotency [10], [36] and enhances somatic cell reprogramming [10]. Similarly, FGF2 signaling promotes proliferation and suppresses apoptosis of hESCs; its suppression enhances differentiation [12], [37]. Thus, a confirmation that E2F regulates component genes in these pathways would suggest such a novel modulation of self-renewal.

**Figure 3:**
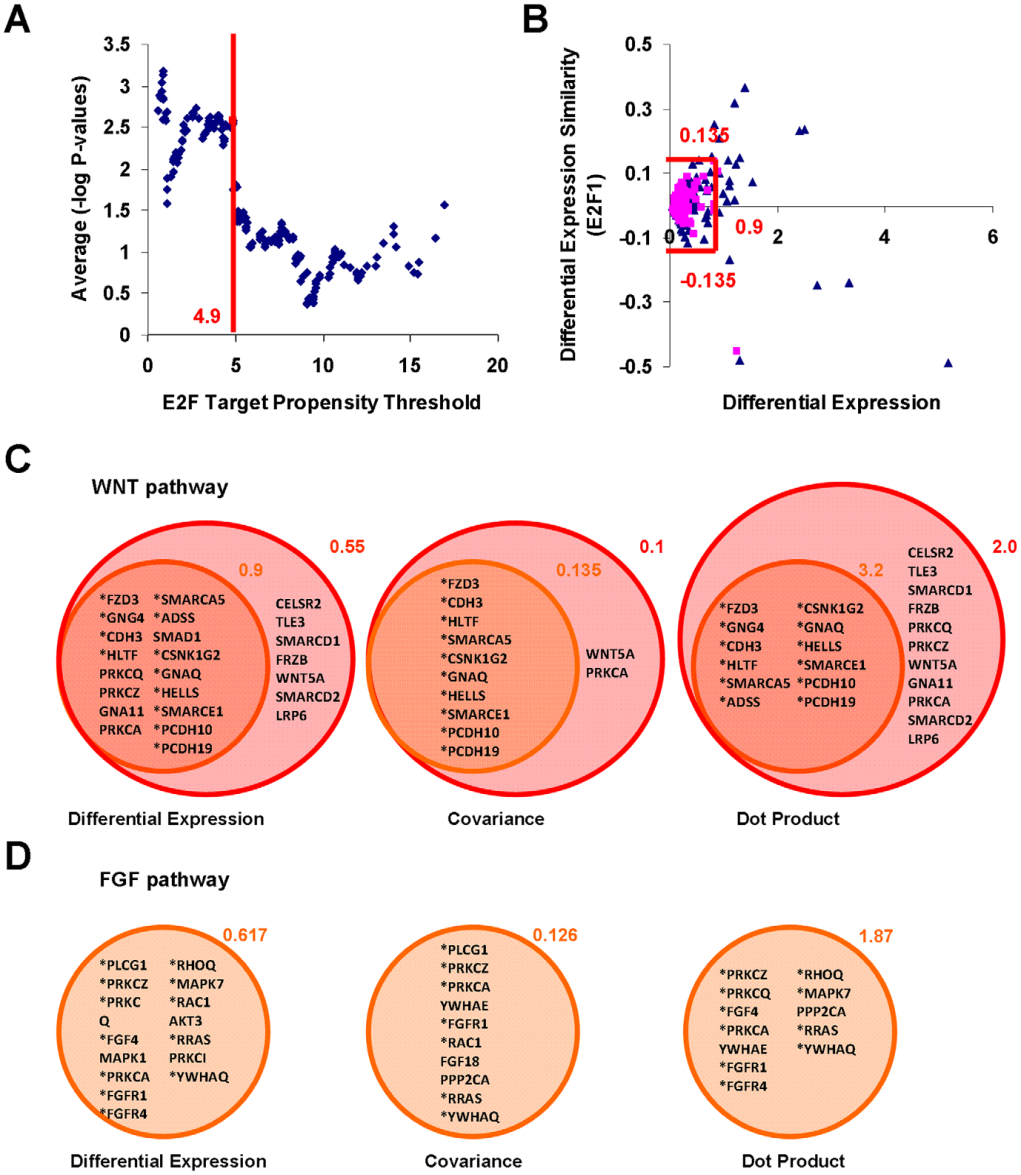
Identification of target-cohorts in WNT and FGF self-renewal pathways. (**A**) Target propensity separation threshold used to separate genes above and below E2F target propensity thresholds. The P-values at each target propensity threshold for various measures of differential expression profiles is computed during target-cohort analysis. The average P-value at each target propensity threshold is used to determine the optimal target propensity threshold to separate high and low target propensity genes. As target propensity threshold decreased to 4.9, statistical significance increases sharply before plateau-ing off. This is called ‘target propensity separation threshold’. (**B**) Differential expression similarity with E2F1 (covariance measure) is plotted against the differential expression scores for genes below (***pink squares***) and above (***blue triangles***) separation threshold. For most genes below separation threshold (with the exception of one outlier), |covariance|<0.135 while differential expression <0.9. For genes above separation threshold, those with |covariance|>0.135 and differential expression >0.9 (***outside red box***), are called target-cohorts, and likely to be enriched in target genes. (**C**) E2F target-cohorts identified in WNT pathway using separation threshold = 4.9 (red circle) and 4.0 (***black circle***). For separation threshold = 4.9, target-cohorts are genes marked with an ‘*’ and have differential expression >0.9 and either |dot product|>3.2 or |covariance|>0.135. To explore more genes for trans-activation by E2F, target propensity separation threshold is lowered to 4.0 with the resulting criteria to identify target-cohorts being differential expression >0.55 and either |dot product|>2.0 or |covariance|>0.1. (**D**) Similar to the procedure highlighted in (**A**) and (**B**), target-cohorts in FGF pathways were identified using separation threshold = 5.1, with differential expression >0.617 and either |covariance|>0.126 or |dot product|>1.87.

**Figure 4:**
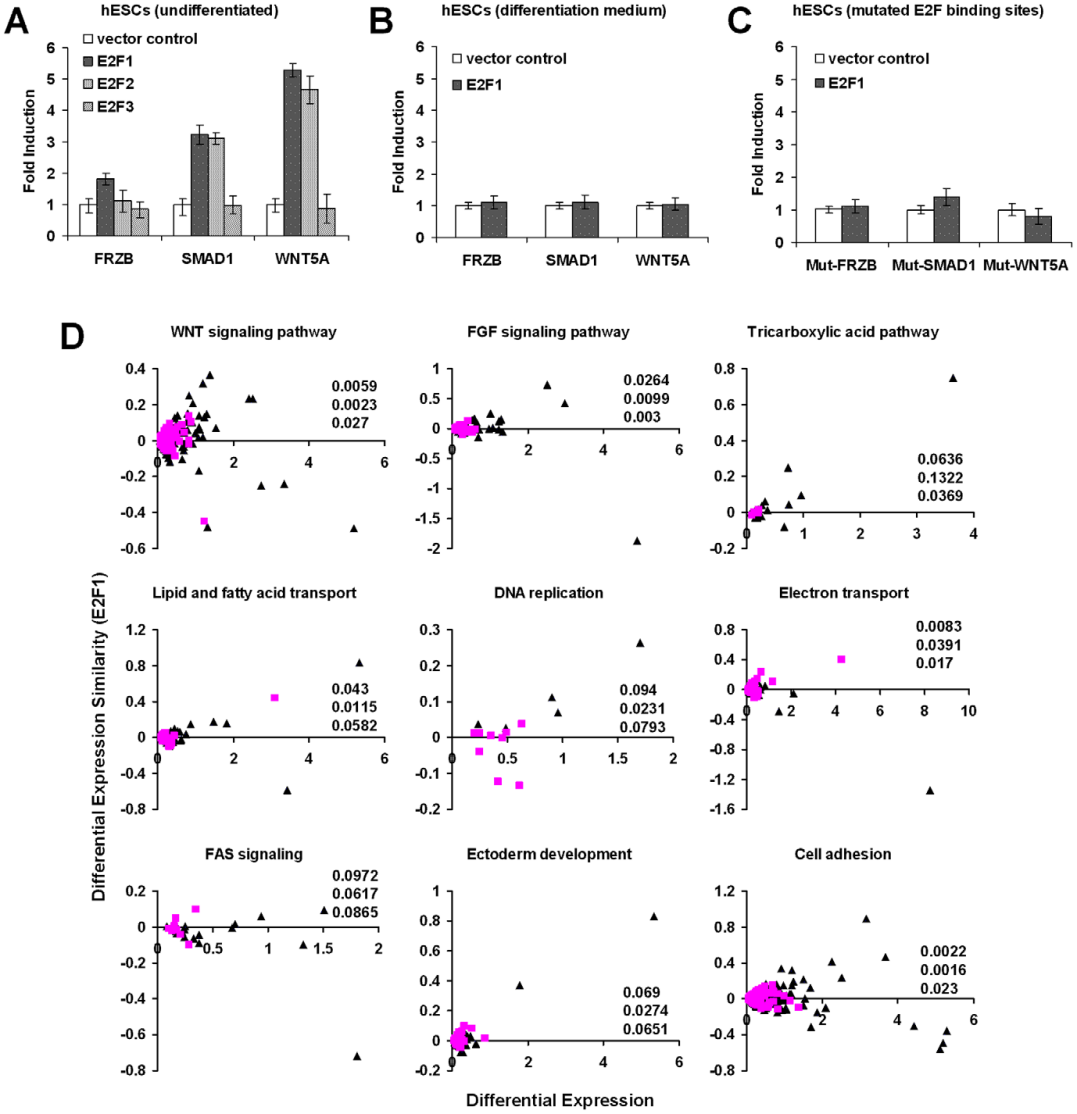
Validation and increased differential expression scores of target-cohorts in various new and canonical E2F functions. Luciferase activity of wild-type promoter-reporter constructs (WNT components- FRZB, SMAD1 and WNT5A) in (**A**) hESCs, (**B**) in hESCs cultured with differentiation medium, and (**C**) in hESCs with E2F binding sites mutated. (a–c) Fold inductions using E2F expression vectors are in comparison to that of empty vector. All luciferase activities were measured relative to the renilla luciferase internal control. Data are illustrated as mean ± SD of a representative experiment performed in triplicate and repeated twice. (**D**) Representative gene groups in functional categories identified by target-cohort analysis. The X-axis represents differential expression score while the Y-axis presents the similarity with E2F1 (covariance measure). Overall, high target propensity genes (triangles) have values more dispersed from the origin, compared to low target propensity genes (squares), implying higher differential expression scores and similarities with E2F1. Corresponding P-values were displayed as a column in the order [differential expression, dot Product, covariance].

To investigate direct E2F regulation of some of these genes, a set of target-cohort in the WNT pathway were selected according to the criteria detailed in Figure 3. Again, target-cohorts have high target propensity with significantly higher differential expression score and similarities to E2F expression profiles, compared to other genes in the group. Many of these identified genes were shown to be differentially expressed between hPSCs and hFs using real-time polymerase chain reaction (PCR) (Figure S4), and their tentative self-renewal associated functions are described in Table S3. Three shortlisted genes namely, WNT5A, FZRB and SMAD1, showed a significant increase in the luciferase activities of their promoter-reporter constructs compared to control vector when assessed for trans-activation by E2F expression vectors in hESCs (Figure 4A). In contrast, there was no increased activity when the hESCs were cultured in a differentiation medium (Figure 4B), or when the E2F binding sites were mutated in the promoter vectors of tested genes (Figure 4C). In all, the results clearly confirm that an elevated E2F level can result in the up-regulation of its target genes in the self-renewal associated WNT pathway, spanning extracellular signaling, signal reception as well as downstream execution of expression regulation, compared to differentiating cells. As activated SMAD1 regulates differentiation and self-renewal via BMP signaling [12], E2F control of its expression as demonstrated by our trans-activation experiments (Figure 4), influences self-renewal. On the other hand, the role of non-canonical WNT5A and FZRB remains to be elucidated.

The myriad of signaling-related gene groups with target-cohorts supports the notion that E2F might coordinate interactions among major pathways. For example, the Ras/Raf/MEK/ERK pathway was theoretically proposed to negatively feedback into the apoptotic machinery, and hence prevents apoptosis triggered by a self-feeding E2F1 [38]. Interestingly, ERK1 and Ras were suggested to be E2F target genes [39], implying that E2F transcriptional regulation of these genes modulates the intensity of Ras/Raf/MEK/ERK feedback loop, thereby regulating the balance between proliferation/differentiation and apoptosis. E2F transcriptional regulation of such component genes in feedback loops is likely to be a common theme among various functional processes in hPSCs.

### E2F regulates metabolism of hPSCs

Another major finding of our work is the previously unknown role of E2F in directly targeting broad classes of metabolism in hPSCs. Interestingly, we found that metabolic gene groups (spanning energy generation, transports and protein/lipid/carbohydrate metabolism) account for almost 1/3 of all uncovered gene groups, outnumbering canonical gene groups in the broad functional categories of oncogenes, development and cell cycle (Figure S3C). Our result clearly showed that metabolism considered in its entirety, is significantly regulated by E2F. In retrospect, this category together with signaling-related gene groups may help explain the under-representation of known E2F targets in their estimated number from cellular binding-site profiling studies [40], [41].

E2F's direct regulation of metabolism may support the high proliferation rate of hPSCs which imposes heavy demands on energy generation and biomass production compared to differentiated cells [42]. This is supported by the role of E2F1 acting as a switch between the glycolytic mode during proliferation and the oxidative phosphorylation under dormant or stressful conditions [43]. Target-cohorts were detected in functional groups related to energy generation (e.g. tricarboxylic acid pathway and electron transport chains), fatty acid metabolism, lipid and fatty acid transport, and exocytosis. Some of them are reported previously. For example, human cytochrome c_1_, whose gene is regulated by E2F [44], participates in an electron transport chain and the mitochondria pathway of apoptosis whereas a similar set of electron transport-related genes (COX8, CYB5-M, CYP51A1, FDXR and SUCLG1) were found to be E2F4-bound in cancer cell lines [45]. In addition, four transporter genes were identified to be potential E2F targets [39]. As illustrated selectively in Figure 4D, metabolic gene groups identified from target-cohort analysis have genes with high target propensity (triangles) significantly more dispersed away from the origin when drawn in differential expression profile space, compared to low target propensity genes (squares). This observation indicates the presence of differentially expressed genes targeted by E2F. Similar results were found for WNT and FGF pathways which illustrated the biological significance of predictions using our analytical framework.

## Discussion

In this study, a differential E2F regulation of its canonical (cell cycle, apoptosis and differentiation programs) and associated functions has been newly demonstrated in hPSCs. We further found that E2F is a novel regulator of the global signaling and metabolic networks in pluripotent stem cells which include WNT and FGF as well as energy generation, fatty acid metabolism and transport pathways. All in all, the regulation of these functions represents a more complete picture of different self-renewal capabilities, indicating a definitive, central role for the TFs in these cells (Figures 3 and 4). The ability of our integrated bioinformatics pipeline to uncover the roles of E2F was due to the relevance of our target propensity model, which is based on core and proximal promoter enrichment of putative binding sites [39], well-supported by its genomic binding profiles and known participation in basal transcription (Supporting Information S1). While we emphasize the usefulness of exploratory TFA screening, complementary criteria in discerning candidate priorities and caveats are described in Figure S1. Further down the pipeline, similar approaches to target-cohort analysis are envisioned to provide a rapid computational snapshot of E2F targeted functions temporally such as during development or oncological progression.

**Figure 5:**
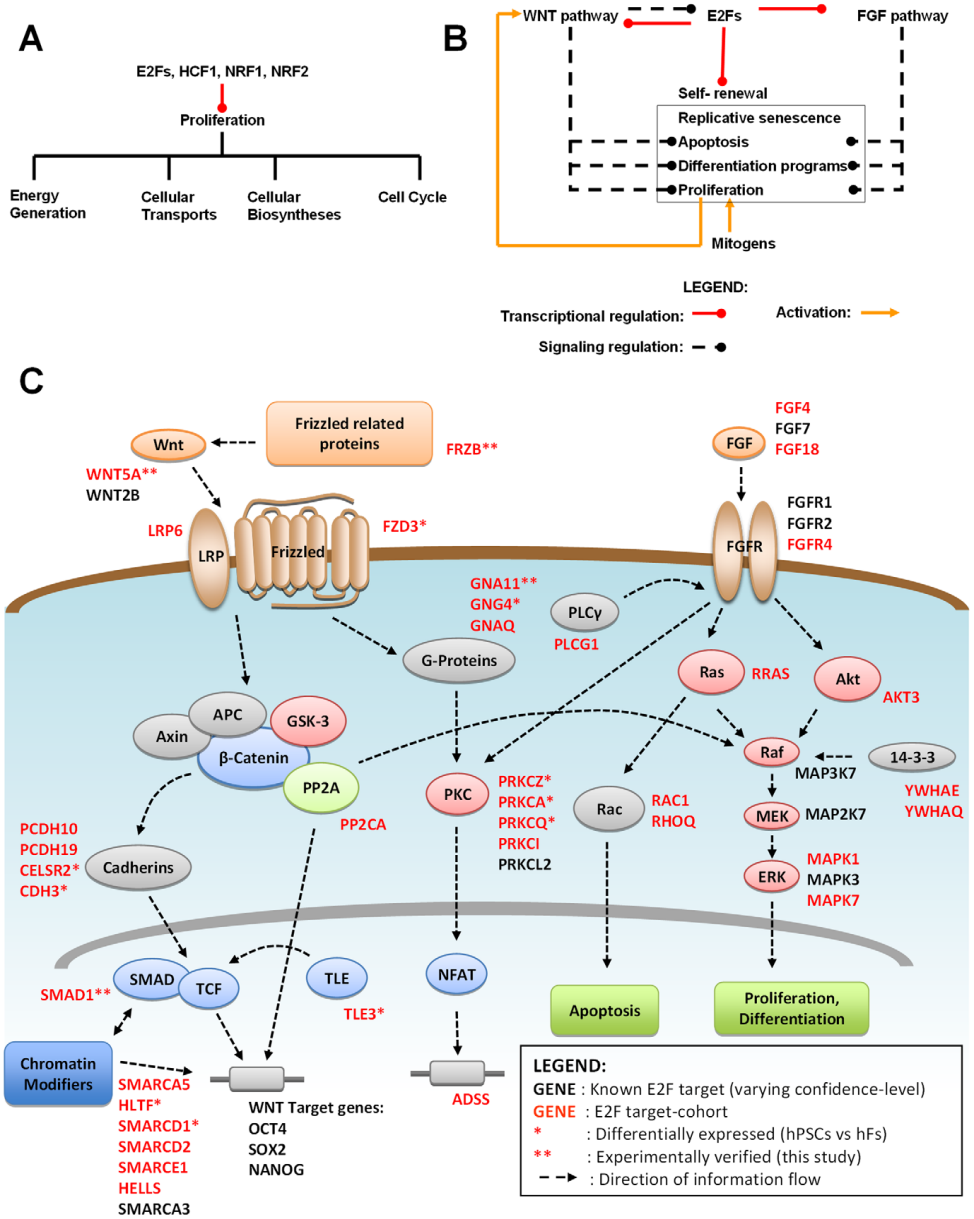
Interplay between E2F and other cellular regulators in the direct regulation of self-renewal associated functions. (**A**) Co-regulated functions of E2F [14–16, this work], HCF1 [46], [47], NRF1/2 [48], [49] are associated with cell proliferation (**B**) E2F, WNT and FGF activities are deeply integrated in a self-renewal module of interplay between gene regulation and signal transduction. Besides directly targeting genes with the canonical functions of proliferation, differentiation and apoptosis, E2F transcriptionally regulate the component genes of these signaling pathways with the same functions. Remarkably, WNT pathway is engaged in a positive feedback with cell cycle progression [51]. With mitogens and intracellular regulators (such as E2F) driving cell cycle progression in hPSCs, a self-feeding state of high cell-proliferation may be programmed into hPSCs via the WNT pathway. (**C**) Key components of WNT and FGF pathway are regulated by E2F. The ovals and blocks represent key components of the pathways while the black arrows depict direction of information flow. E2F target-cohorts (red) and known target genes (black) encoding signaling components, are listed beside them. If a gene is both a known target and a target-cohort, it is colored red. Known targets include WNT2B, SMARCA3, SMARCA5, RRAS, MAP2K7, YHWAE (BIND database), FGF1 [54], FGF2 [53], FGF7, PRKCL2, MAP3K7, MAPK3 (ERK1), [39], [58], SOX2, OCT4, NANOG [55]. Genes denoted with ‘**’ are experimentally verified in this study to be regulated by E2F (Luciferase-based assay) while those with ‘*’ are experimentally shown to be differentially expressed (RT-QPCR).

Further support for the direct role of E2F in metabolism comes from its association with other TFs with similar functions. It was found that gene promoters bounded by the self renewal-associated complex, HCF1-Ronin, are associated with E2F1 binding in mouse ESCs [46]. Target genes of the complex are involved in protein transport, metabolism and modification, as well as oxidative phosphorylation and mTOR signaling regulating cellular growth and metabolism. It is further known that E2F1/3/4 tether HCF1 to DNA, allowing HCF1 to recruit epigenetic regulators, leading to the transcriptional activation of cell-cycle controlled genes and the promotion of cell proliferation [47]. In addition, NRF1/2, the other differentially-expressed candidate regulators identified from our screening (Figure S5), are also associated with E2F and similarly implicated in protein transports, energy generation, mitochondrial DNA replication and cell cycle control [48], [49]. NRF2-β, in particular, was shown to interact with HCF1 and E2F1 in yeast two-hybrid experiments [46]. The hypothesis that E2F and NRF1/2 co-regulate genes in hPSCs is underscored by the fact that a number of E2F targets involved in mitochondria biogenesis, mitosis, DNA replication and cytokinesis are transcriptionally regulated by NRF1/2 [45], [48], [49]. The association of E2F with these metabolic regulators as summarized in Figure 5A further lends support to the hypothesis that the transcription factor regulates metabolism.

We also looked for empirical interactions between E2F and WNT, FGF activities that mediate self-renewal associated functions. Here, we emphasize the feedback relationship between E2F and WNT activities. Previous works showed that Wnt-1 induces downstream E2F1 expression [50] while we demonstrated WNT5A is a target gene of E2F1 and E2F2 (Figure 4A). The mechanisms involved may be highly elaborate, as illustrated by the discovery that cell cycle progression (such as E2F-driven) can trigger a positive feedback with Wnt signaling [51]. The resultant increase in WNT signaling may further induce E2F expression to drive cell proliferation (Figure 5B).

For FGF pathway, its role with respect to self-renewal can be attributed, in part, to mechanisms such as SMAD1 activity antagonism and PI3K/AKT signaling [12], both found to be E2F-mediated in our work (See Figure 4, and broad functional categories of E2F target-cohorts in Data S1, respectively). Along the same line, E2F1 was found to trigger AKT activation by directly effecting the transcription of the adaptor protein Gab2 [52]. Further upstream, FGFR1, a key receptor for the canonical self-renewal factor FGF2, was identified to be a target-cohort in our study (Figure 3). This was supported by the findings that E2F1-3 directly activate FGFR1-2 gene expression [53], [54] promoting mitosis. Figures 5A and 5B summarize our respective results on the co-regulation of various aspects of proliferation by E2F and a core self-renewal module in hPSCs depicting the interplay between the TFs and WNT/FGF pathways; the hypothesis that E2F directly control the expression of these self-renewal pathways is detailed in Figure 5C.

Moving forward, the proposed functions of the E2F family should be viewed as an invitation to clarify the roles (if any) of its individual members in hPSCs, after highlighting their collective importance. Our work suggests that their understanding may bring about new perspectives on the characterization, modulation and engineering of the self renewal/metabolism phenotype with implication for the therapeutic application of stem cells. For example, the propagation of hPSCs in culture medium may be affected by differences in intrinsic E2F level such as between hiPSCs and hESCs (Figure 2C), due to the influence of the TF on the strength of WNT and FGF signaling.

Finally, we also highlight a number of studies that elucidate the role of E2F with respect to somatic cell reprogramming. Since promotion of proliferation and Wnt/β-catenin/Tcf3 pathway activation enhance reprogramming [10], E2F potentiating these two functions should have the same effect. It may also do so through direct transcriptional activation of Oct4, Sox2, Nanog and Klf4 [55]. Furthermore, the suppression of p53 and its apoptotic activity were found to promote proliferation, leading to increased reprogramming efficiency [56], [57]. As such, E2F which modulate expression of genes involved in the induction and execution of apoptotic processes (14–16; this work), may further influence reprogramming.

## Supporting Information

Figure S1 **Chi-square statistics as a proxy to detect TFA.** **Chi-square statistics** is used to represent gene number enrichment in the overlap between subsets of high ranking (1) SAM score and (2) target propensity. We incorporate the Yate's correction into the statistics and compute if the expected overlap is greater than 10 genes. This ensures that its value is not unreliably large for small expected overlap. In addition, we only evaluated cases where the observed overlap number is greater than its expected number. Subjected to these constraints, the **maximum chi-square** value among all pairs of rank thresholds is the proxy test-statistic to detect TFA, i.e., direct gene regulation by the TF at a systems-level. However, true targets of a TF may not have the largest differential expression scores. This is taken into account by incrementally removing the highest-ranking genes from the SAM score subset that give the maximum chi-square earlier, and re-computing the test statistic. **P-value extrapolation:** Statistical significance is evaluated by randomizing the gene rankings and re-computing the maximum Chi-Square to obtain the sampled distribution of 10000 values. A linear relationship (R^2^>0.99), which exists between sampled maximum Chi-Squares and their corresponding −log P-values, is used to extrapolate the statistical significance of observed maximum Chi-Square. **Other criteria**: Candidate TFs are given higher priority for differential expression evaluation if (a) their PWMs have high binding specificities, (b) their PWMs are annotated from large set bound sequences, and (c) their known genomic binding loci used in gene regulation are largely confined to the core and proximal promoters. By requiring differential expression of candidate TF as the last stringent criterion, we believe shortlisted candidates are promising as follow-ups. Note that the last criterion is likely to bypass potential TFs that are activated in hPSCs through post-translational mechanisms and not via changes in expressions.
(TIF)

Figure S2 **Basic idea behind target-cohort analysis.** The analysis make use of the fact that there is a significant global increase in (**A**) differential expression score, as well as score similarities with E2F1 using (**B**) dot product metric and (**C**) covariance metric (based on a 250-genes moving average) vs target propensity ranking. The trends are attributed to the presence of E2F target genes which increase the differential expression scores of high target propensity genes. Similarly, target-cohort analysis identifies significantly-regulated gene groups by looking out for these trends. (**D**) Specifically, a non-significant gene group sampled randomly have similar values for both high (***triangles***) and low (***squares***) target propensity genes while (**E**) a significant gene group has higher average values for high target propensity genes (***triangles***), compared to low target propensity genes (***squares***), as visually described in the diagrams. In general, scores were sampled from the global microarray data without replacement.
(TIF)

Figure S3 **Summary result of target-cohort analysis.** (**A**) P-value histograms from target-cohort analysis evaluating E2F regulation of gene groups. A P-value = 0.1 corresponds to the q-value cut-off = 0.267 for differential expression score. (**B**) Venn diagram showing overlaps numbers of identified gene groups using differential expression score and score similarities. Interestingly, gene groups detected using differential expression score and score similarities show the greatest overlap for molecular functions with only 8 (15%) detected by any one metric, followed by pathways (23%) and biological processes (34.3%). (**C**) Identified gene groups according to broad functional categories with number of gene groups in brackets.
(TIF)

Figure S4 **Gene expression of E2F target-cohorts in hPSCs compared to hFs.** Quantitative Real-time PCR analysis of target-cohorts in hPSCs [iPS(IMR90), iPS(foreskin) and HES-3] and hFs [IMR-90, Hs27 and Hs68]. Gene expression was normalized to that of GAPDH and expressed as fold change relative to HES-3. The values shown are mean ± SD of technical triplicate for various biological replicates (cell lines). A large proportion of tested target-cohorts are significantly differentially expressed between hPSCs and hFs.
(TIF)

Figure S5 **Significant NRF1, NRF2 activities in hPSCs compared to hFs.** (**A**) NRF1 and NRF2 showed high statistical significance during TFA screening for 7 hPSC-hF comparisons. (**B–E**) Gene expression analysis by quantitative real-time PCR for NRF1 and NRF2 subunits (GABPA, GABPB1, GABPB2) in various biological replicates (cell lines). NRF1 and GABP2 showed significant differential expression between hPSCs and hFs. (**F**) NRF1 showed differential protein expression between hPSCs and hFs by Western Blot analysis.
(TIF)

Table S1 **E2F motifs (red) and corresponding mutations (bold) on FRZB, SMAD1 and WNT5A promoters used in this study.**
(DOC)

Table S2 **Primers used for gene expression validation (real-time PCR analysis) of E2F1 and WNT-associated target-cohorts.**
(DOC)

Table S3 **Tentative self-renewal associated functions regulated by E2F target-cohorts in WNT and FGF pathways.** Some genes in the WNT pathway as listed in Figure 3 are cited under FGF pathway instead, due to dual memberships.
(DOC)

Supporting Information S1 (DOC)

Data S1 (PDF)
