# Supplementary figures and images for: Integrated Transcriptome and Binding Sites Analysis Implicates E2F in the Regulation of Self-Renewal in Human Pluripotent Stem Cells
Source: PLoS One. 2011 Nov 4;6(11):e27231. doi: 10.1371/journal.pone.0027231 (PMC3208628; doi:10.1371/journal.pone.0027231)

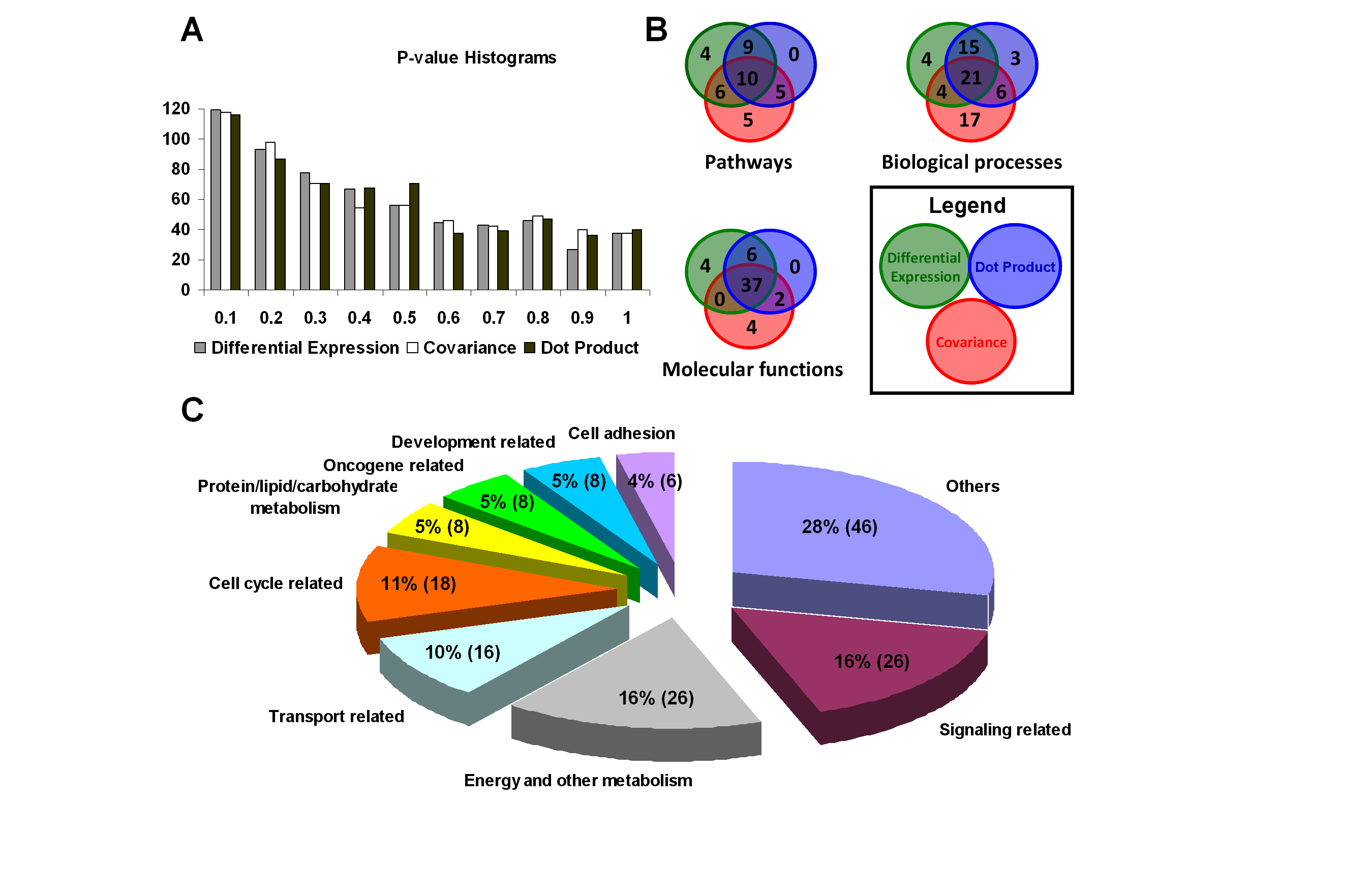

Supplement: Figure S3 — Summary result of target-cohort analysis. (A) P-value histograms from target-cohort analysis evaluating E2F regulation of gene groups. A P-value = 0.1 corresponds to the q-value cut-off = 0.267 for differential expression score. (B) Venn diagram showing overlaps numbers of identified gene groups using differential expression score and score similarities. Interestingly, gene groups detected using differential expression score and score similarities show the greatest overlap for molecular functions with only 8 (15%) detected by any one metric, followed by pathways (23%) and biological processes (34.3%). (C) Identified gene groups according to broad functional categories with number of gene groups in brackets. (TIF) [file pone.0027231.s003.tif]
