# Supplementary material for: Integrated Transcriptome and Binding Sites Analysis Implicates E2F in the Regulation of Self-Renewal in Human Pluripotent Stem Cells
Source: PLoS One. 2011 Nov 4;6(11):e27231. doi: 10.1371/journal.pone.0027231 (PMC3208628; doi:10.1371/journal.pone.0027231)
Supplement: Table S1 — E2F motifs (red) and corresponding mutations (bold) on FRZB, SMAD1 and WNT5A promoters used in this study. (DOC) [file pone.0027231.s006.doc]

**Table S1.** **E2F motifs (red) and corresponding mutations (bold) on FRZB, SMAD1 and WNT5A promoters used in this study**

| **> E2F motifs on FRZB promoter (Wild-type) hg18 chr2: 183,439,517-183,439,711**  GCAGACCATGATCCCGGCAGGATGGGGCAGGGTGCAGCCGCGCAGTGGACGCCAAAAGGCCCGCTCCGCCGTCTCCGCCTCCCCCGCTGCAAGTGGACACAAGGATCTGGGAGCTTCTCCTCCCCCGGCAATCACCGCTTCCTTGGATCAAATTCCCCCAATGGGGTCCCACGAGCTTTACCGAGCTCCAGCCAC |
| --- |
| **> E2F motifs on FRZB promoter (Mutated)**  GCAGACCATGATCC**TATT**AGGATGGGGCAGGGTGCAGCCGCGCAGTGGACGCCAAAAGGCCCGCTCCGCCGTCTCCGCCTCCCCCGCTGCAAGTGGACACAAGGATCTGGGAGCTTCTCCTCC**AATA**GCAATCACCGCTTCCTTGGATCAAATTCCCCCAATGGGGTCCCACGAGCTTTACCGAGCTCCAGCCAC |
| **> E2F motifs on SMAD1 promoter (Wild-type) hg18 chr4: 146,622,152-146,622,606** GGGTTAGGAAAGCCTGAACGCCGCCACACTGTTTTTTCTCCAGGAAGAAACGGGGCGTGGGGCTGCCGGTAGAAGGGGGTGGCTCCGGCCGCTCCCCCGCGCCGTCCTCCGGCCCCGGCCGCGCTGCGCTCACGCCGGCCGGGCCGGGAATTTGGAGAGGATCCCTGGTCGCGCGGCAGCGGCGGCGGCGCGCGGGTGAGCGGGTGAGCGTGTGAGCGGGCGGGCGGGCAGGCGAGTGCGCCGGGTATTGGCAGCTGAGGAGTGGAGGCTGGGCAGCTCCGACTCCCTGACGCCAGCGCGACCAGATCAATCCAGGCTCCAGGAGAAAGCAGGCGGGCGGGCGGAGAAAGGAGAGGCCGAGCGGCTCAACCCGGGCCGAGGCTCGGGGAGCGGAGAGTGGCGCAGCGCCCGGCCGTCCGGACCCGGGCCGCGAGACCCCGCTCGCCCGGCCACTC |
| **> E2F motifs on SMAD1 promoter (Mutated)**  GGGTTAGGAAAGCCTGAAC**AATA**CCACACTGTTTTTTCTCCAGGAAGAAACGGGGCGTGGGGCTGCCGGTAGAAGGGGGTGGCTCCGGCCGCTCCCCCGCGCCGTCCTCCGGCCCCGGCCGCGCTGCGCTCACGCCGGCCGG**AATA**GGAATTTGGAGAGGATCCCTGGTCGCGCGGCAGCGGCGGCGGCGCGCGGGTGAGCGGGTGAGCGTGTGAGCGGGCGGGCGGGCAGGCGAGTGCGCCGGGTATTGGCAGCTGAGGAGTGGAGGCTGGGCAGCTCCGACTCCCTGACGCCAGCGCGACCAGATCAATCCAGGCTCCAGGAGAAAGCAGGCGGGCGGGCGGAGAAAGGAGAGGCCGAGCGGCTCAACCCGGGCCGAGGCTCGGGGAGCGGAGAGTGGCGCAGCGCCCGGCCGTCCGGACCCGG**AATA**CGAGACCCCGCTCGCCCGGCCACTC |
| **> E2F motifs on WNT5A promoter (Wild-type) hg18 chr3: 55,496,242-55,496,631**  GAGTTGGGGCAGAGCTGGGATGCGCCCAGGAATGGAGGGGGCGCGGACGCGCGCGAGCCGGCAGCAAGGGCAGGGCCTGGTCGGGGCGCAACTAGGGAGCCGCCGGTCCGGCGAGGGCGCGCAGGCAACTGTTCCACGGAGAGGCGCTCCGTTTCCAACGTCCATCAGCGACGGCGGTAATTAGGGCTTTCCAACCCCAAATGTGGGCGTGATTGTGCAAAAGACCTTACGACAAATAATAATAAAAAGAAATTCTTCACAAGAGGGTGAAAAAAATGTACCACTACTCAACTGTGGCCCGGGGCGGGGGAAGGGGGGCGATCTGTGCGCCCAGGTGCCCCCAGTTCATTCACACCACAGATTCTGCAAACTCTTGGCGGCTCACGCCTC |
| **> E2F motifs on WNT5A promoter (Mutated)**  GAGTTGGGGCAGAGCTGGGATGCGCCCAGGAATGGAGGG**AATA**CGGACG**AATA**CGAGCCGGCAGCAAGGGCAGGGCCTGGTCGGGGCGCAACTAGGGAGCCGCCGGTCCGGCGAGGGCGCGCAGGCAACTGTTCCACGGAGAGGCGCTCCGTTTCCAACGTCCATCAGCGACGGCGGTAATTAGGGCTTTCCAACCCCAAATGTGGGCGTGATTGTGCAAAAGACCTTACGACAAATAATAATAAAAAGAAATTCTTCACAAGAGGGTGAAAAAAATGTACCACTACTCAACTGTGGCCCGG**AATA**GGGGAAGGGGGGCGATCTGTGCGCCCAGGTGCCCCCAGTTCATTCACACCACAGATTCTGCAAACTCTTGG**TATT**TCACGCCTC |
