# Supplementary material for: Integrated Transcriptome and Binding Sites Analysis Implicates E2F in the Regulation of Self-Renewal in Human Pluripotent Stem Cells
Source: PLoS One. 2011 Nov 4;6(11):e27231. doi: 10.1371/journal.pone.0027231 (PMC3208628; doi:10.1371/journal.pone.0027231)
Supplement: Table S2 — Primers used for gene expression validation (real-time PCR analysis) of E2F1 and WNT-associated target-cohorts. (DOC) [file pone.0027231.s007.doc]

**Table S2. Primers used for** gene expression validation (real-time PCR analysis) of E2F1 and WNT-associated target-cohorts.

| **Gene name** | **Sequences** |
| --- | --- |
| E2F1 | F: 5’-CAGCTGGACCACCTGATGAAT-3’  R: 5’-GCAATGCTACGAAGGTCCTGA-3’ |
| FZD3 | F: 5’-TACACGCCCTGCAGTTACAGAG-3’  R: 5’-TTGAGCCGATGAGAACTACTATGC-3’ |
| GNG4 | F: 5’-GAGTTCCCTTAGCCCCCTGA-3’  R: 5’-GTGTTTGCTTCACGGTGTGC-3’ |
| CELSR2 | F: 5’-GGTGCCATTGGCCGAGTAC-3’  R: 5’-GAGTTCATTTCCCCGCTCAA-3’ |
| TLE3 | F: 5’-GCACAGATCATGCCTTTCCTG-3’  R: 5’-CGATGATGGCGTTCAGCTC-3’ |
| CDH3 | F: 5’-TACCCAGGACACCTTCCGAG-3’  R: 5’-TCATCCGTGGCTGTCACCT-3’ |
| HLTF | F: 5’-TTTTCTGAGAAGGACCGACCAG-3’  R: 5’-TGGCCGTAAGAGTTTTACCCAA-3’ |
| SMARCD1 | F: 5’-CTGTATGGGCCAGACAACCAT-3’  R: 5’-CGTACATTCACGT CTCCCGG-3’ |
| FRZB | F: 5’-GCCTCTGCCCTCCACTTAATG-3’  R: 5’-CTCAGCTATAGAGCCTTCCACCA-3’ |
| PRKCQ | F: 5’-AAGCTCTTCGTGCGAGAACC-3’  R: 5’-TTCCGTTCAA GTTCCTCCCA-3’ |
| PRKCZ | F: 5’-CCTGGTCGGATTACACTCCTG-3’  R: 5’-TCTGCCTCTGCATGTGGAAC-3’ |
| ADSS | F: 5’-AAGCAGCTGATGGTATCCAGGA-3’  R: 5’-AGCTTTGGACGAATAAACTGGG-3’ |
| GNA11 | F: 5’-AGTACGAGCAGAACAAGGCCA-3’  R: 5’-CGTACTGATGCTCGAAGGTGG-3’ |
| PRKCA | F: 5’-CAATGCGTCATCAATGTCCC-3’  R: 5’-ACTGTGACATGGAGCTTTTCATCA-3’ |
| SMAD1 | F: 5’-CTGCTCTCCAATGTTAACCGG-3’  R: 5’-TCGGCATACACCTCCCCTC-3’ |
| WNT5A | F: 5’-AAGGGCTCCTACGAGAGTGCT-3’  R: 5’-AGCCAGGTTGTACACCGTCCT-3’ |
| LRP6 | F: 5’-AGACCTCGAGCCGTTGTGG-3’  R: 5’-GACCTCCCGTTCTGTCCCAT-3’ |
