# Supplementary material for: Integrated Transcriptome and Binding Sites Analysis Implicates E2F in the Regulation of Self-Renewal in Human Pluripotent Stem Cells
Source: PLoS One. 2011 Nov 4;6(11):e27231. doi: 10.1371/journal.pone.0027231 (PMC3208628; doi:10.1371/journal.pone.0027231)
Supplement: Table S3 — Tentative self-renewal associated functions regulated by E2F target-cohorts in WNT and FGF pathways. Some genes in the WNT pathway as listed in Figure 3 are cited under FGF pathway instead, due to dual memberships. (DOC) [file pone.0027231.s008.doc]

**Table S3. Tentative self-renewal associated functions regulated by E2F target-cohorts in WNT and FGF pathways.** Some genes in the WNT pathway are listed under FGF pathway as a result of dual memberships

| **WNT pathway** | **Regulated functions (Author curation from GeneCard)** |
| --- | --- |
| FZD3,TLE3,LRP6,WNT5A,FRZB | Wnt/Beta-catenin/TCF signaling |
| GNA11,GNG4,GNAQ | Signal transduction |
| PCDH10,PCDH19,CELSR2 | Plasma membrane component, calcium-dependent cell adhesion |
| CDH3 | Wnt/Beta-catenin/TCF signaling, calcium-dependent cell adhesion |
| SMAD1 | Proliferation, apoptosis, differentiation |
| SMARCD2 | Chromatin remodeling |
| ADSS | De-novo purine nucleotide biosynthesis, alanine, aspartate and glutamate metabolism |
| HLTF, SMARCE1, SMARCA5, SMARCD1 | Differentiation (Chromatin remodeling) |
| HELLS | Proliferation, apoptosis (Chromatin remodeling) |
| CSNK1G2 | Proliferation, DNA replication, nuclear localization, membrane transport |

| **FGF pathway** | **Regulated functions (Author curation from GeneCard)** |
| --- | --- |
| PLCG1 | Proliferation, apoptosis, maintenance of membrane phospholipids |
| PRKCZ | Proliferation, differentiation, apoptosis, cell adhesion |
| PRKCA | Proliferation, cell adhesion |
| PRKCQ | Proliferation |
| YWHAQ,YWHAE, PRKCI | Proliferation, apoptosis |
| RRAS | Nucleocytoplasmic transport, actin cytoskeleton organization, intracellular protein transport |
| RAC1 | Proliferation, cell adhesion, motility, differentiation |
| FGFR1, FGFR4 | Angiogenesis, proliferation, development, differentiation |
| FGF4,FGF18 | Proliferation, apoptosis, development |
| MAPK1, MAPK7 | Proliferation, differentiation, development, apoptosis |
| RHOQ | Glucose uptake |
| AKT3 | Proliferation, differentiation, apoptosis |
| PPP2CA | Proliferation, apoptosis |
