# Supplementary material for: Integrated Transcriptome and Binding Sites Analysis Implicates E2F in the Regulation of Self-Renewal in Human Pluripotent Stem Cells
Source: PLoS One. 2011 Nov 4;6(11):e27231. doi: 10.1371/journal.pone.0027231 (PMC3208628; doi:10.1371/journal.pone.0027231)
Supplement: Supporting Information S1 — (DOC) [file pone.0027231.s009.doc]

**Supporting Information S1**

**Recovery of E2F canonical functions**

The result showed that E2F target-cohorts are involved in cell cycle and other co-regulated functions such as DNA replication and cytokinesis [1] which is consistent with the literature. Expanding the list, our analysis also uncovered E2F cohorts encoding actin-related proteins (such as actin-binding cytoskeletal protein) (See functional categories in Supporting Data S1). Indeed, E2F drives the cell cycle, establish cell cycle checkpoints, regulate cell proliferation rate and coordinate mitotic programs [2]. Some of these programs include DNA recombination, histone syntheses and modifications, centromeric protein synthesis, centrosome duplication as well as chromosome condensation and segregation. The finding that functional groups associated with cell proliferation are regulated in hPSCs compared to hFs, may reflect the higher proliferation rate of hPSCs.

Other detected target-cohorts were in functional groups consistent with the role of E2F in oncogenesis. Known target genes participate in the induction and execution of apoptosis (also identified as target-cohorts), tumor growth and suppression, as well as cell proliferation which include the diverse Myc, Myb and E2F families of TFs [3-5]. Similarly, target-cohorts were related to cell motility, angiogenesis and FAS signaling.

At the organism-level, E2F is involved in developmental programs. Our study found targeted gene groups executing ectoderm development, embryogenesis [3], neurogenesis [6] and oogenesis [7], suggesting their potentiation in hPSCs relative to hFs. The developmental role of E2F can be understood in terms of the temporal and spatial regulation of cell proliferation, apoptosis and differentiation. In addition, genes that are relevant to chromatin packaging and remodeling such as the developmentally important SOX genes (a class of HMG box TFs) were detected to be target-cohorts. Of interest, HMG box TFs were previously reported as E2F targets [8]**.**

**E2F functional motifs enrichment in core and proximal promoters**

An unbiased location analysis of ChIP-chip E2F1 binding sites in Hela cell line revealed that 51% of these sites were localized at the transcription start site (TSS), while 82% falls within 1 kbp of the TSS. Only 4% overlaps the 3’ end of transcripts [9]**.** A similar localization of ChIP-chip E2F4 and E2F6 binding sites in the core promoters were also reported [10]. In addition, the promoters of cell cycle genes (canonical E2F targets) showed an enrichment of binding sites between -400bp to +100bp and peaking very near to the TSS, compared to a typical eukaryote promoter [8]**.** All these indicate functional motif enrichment in core and proximal promoters.

The role of E2F1 in basal transcription further supports the notion of E2F functional binding in core promoters. The TF was shown to be capable of interacting with components of Pre-Initiation Complex (PIC), required for transcription initiation. These include TFIIA, TFIID, TBP (subunit of TFIID) and TFIIH [11-14]. E2F1 may stabilize the binding of TBP to TATA-containing promoters in the first step of PIC assembly beside the recruitment of TBP to TATA-less promoters [8].

Further support for the claim of E2F functional binding in core promoters comes from the genome-wide E2F co-localization with PIC components. E2F1 was found within 1 kbp of 83% of POLR2A (subunit of RNA polymerase II) sites, and within 1kbp of the TSS in 90% of POLR2A-occupied promoters [9]. The genomic binding profiles of E2F1, E2F4 and E2F6 are similar to POLR2A and TAF1 (subunit of TBP) [10]**.** However, it is not known whether the interactions of E2F1 with the basal complex components are conserved among the other E2F.

**Pseudo-algorithm for target-cohort analysis**

1. For each pre-defined gene group, the set of ***I*** unique target propensity thresholds is denoted as ***T* = {*t_i_*: *i*=1, 2, …, *I*-1, *I*}** and ***t*_1_> *t*_2_ > … > *t_I_*_-1_ > *t*_I_**.
2. Member genes in a group were split into two subsets- (a) above or equal to the target propensity threshold and, (b) below the threshold.
3. *Compute score increment between high and low target propensity subsets* **∆*S_i_***. (first column of array A)
4. Obtain ***J*** = 10,000 gene groups of the same size by assigning member genes with scores from randomly-sampled genes in the microarray dataset without replacement. Similarly, *compute score increments between high and low target propensity subsets* **∆*S_i_ ^j^*** for ***j***th sample (in ***j***+1 column of array A)
5. P-value of ∆***S*** for threshold ***t_i_*** = P (∆***S_i_***) = **P*_i_* = (# {∆*S_i_ ^j^*: ∆*S_i_ ^j^* ≥ ∆*S_i_*} +1) / *J***. (in column one of array B).
6. P-value of ∆***S*** for threshold ***t_i_*** of ***k***th sampling = P (∆***S_i_ ^k^*** ) = **P*_i_ ^k^* = (# {∆*S_i_ ^j^*: ∆*S_i_ ^j^* ≥ ∆*S_i_ ^k^*} +1) / *J***. (in ***k***+1 column of array B)
7. Interim P-value for tested gene group, taking into account all threshold ***t_i_*** = **P = min{P*_i_*}.** (in first column of array C)
8. Interim P-value for ***m***th sample, taking into account all threshold **t_i_** = **P*^m^* = min {P*_i_^m^*}**. (in ***m***+1 column of array C)
9. Computed P-value = **P_c_ = (# {P*^m^* :P*^m^*≤ P} +1)/*J*** (in array D)

**🡺** 🡺

**Array A** **Array B**

$\begin{matrix} P & P^{1} & P^{1} & ... & P^{J-1} & P^{J} \end{matrix}$ 🡺 $P_{c}$

**Array C** **Array D**

In the event where no sample in ***J*** samplings has more significant value than the tested one, the P-value is conservatively reported as ***J***^-1^ in three associated steps. Hence, computed P-values are significantly biased toward larger values for smaller P-values. In addition, two sampling characteristics build in a highly stringent test, minimizing unwarranted high significance. Firstly, it assigns large P-values to gene groups with similar scores for correlated members (i.e., no evidence of score increment across target propensity thresholds). Secondly, it preserves global score similarities between genes and the auto-correlation observed for a gene, conserving the global properties of the genetic network.

**References**

1. Seguin L, Liot C, Mzali R et al. CUX1 and E2F1 Regulate Coordinated Expression of the Mitotic Complex Genes Ect2, MgcRacGAP, and MKLP1 in S Phase. Mol Cell Biol 2009; 29: 570–581.

2. Reis T, Edgar BA. Negative regulation of dE2F1 by cyclin-dependent kinases controls cell cycle timing. Cell 2004; 117: 253-264.

3. Bracken AP, Ciro M, Cocito A et al. E2F target genes: unraveling the biology. Trends Biochem Sci 2004; 29: 409-417.

4. Dimova DK, Dyson NJ. The E2F transcriptional network: old acquaintances with new faces. Oncogene 2005; 24: 2810–2826.

5. DeGregori J, Johnson DG. Distinct and overlapping roles for E2F family members in transcription, proliferation, and apoptosis. Curr Mol Med 2006; 6: 739–748.

6. Cooper-Kuhn CM, Vroemen M, Brown J et al. Impaired adult neurogenesis in mice lacking the transcription factor E2F1. Mol Cell Neurosci 2002; 21: 312-323.

7. Royzman I, Hayashi-Hagihara A, Dej KJ et al. The E2F cell cycle regulator is required for Drosophila nurse cell DNA replication and apoptosis. Mech Dev 2002; 119: 225-237.

8. Kel AE, Kel-Margoulis OV, Farnham PJ et al. Computer-assisted identification of cell cycle-related genes: new targets for E2F transcription factors. J Mol Biol 2001; 309: 99-120.

9. Bieda M, Xu XQ, Singer MA et al. Unbiased location analysis of E2F1-binding sites suggests a widespread role for E2F1 in the human genome. Genome Res 2006; 16: 595-605.

10. Xu XQ, Bieda M, Jin VX et al. Comprehensive ChIP-chip analysis of E2F1, E2F4, and E2F6 in normal and tumor cells reveals interchangeable roles of E2F family members. Genome Res 2007; 17: 1550-1561.

11. Emili A, Ingles CJ. Promoter-dependent photocrosslinking of the acidic transcriptional activator E2F-1 to the TATA-binding protein. J Biol Chem 1995; 270: 13674–13680.

12. Pearson A, Greenblatt J. Modular organization of the E2F1 activation domain and its interaction with general transcription factors TBP and TFIIH. Oncogene 1997; 15: 2643-2658.

13. Fry CJ, Pearson A, Malinowski E et al. Activation of the murine dihydrofolate reductase promoter by E2F1: A requirement for CBP recruitment. J Biol Chem 1999; 274: 15883–15891.

14. Ross JF, Liu X, Dynlacht BD Mechanism of transcriptional repression of E2F by the retinoblastoma tumor suppressor protein. Mol Cell 1999; 3: 195-205.
