# Supplementary material for: Integrated Transcriptome and Binding Sites Analysis Implicates E2F in the Regulation of Self-Renewal in Human Pluripotent Stem Cells
Source: PLoS One. 2011 Nov 4;6(11):e27231. doi: 10.1371/journal.pone.0027231 (PMC3208628; doi:10.1371/journal.pone.0027231)
Supplement: Data S1 — (PDF) [file pone.0027231.s010.pdf]

Supporting Data S1:

1. FDR analysis, Storey and Tibshirani, 2003 (DE metric)
LEGEND:
DE- differential expression
bp- biological process
pw- pathway
mf- molecular function

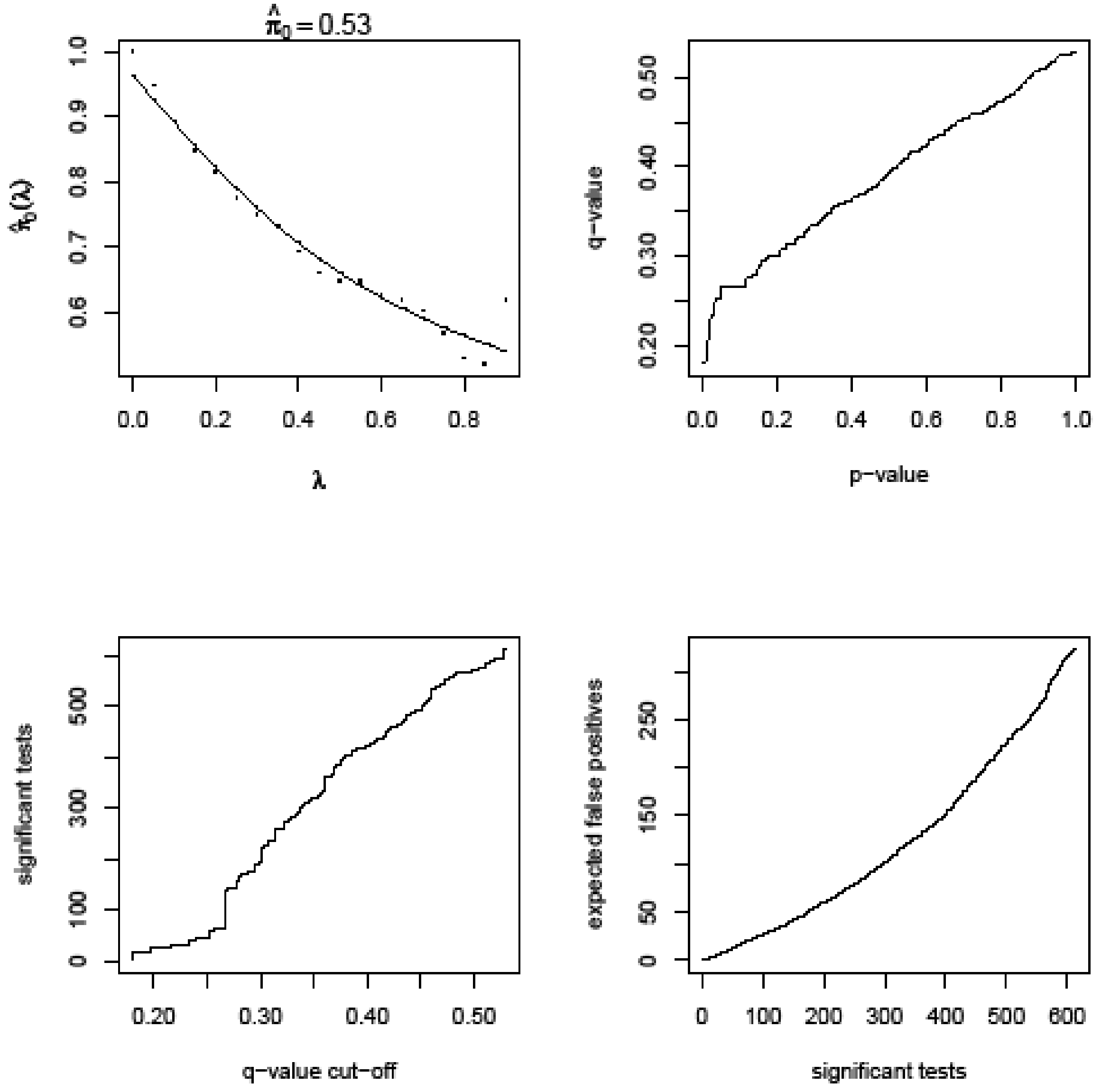

2. Target-cohort analysis P-values
COLUMN LEGEND:
column 1- gene group
column 2- P-value
column 3- gene group type
column 4- q-value

2.1 Differential expression metric

col 1	col 2	col 3	col 4
Vitamin metabolism	0.0015	bp	0.1802574
Cell structure	0.0018	bp	0.1802574
Cell adhesion	0.0022	bp	0.1802574
Transport	0.003	bp	0.1802574
G-protein mediated signaling	0.0039	bp	0.1802574
Neurogenesis	0.0046	bp	0.1802574
Wnt signaling pathway	0.0059	pw	0.1802574
Cell adhesion-mediated signaling	0.0059	bp	0.1802574
Hydroxylase	0.0062	mf	0.1802574
Amino acid transporter	0.0068	mf	0.1802574
Lysosome transport	0.0076	bp	0.1802574
Protease inhibitor	0.0077	mf	0.1802574
G-protein coupled receptor	0.0082	mf	0.1802574
Electron transport	0.0083	bp	0.1802574
Steroid metabolism	0.0085	bp	0.1802574
Oogenesis	0.009	bp	0.1802574
Other lyase	0.0094	mf	0.1802574
Oxygenase	0.0131	mf	0.1976165
Muscle contraction	0.0131	bp	0.1976165
Calcium mediated signaling	0.0134	bp	0.1976165
Amino acid biosynthesis	0.0138	bp	0.1976165
Phosphatase modulator	0.0141	mf	0.1976165
Actin and actin related protein	0.0142	mf	0.1976165
Zinc finger transcription factor	0.0152	mf	0.1976165
Neuronal activities	0.0152	bp	0.1976165
Other protein targeting and localization	0.0174	bp	0.215895
Embryogenesis	0.0181	bp	0.215895
Extracellular matrix glycoprotein	0.0186	mf	0.215895
Other immune and defense	0.0231	bp	0.2333333
Lipid and fatty acid binding	0.024	bp	0.2333333
Extracellular matrix structural protein	0.0245	mf	0.2333333
Peptide hormone	0.0245	mf	0.2333333
Tight junction	0.0251	mf	0.2333333
FGF signaling pathway	0.0264	pw	0.2333333
Amino acid transport	0.0265	bp	0.2333333
Other carbon metabolism	0.0271	bp	0.2333333
Regulation of phosphate metabolism	0.0275	bp	0.2333333
Myelin protein	0.0276	mf	0.2333333
B cell activation	0.028	pw	0.2333333
Interferon receptor	0.0306	mf	0.2393182
EGF receptor signaling pathway	0.0307	pw	0.2393182
Angiogenesis	0.0314	pw	0.2393182

HMG box transcription factor	0.0317	mf	0.2393182
T cell activation	0.0324	pw	0.2393182
JNK cascade	0.0355	bp	0.2524167
mRNA processing factor	0.0358	mf	0.2524167
DNA topoisomerase	0.037	mf	0.2524167
Oxytocin receptor mediated signaling pathway	0.038	pw	0.2524167
Oxidative stress response	0.04	pw	0.2524167
p38 MAPK pathway	0.0407	pw	0.2524167
Steroid hormone-mediated signaling	0.0422	bp	0.2524167
Lipid and fatty acid transport	0.043	bp	0.2524167
Cell motility	0.044	bp	0.2524167
Tubulin	0.0441	mf	0.2524167
Vitamin_cofactor transport	0.0441	bp	0.2524167
De novo pyrmidine ribonucleotides biosythesis	0.0443	pw	0.2524167
Chromatin packaging and remodeling	0.0443	bp	0.2524167
Inflammation mediated by chemokine and cytokine signaling pathway	0.046	pw	0.2524167
Vesicle coat protein	0.0464	mf	0.2524167
Pyruvate metabolism	0.0466	pw	0.2524167
Other receptor	0.0492	mf	0.2621311
Basic helix-loop-helix transcription factor	0.0531	mf	0.2662174
Cytokinesis	0.0536	bp	0.2662174
Hedgehog signaling pathway	0.056	pw	0.2662174
Dehydratase	0.0572	mf	0.2662174
Other transfer_carrier protein	0.058	mf	0.2662174
Huntington disease	0.06	pw	0.2662174
Exocytosis	0.0607	bp	0.2662174
Cadherin signaling pathway	0.0617	pw	0.2662174
Aminobutyrate degradation	0.0618	pw	0.2662174
Carnitine and CoA metabolism	0.0634	pw	0.2662174
Tricarboxylic acid pathway	0.0636	bp	0.2662174
Guanylate cyclase	0.0638	mf	0.2662174
Serine protease inhibitor	0.0644	mf	0.2662174
Actin binding motor protein	0.0645	mf	0.2662174
Carnitine metabolism	0.065	pw	0.2662174
VEGF signaling pathway	0.0657	pw	0.2662174
Other transferase	0.0659	mf	0.2662174
Methylcitrate cycle	0.0681	pw	0.2662174
Peroxidase	0.0689	mf	0.2662174
Ectoderm development	0.069	bp	0.2662174
Actin binding cytoskeletal protein	0.0697	mf	0.2662174
Receptor	0.0702	mf	0.2662174
Extracellular matrix	0.0733	mf	0.2662174
Apoptotic processes	0.0771	bp	0.2662174
MAPKKK cascade	0.0772	bp	0.2662174
DNA helicase	0.0773	mf	0.2662174
Small GTPase	0.0783	mf	0.2662174
Fatty acid metabolism	0.0785	bp	0.2662174
Metabotropic glutamate receptor group I pathway	0.0786	pw	0.2662174
Storage protein	0.0791	mf	0.2662174
Phosphatase inhibitor	0.0798	mf	0.2662174
Growth factor homeostasis	0.0798	bp	0.2662174
Cadherin	0.0806	mf	0.2662174
De novo pyrimidine deoxyribonucleotide biosynthesis	0.0809	pw	0.2662174

General vesicle transport	0.0826	bp	0.2662174
Histamine H1 receptor mediated signaling pathway	0.0828	pw	0.2662174
Transcription factor	0.0834	mf	0.2662174
ATP synthesis	0.0843	pw	0.2662174
Chaperonin	0.0852	mf	0.2662174
Hypoxia response via HIF activation	0.0855	pw	0.2662174
Acetate utilization	0.087	pw	0.2662174
Vision	0.0872	bp	0.2662174
Ascorbate degradation	0.0875	pw	0.2662174
Cysteine protease inhibitor	0.0879	mf	0.2662174
Phospholipase	0.0898	mf	0.2662174
Phosphate metabolism	0.0905	bp	0.2662174
Other miscellaneous function protein	0.0909	mf	0.2662174
Alpha adrenergic receptor signaling pathway	0.0919	pw	0.2662174
Cell adhesion molecule	0.0927	mf	0.2662174
Translation factor	0.0931	mf	0.2662174
Membrane traffic protein	0.0938	mf	0.2662174
DNA replication	0.094	pw	0.2662174
Receptor protein tyrosine kinase signaling pathway	0.094	bp	0.2662174
Primase	0.0942	mf	0.2662174
Proteolysis	0.0951	bp	0.266444
Transfer_carrier protein	0.0965	mf	0.2667708
FAS signaling pathway	0.0972	pw	0.2667708
Induction of apoptosis	0.0981	bp	0.2667708
Angiogenesis	0.0985	bp	0.2667708
PI3 kinase pathway	0.1007	pw	0.2670652
Methyltransferase	0.1019	mf	0.2670652
Other defense and immunity protein	0.1028	mf	0.2670652
Skeletal development	0.1033	bp	0.2670652
Muscle development	0.1047	bp	0.2670652
Nitric oxide biosynthesis	0.1056	bp	0.2670652
Membrane-bound signaling molecule	0.107	mf	0.2670652
Oncogenesis	0.108	bp	0.2670652
mRNA splicing	0.1081	bp	0.2670652
Other blood circulation and gas exchange activity	0.1081	bp	0.2670652
Oxidative phosphorylation	0.1095	bp	0.2670652
Developmental processes	0.1099	bp	0.2670652
Parkinson disease	0.1111	pw	0.2670652
Amino acid catabolism	0.1114	bp	0.2670652
Alzheimer disease-presenilin pathway	0.1118	pw	0.2670652
Phenylethylamine degradation	0.1128	pw	0.2670652
Androgen_estrogene_progesterone biosynthesis	0.1129	pw	0.2670652
Cytokine_chemokine mediated immunity	0.1134	bp	0.2670652
Hydrogen transporter	0.1148	mf	0.2684173
Tumor necrosis factor family member	0.1157	mf	0.2685893
Muscarinic acetylcholine receptor 1 and 3 signaling pathway	0.1176	pw	0.2710638
Calmodulin related protein	0.1206	mf	0.2760211
Damaged DNA-binding protein	0.124	mf	0.2777273
Other carbohydrate metabolism	0.1242	bp	0.2777273
GABA receptor	0.1243	mf	0.2777273
Nerve-nerve synaptic transmission	0.1262	bp	0.2777273
Chromosome segregation	0.1265	bp	0.2777273
Apoptosis signaling pathway	0.1271	pw	0.2777273

DNA replication	0.1283	bp	0.2777273
Phagocytosis	0.1289	bp	0.2777273
Cholesterol metabolism	0.1296	bp	0.2777273
mRNA end-processing and stability	0.1308	bp	0.2777273
Ras Pathway	0.1312	pw	0.2777273
Structural protein	0.1316	mf	0.2777273
Select calcium binding protein	0.1347	mf	0.2793354
Protein targeting	0.1347	bp	0.2793354
Transmembrane receptor regulatory_adaptor protein	0.1355	mf	0.2793354
p53 pathway feedback loops 2	0.1358	pw	0.2793354
Endocytosis	0.1375	bp	0.2793825
Axon guidance mediated by semaphorins	0.1381	pw	0.2793825
Purine metabolism	0.1398	bp	0.2793825
Protein glycosylation	0.1411	bp	0.2793825
Glycosyltransferase	0.1418	mf	0.2793825
Receptor protein serine_threonine kinase signaling pathway	0.142	bp	0.2793825
DNA polymerase processivity factor	0.1422	mf	0.2793825
Cytoskeletal regulation by Rho GTPase	0.1427	pw	0.2793825
Chemokine	0.1436	mf	0.2794611
DNA glycosylase	0.1467	mf	0.2823077
De novo purine biosynthesis	0.1468	pw	0.2823077
Stress response	0.1486	bp	0.282617
Lipid metabolism	0.1487	bp	0.282617
Thyrotropin-releasing hormone receptor signaling pathway	0.1521	pw	0.2870833
Anion transport	0.153	bp	0.2870833
Signal transduction	0.1537	bp	0.2870833
Microtubule binding motor protein	0.1565	mf	0.2906429
Annexin	0.1605	mf	0.295107
Cell surface receptor mediated signal transduction	0.1612	bp	0.295107
5HT2 type receptor mediated signaling pathway	0.162	pw	0.295107
Other intracellular protein traffic	0.1636	bp	0.295107
5-Hydroxytryptamine degredation	0.1664	pw	0.295107
Lyase	0.1668	mf	0.295107
Pyrimidine Metabolism	0.1673	pw	0.295107
Extracellular matrix protein-mediated signaling	0.1674	bp	0.295107
Cell junction protein	0.1675	mf	0.295107
Protein ADP-ribosylation	0.1686	bp	0.295107
Endothelin signaling pathway	0.1694	pw	0.295107
Other cell adhesion molecule	0.1698	mf	0.295107
Other sulfur metabolism	0.171	bp	0.2956117
Proline biosynthesis	0.1738	pw	0.2978026
Insulin_IGF pathway-mitogen activated protein kinase kinase_MAP kinase cascade	0.1741	pw	0.2978026
Asymmetric protein localization	0.1773	bp	0.299201
N-acetylglucosamine metabolism	0.1781	pw	0.299201
Receptor mediated endocytosis	0.1785	bp	0.299201
Signaling molecule	0.1786	mf	0.299201
Coenzyme A biosynthesis	0.1815	pw	0.3012444
p53 pathway	0.1873	pw	0.3012444
CREB transcription factor	0.1891	mf	0.3012444
Natural killer cell mediated immunity	0.1896	bp	0.3012444
Antibacterial response protein	0.1904	mf	0.3012444
Other actin family cytoskeletal protein	0.1904	mf	0.3012444
Single-stranded DNA-binding protein	0.1917	mf	0.3012444

Other homeostasis activities	0.1925 bp	0.3012444
Integrin signalling pathway	0.1929 pw	0.3012444
Other synthase_synthetase	0.1941 mf	0.3012444
Angiotensin II-stimulated signaling through G proteins and beta-arrestin	0.1948 pw	0.3012444
Other pathways of electron transport	0.1966 bp	0.3012444
Pyrimidine metabolism	0.1967 bp	0.3012444
Antioxidation and free radical removal	0.1969 bp	0.3012444
Extracellular matrix linker protein	0.1977 mf	0.3012444
Neurotrophic factor	0.198 mf	0.3012444
Other cell cycle process	0.1994 bp	0.3012444
Other select calcium binding proteins	0.1998 mf	0.3012444
Plasminogen activating cascade	0.2 pw	0.3012444
Ligand-mediated signaling	0.2005 bp	0.3012444
Transferase	0.2014 mf	0.3012444
Xanthine and guanine salvage pathway	0.2018 pw	0.3012444
Oncogene	0.2035 bp	0.3012444
Axon guidance mediated by Slit_Robo	0.2037 pw	0.3012444
Ion channel	0.2042 mf	0.3012444
Nuclear transport	0.2048 bp	0.3012444
DNA-directed DNA polymerase	0.205 mf	0.3012444
Calcium ion homeostasis	0.2058 bp	0.3012444
mRNA transcription elongation	0.2067 bp	0.3012444
Other membrane traffic protein	0.2121 mf	0.3075
Large G-protein	0.2144 mf	0.3075
Intracellular protein traffic	0.215 bp	0.3075
Fertilization	0.2171 bp	0.3075
Extracellular transport and import	0.2179 bp	0.3075
Endoderm development	0.218 bp	0.3075
Alzheimer disease-amyloid secretase pathway	0.2198 pw	0.3075
Defense_immunity protein	0.2207 mf	0.3075
Macrophage-mediated immunity	0.2207 bp	0.3075
Glycosidase	0.2208 mf	0.3075
Other oncogenesis	0.2214 bp	0.3075
Chromatin_chromatin-binding protein	0.2226 mf	0.3078511
Protein targeting and localization	0.2253 bp	0.3102648
Axon guidance mediated by netrin	0.228 pw	0.3126582
Hydrolase	0.2299 mf	0.31278
Other signaling molecule	0.2316 mf	0.31278
Tumor necrosis factor receptor	0.2334 mf	0.31278
Other apoptosis	0.2345 bp	0.31278
P53 pathway feedback loops 1	0.2356 pw	0.31278
Phosphate transport	0.2373 bp	0.31278
Serine protease	0.2377 mf	0.31278
Synthase and synthetase	0.2377 mf	0.31278
G-protein modulator	0.2382 mf	0.31278
Other amino acid metabolism	0.2395 bp	0.31278
Neuromuscular synaptic transmission	0.2397 bp	0.31278
DNA degradation	0.24 bp	0.31278
B-cell- and antibody-mediated immunity	0.2406 bp	0.31278
Glycolysis	0.2416 pw	0.3128287
Other receptor mediated signaling pathway	0.2438 bp	0.3143359
PDGF signaling pathway	0.2454 pw	0.3143359
Exodeoxyribonuclease	0.2468 mf	0.3143359

Ion transport	0.2473	bp	0.3143359
Intracellular signaling cascade	0.2476	bp	0.3143359
Non-motor actin binding protein	0.2516	mf	0.3181712
T-cell mediated immunity	0.2529	bp	0.3185756
mRNA splicing	0.2569	pw	0.3211765
Kinase inhibitor	0.2612	mf	0.3211765
Inhibition of apoptosis	0.2625	bp	0.3211765
mRNA transcription termination	0.2626	bp	0.3211765
Cation transporter	0.2627	mf	0.3211765
Nucleic acid binding	0.2627	mf	0.3211765
Nicotinic acetylcholine receptor signaling pathway	0.264	pw	0.3211765
Heart development	0.2645	bp	0.3211765
Aspartic protease	0.2649	mf	0.3211765
Tyrosine protein kinase receptor	0.2653	mf	0.3211765
Constitutive exocytosis	0.266	bp	0.3211765
Aldolase	0.2678	mf	0.3211765
Endoribonuclease	0.268	mf	0.3211765
Pre-mRNA processing	0.2688	bp	0.3211765
Regulation of vasoconstriction, dilation	0.2705	bp	0.3220238
Other isomerase	0.2731	mf	0.3239325
Lipase	0.2763	mf	0.3265364
Glycolysis	0.2779	bp	0.3272373
Other ligase	0.2822	mf	0.3290337
Kinase activator	0.2839	mf	0.3290337
Cell communication	0.2839	bp	0.3290337
Vitamin biosynthesis	0.285	bp	0.3290337
Meiosis	0.2854	bp	0.3290337
Protein disulfide-isomerase reaction	0.2855	bp	0.3290337
Immunoglobulin receptor family member	0.2913	mf	0.3335526
General transcription by RNA polymerase I	0.2918	pw	0.3335526
Detoxification	0.2925	bp	0.3335526
Transcription regulation by bZIP transcription factor	0.2945	pw	0.3344931
Regulation of carbohydrate metabolism	0.2959	bp	0.3344931
Other oxidoreductase	0.2972	mf	0.3344931
Circadian clock system	0.2975	pw	0.3344931
Other cell junction protein	0.2986	mf	0.3344931
Ribosomal protein	0.2995	mf	0.3344931
Protein phosphatase	0.3021	mf	0.3358333
Protein metabolism and modification	0.3038	bp	0.3358333
Mutase	0.3056	mf	0.3358333
Nucleoside, nucleotide and nucleic acid transport	0.306	bp	0.3358333
Cysteine protease	0.3069	mf	0.3358333
Protein complex assembly	0.3069	bp	0.3358333
Chemosensory perception	0.3107	bp	0.3380428
Synthetase	0.3123	mf	0.3380428
Membrane traffic regulatory protein	0.3141	mf	0.3380428
Ribonucleoprotein	0.3145	mf	0.3380428
Pentose-phosphate shunt	0.3156	bp	0.3380428
Cytokine and chemokine mediated signaling pathway	0.3161	bp	0.3380428
Fatty acid beta-oxidation	0.3162	bp	0.3380428
Other zinc finger transcription factor	0.3193	mf	0.3402377
DNA repair	0.3228	bp	0.3415666
Galactosidase	0.3233	mf	0.3415666

Immunoglobulin	0.3237 mf	0.3415666
Hydratase	0.3278 mf	0.3447735
Other cytokine	0.3293 mf	0.3450399
NF-kappaB cascade	0.3304 bp	0.3450399
Interleukin	0.3316 mf	0.3450399
Protein modification	0.3323 bp	0.3450399
JAK_STAT signaling pathway	0.3355 pw	0.3469375
Carbohydrate kinase	0.337 mf	0.3469375
Carbohydrate metabolism	0.339 bp	0.3469375
Miscellaneous	0.3397 bp	0.3469375
Growth factor	0.3398 mf	0.3469375
Notch signaling pathway	0.3413 pw	0.3469375
Granulocyte-mediated immunity	0.3416 bp	0.3469375
Cell cycle	0.3484 pw	0.3527414
Pterin metabolism	0.3524 bp	0.3556832
DNA-directed RNA polymerase	0.3538 mf	0.3559907
Synthase	0.3561 mf	0.3566
Protease	0.3566 mf	0.3566
Monosaccharide metabolism	0.3577 bp	0.3566028
Nucleotidyltransferase	0.3599 mf	0.3569055
Allantoin degradation	0.3602 pw	0.3569055
Spermatogenesis and motility	0.3645 bp	0.3575
Other cytoskeletal proteins	0.365 mf	0.3575
DNA methyltransferase	0.3652 mf	0.3575
Phospholipid metabolism	0.3656 bp	0.3575
Steroid hormone metabolism	0.3672 bp	0.3575
Other neuronal activity	0.3674 bp	0.3575
Enkephalin release	0.3707 pw	0.3590116
Heterotrimeric G-protein signaling pathway-Gq alpha and Go alpha mediated pathway	0.3719 pw	0.3590116
Coenzyme and prosthetic group metabolism	0.3727 bp	0.3590116
Disaccharide metabolism	0.3737 bp	0.3590116
Amino acid metabolism	0.376 bp	0.3590116
Interferon	0.3763 mf	0.3590116
Other phosphatase	0.3767 mf	0.3590116
Translation elongation factor	0.3785 mf	0.3590116
Mesoderm development	0.379 bp	0.3590116
5-Hydroxytryptamine biosynthesis	0.38 pw	0.3590116
Cortocotropin releasing factor receptor signaling pathway	0.3843 pw	0.3601929
Cholesterol biosynthesis	0.3873 pw	0.3601929
Insulin_IGF pathway-protein kinase B signaling cascade	0.3877 pw	0.3601929
Esterase	0.3879 mf	0.3601929
Hsp 90 family chaperone	0.3879 mf	0.3601929
Olfaction	0.3879 bp	0.3601929
Other nucleoside, nucleotide and nucleic acid metabolism	0.3906 bp	0.3604958
Other developmental process	0.3914 bp	0.3604958
Oxidoreductase	0.3954 mf	0.3604958
Nucleotide phosphatase	0.3955 mf	0.3604958
Determination of dorsal_ventral axis	0.3961 bp	0.3604958
Acetylcholine receptor	0.3965 mf	0.3604958
Other hydrolase	0.3969 mf	0.3604958
Homeostasis	0.3971 bp	0.3604958
Peroxisome transport	0.4012 bp	0.3625556
DNA recombination	0.4016 bp	0.3625556

Other steroid metabolism	0.4081	bp	0.366095
Gametogenesis	0.4087	bp	0.366095
Protein phosphorylation	0.4089	bp	0.366095
Protein biosynthesis	0.4112	bp	0.3671429
Cell structure and motility	0.414	bp	0.3674796
Endodeoxyribonuclease	0.4158	mf	0.3674796
Apolipoprotein	0.416	mf	0.3674796
Regulated exocytosis	0.4161	bp	0.3674796
Adenine and hypoxanthine salvage pathway	0.4183	pw	0.3680712
Interferon-mediated immunity	0.4191	bp	0.3680712
CAM family adhesion molecule	0.4207	mf	0.3680712
Acyltransferase	0.4213	mf	0.3680712
Cell cycle control	0.4254	bp	0.3692717
Transporter	0.4269	mf	0.3692717
Phosphodiesterase	0.4293	mf	0.3692717
Gamma-aminobutyric acid synthesis	0.4293	pw	0.3692717
Voltage-gated ion channel	0.4302	mf	0.3692717
Tyrosine biosynthesis	0.4305	pw	0.3692717
Apoptosis	0.4316	bp	0.3692717
Carbohydrate transport	0.4326	bp	0.3692717
Pheromone response	0.4329	bp	0.3692717
Immunity and defense	0.4371	bp	0.3718783
Protein kinase	0.4391	mf	0.3726044
Intermediate filament	0.4427	mf	0.373961
Other metabolism	0.443	bp	0.373961
Adenylate cyclase	0.4443	mf	0.3740439
Serine_threonine protein kinase receptor	0.4454	mf	0.3740439
TCA cycle	0.4468	pw	0.3742526
Other enzyme activator	0.4496	mf	0.3744962
Phenylalanine biosynthesis	0.4498	pw	0.3744962
Ubiquitin proteasome pathway	0.4517	pw	0.3744962
mRNA transcription	0.4517	bp	0.3744962
Sulfur metabolism	0.453	bp	0.3746183
Interleukin signaling pathway	0.4556	pw	0.3758122
Metabolism of cyclic nucleotides	0.4573	bp	0.3762595
Heterotrimeric G-protein signaling pathway-rod outer segment phototransduction	0.459	pw	0.3764106
Decarboxylase	0.4598	mf	0.3764106
Transaminase	0.4644	mf	0.3783521
Toll receptor signaling pathway	0.4645	pw	0.3783521
GABA-B_receptor_II_signaling	0.4675	pw	0.3795449
Histamine H2 receptor mediated signaling pathway	0.4683	pw	0.3795449
Interleukin receptor	0.4712	mf	0.3809453
Coenzyme metabolism	0.4725	bp	0.3810484
Aminoacyl-tRNA synthetase	0.4787	mf	0.3850928
Fatty acid biosynthesis	0.4821	bp	0.3856985
Sensory perception	0.4826	bp	0.3856985
Endogenous_cannabinoid_signaling	0.4838	pw	0.3856985
Nicotine degradation	0.4842	pw	0.3856985
Synaptic_vesicle_trafficking	0.4861	pw	0.3862653
Ferredoxin metabolism	0.4877	bp	0.3865915
Pentose phosphate pathway	0.4898	pw	0.3873114
Hearing	0.4935	bp	0.38929
Vasopressin synthesis	0.4954	pw	0.3898426

RNA helicase	0.4968	mf	0.39
Deacetylase	0.502	mf	0.3931325
Reverse transcriptase	0.5048	mf	0.394375
Translational regulation	0.5093	bp	0.3969365
Guanyl-nucleotide exchange factor	0.5106	mf	0.3969976
Kinase modulator	0.5167	mf	0.3991647
Centromere DNA-binding protein	0.5175	mf	0.3991647
mRNA capping	0.518	bp	0.3991647
Adrenaline and noradrenaline biosynthesis	0.5183	pw	0.3991647
Surfactant	0.5256	mf	0.4030458
NO mediated signal transduction	0.5265	bp	0.4030458
Other coenzyme and prosthetic group metabolism	0.5278	bp	0.4030458
Helicase	0.5283	mf	0.4030458
Transketolase	0.5362	mf	0.4069697
Epimerase_racemase	0.5365	mf	0.4069697
Synaptic transmission	0.5372	bp	0.4069697
SNARE protein	0.5399	mf	0.408064
Ligase	0.5414	mf	0.4082483
Isomerase	0.5443	mf	0.4084217
Lysine biosynthesis	0.5453	pw	0.4084217
Other microtubule family cytoskeletal protein	0.5454	mf	0.4084217
Metalloprotease inhibitor	0.5568	mf	0.4151373
Gluconeogenesis	0.5577	bp	0.4151373
mRNA polyadenylation	0.5582	bp	0.4151373
Other intracellular signaling cascade	0.5622	bp	0.416637
Cytokine	0.5659	mf	0.416637
MHCI-mediated immunity	0.5678	bp	0.416637
Non-motor microtubule binding protein	0.5689	mf	0.416637
Interferon-gamma signaling pathway	0.5696	pw	0.416637
Phosphorylase	0.5699	mf	0.416637
5HT1 type receptor mediated signaling pathway	0.571	pw	0.416637
JAK-STAT cascade	0.5714	bp	0.416637
Select regulatory molecule	0.5725	mf	0.416637
Protein folding	0.5747	bp	0.416637
mRNA polyadenylation factor	0.5755	mf	0.416637
Cytokine receptor	0.5756	mf	0.416637
Protein-lipid modification	0.5801	bp	0.4184645
Other ligand-gated ion channel	0.5807	mf	0.4184645
G-protein	0.5833	mf	0.4194082
Other transporter	0.5855	mf	0.4200607
Cell cycle	0.5879	bp	0.4208535
Other transport	0.5926	bp	0.4225
Purine metabolism	0.5928	pw	0.4225
Complement-mediated immunity	0.5942	bp	0.4225711
Acyl-CoA metabolism	0.5972	bp	0.4237773
Double-stranded DNA binding protein	0.5986	mf	0.4238453
Major histocompatibility complex antigen	0.6056	mf	0.4278696
Methylmalonyl pathway	0.6091	pw	0.4292532
Complement component	0.6102	mf	0.4292532
Arginine biosynthesis	0.6116	pw	0.4293089
Intermediate filament binding protein	0.619	mf	0.4323611
Cytoskeletal protein	0.62	mf	0.4323611
Formyltetrahydroformate biosynthesis	0.6219	pw	0.4323611

Kinase	0.6224	mf	0.4323611
mRNA splicing factor	0.6226	mf	0.4323611
Cation transport	0.6244	bp	0.4326866
Voltage-gated sodium channel	0.6274	mf	0.4338404
Other enzyme inhibitor	0.6303	mf	0.4349204
2-arachidonoylglycerol biosynthesis	0.6353	pw	0.4360872
Isoleucine biosynthesis	0.6363	pw	0.4360872
Valine biosynthesis	0.6377	pw	0.4360872
Asparagine and aspartate biosynthesis	0.6386	pw	0.4360872
Other protein metabolism	0.6387	bp	0.4360872
Gut mesoderm development	0.6414	bp	0.4364479
Dehydrogenase	0.6435	mf	0.4364479
Heterotrimeric G-protein signaling pathway-Gi alpha and Gs alpha mediated pathway	0.6445	pw	0.4364479
Cyclic nucleotide-gated ion channel	0.6446	mf	0.4364479
Miscellaneous function	0.6503	mf	0.4393919
Salvage pyrimidine deoxyribonucleotides	0.6537	pw	0.4403616
General mRNA transcription activities	0.6553	bp	0.4403616
Mitochondrial transport	0.6558	bp	0.4403616
Nuclear hormone receptor	0.6574	mf	0.4405258
Other extracellular matrix	0.6603	mf	0.4415586
tRNA metabolism	0.6621	bp	0.4418532
Pyridoxal phosphate salvage pathway	0.6675	pw	0.4445441
Phosphatase	0.6706	mf	0.4451837
Other cytokine receptor	0.6712	mf	0.4451837
Blood coagulation	0.6753	pw	0.4469908
TGF-beta receptor	0.6774	mf	0.4474695
Neuropeptide	0.6832	mf	0.4503854
Blood clotting	0.6853	bp	0.4505444
Hsp 70 family chaperone	0.6874	mf	0.4505444
Thiamine metabolism	0.6876	pw	0.4505444
Pyrophosphatase	0.69	mf	0.45058
Gap junction	0.6923	mf	0.45058
Anion channel	0.6926	mf	0.45058
Segment specification	0.6932	bp	0.45058
Neurotransmitter release	0.698	bp	0.4527944
Glycogen metabolism	0.6995	bp	0.4528635
Amino acid activation	0.7063	bp	0.4548725
Glutamate receptor	0.7067	mf	0.4548725
p53 pathway by glucose deprivation	0.7069	pw	0.4548725
Reverse transcription	0.7086	bp	0.4548725
Heme biosynthesis	0.7105	pw	0.4548725
Other nucleic acid binding	0.7113	mf	0.4548725
Other sensory perception	0.7127	bp	0.4548725
Small molecule transport	0.7138	bp	0.4548725
DNA ligase	0.7159	mf	0.455318
Glucosidase	0.7228	mf	0.4587305
Pain sensation	0.7254	bp	0.4587305
Opioid prodynorphin pathway	0.7255	pw	0.4587305
Microtubule family cytoskeletal protein	0.7329	mf	0.4603861
Blood circulation and gas exchange	0.7361	bp	0.4603861
Other RNA-binding protein	0.7379	mf	0.4603861
Mitosis	0.7386	bp	0.4603861
Glutamine glutamate conversion	0.7395	pw	0.4603861

Vitamin B6 metabolism	0.7429	pw	0.4603861
Other polysaccharide metabolism	0.7433	bp	0.4603861
Cell proliferation and differentiation	0.7437	bp	0.4603861
Translation initiation factor	0.7446	mf	0.4603861
Other mRNA transcription	0.7449	bp	0.4603861
Non-vertebrate process	0.7454	bp	0.4603861
Transcription cofactor	0.7493	mf	0.4603861
Amino acid metabolism regulation	0.7505	bp	0.4603861
ATP synthase	0.7514	mf	0.4603861
Carbohydrate transporter	0.7518	mf	0.4603861
MHCII-mediated immunity	0.752	bp	0.4603861
Other protease inhibitor	0.7522	mf	0.4603861
Deaminase	0.7546	mf	0.4609868
Succinate to proprionate conversion	0.7571	pw	0.4613904
Other proteases	0.7581	mf	0.4613904
Voltage-gated potassium channel	0.7645	mf	0.4640951
DNA photolyase	0.7654	mf	0.4640951
Lactation, mammary development	0.7704	bp	0.466257
Metalloprotease	0.7724	mf	0.4665167
Folate biosynthesis	0.7737	pw	0.4665167
RNA catabolism	0.7787	bp	0.4682163
Beta3 adrenergic receptor signaling pathway	0.7794	pw	0.4682163
Sulfur redox metabolism	0.7831	bp	0.469571
ATP-binding cassette ABC transporter	0.7906	mf	0.4722339
Other G-protein modulator	0.791	mf	0.4722339
Polyphosphate biosynthesis	0.7919	bp	0.4722339
Mitochondrial carrier protein	0.7941	mf	0.4726786
Mannose metabolism	0.7971	pw	0.473167
Nuclease	0.798	mf	0.473167
Chaperone	0.8012	mf	0.473167
Acetyltransferase	0.8015	mf	0.473167
Anterior_posterior patterning	0.8022	bp	0.473167
Histone	0.8047	mf	0.4737817
Other chaperones	0.8078	mf	0.4747468
Translation release factor	0.8115	mf	0.4760605
Vitamin D metabolism and pathway	0.8156	pw	0.4776036
Ubiquitin-protein ligase	0.8175	mf	0.4778552
Lipoate_biosynthesis	0.8297	pw	0.4815436
Ionotropic glutamate receptor pathway	0.8312	pw	0.4815436
Leucine biosynthesis	0.832	pw	0.4815436
Action potential propagation	0.8321	bp	0.4815436
DNA metabolism	0.8326	bp	0.4815436
Alanine biosynthesis	0.8327	pw	0.4815436
Carbohydrate phosphatase	0.835	mf	0.482016
Hematopoiesis	0.8437	bp	0.4861746
Other transcription factor	0.8478	mf	0.4876726
Protein methylation	0.8589	bp	0.492888
Other signal transduction	0.8599	bp	0.492888
KRAB box transcription factor	0.872	mf	0.4989437
O-antigen biosynthesis	0.8785	pw	0.5012741
Voltage-gated calcium channel	0.8806	mf	0.5012741
Nitrogen metabolism	0.8807	bp	0.5012741
Muscarinic acetylcholine receptor 2 and 4 signaling pathway	0.8847	pw	0.5026705

Fructose galactose metabolism	0.8938	pw	0.5069546
Pinocytosis	0.8977	bp	0.5078689
Dopamine receptor mediated signaling pathway	0.8997	pw	0.5078689
mRNA transcription initiation	0.9001	bp	0.5078689
Tumor suppressor	0.9067	bp	0.5094473
Replication origin binding protein	0.9069	mf	0.5094473
Other ion channel	0.9076	mf	0.5094473
RNA localization	0.9098	bp	0.5098017
RNA methyltransferase	0.9129	mf	0.5099485
Nucleotide kinase	0.9132	mf	0.5099485
Basal transcription factor	0.916	mf	0.5103168
Other kinase	0.917	mf	0.5103168
Homeobox transcription factor	0.9252	mf	0.5131783
Sex determination	0.9253	bp	0.5131783
Porphyrin metabolism	0.9299	bp	0.5148509
Non-receptor tyrosine protein kinase	0.9373	mf	0.5180655
Salvage pyrimidine ribonucleotides	0.9412	pw	0.5189552
Oxidase	0.9429	mf	0.5189552
Reductase	0.9437	mf	0.5189552
Cyclase	0.9597	mf	0.5268623
General transcription regulation	0.9619	pw	0.5269376
rRNA metabolism	0.9635	bp	0.5269376
Protein acetylation	0.9654	bp	0.5269376
Glucose homeostasis	0.9689	bp	0.5269376
Exoribonuclease	0.971	mf	0.5269376
5HT3 type receptor mediated signaling pathway	0.9712	pw	0.5269376
Beta1 adrenergic receptor signaling pathway	0.9732	pw	0.5269376
Fatty acid desaturation	0.9733	bp	0.5269376
Non-receptor serine_threonine protein kinase	0.9777	mf	0.5269376
Metabotropic glutamate receptor group III pathway	0.9788	pw	0.5269376
Serine glycine biosynthesis	0.9819	pw	0.5269376
5HT4 type receptor mediated signaling pathway	0.9832	pw	0.5269376
Beta2 adrenergic receptor signaling pathway	0.9835	pw	0.5269376
Metabotropic glutamate receptor group II pathway	0.9837	pw	0.5269376
Other enzyme regulator	0.9852	mf	0.5269376
Amylase	0.9868	mf	0.5269376
Opioid proenkephalin pathway	0.9874	pw	0.5269376
TGF-beta signaling pathway	0.9909	pw	0.5274877
Opioid proopiomelanocortin pathway	0.9928	pw	0.5274877
Taste	0.9933	bp	0.5274877
Other DNA-binding protein	0.9979	mf	0.5290661

2.2 DE similarity (covariance)

col 1	col 2	col 3	col 4
Hydroxylase	0.0007	mf	0.2344891
Vitamin metabolism	0.0019	bp	0.2344891
Other protein targeting and localization	0.0021	bp	0.2344891
Protease inhibitor	0.0023	mf	0.2344891
EGF receptor signaling pathway	0.0024	pw	0.2344891
FGF signaling pathway	0.003	pw	0.2344891
Lysosome transport	0.0041	bp	0.2344891
Other carbon metabolism	0.005	bp	0.2344891
Oogenesis	0.0077	bp	0.2344891

Muscle contraction	0.0079	bp	0.2344891
Actin and actin related protein	0.008	mf	0.2344891
Steroid metabolism	0.0082	bp	0.2344891
p38 MAPK pathway	0.0096	pw	0.2344891
G-protein mediated signaling	0.01	bp	0.2344891
Phosphatase modulator	0.0101	mf	0.2344891
PI3 kinase pathway	0.0104	pw	0.2344891
Oxidative stress response	0.0109	pw	0.2344891
Parkinson disease	0.0117	pw	0.2344891
Transport	0.0118	bp	0.2344891
Protein glycosylation	0.0139	bp	0.2344891
JNK cascade	0.0147	bp	0.2344891
Phenylethylamine degradation	0.0149	pw	0.2344891
p53 pathway	0.0152	pw	0.2344891
Calcium mediated signaling	0.0155	bp	0.2344891
Interferon receptor	0.0158	mf	0.2344891
Tight junction	0.0162	mf	0.2344891
Basic helix-loop-helix transcription factor	0.0163	mf	0.2344891
Amino acid transporter	0.017	mf	0.2344891
Electron transport	0.017	bp	0.2344891
Aminobutyrate degradation	0.0183	pw	0.244003
Tubulin	0.0215	mf	0.2628573
Lipid and fatty acid binding	0.0215	bp	0.2628573
B cell activation	0.0222	pw	0.2628573
Pyruvate metabolism	0.0225	pw	0.2628573
Cell adhesion	0.023	bp	0.2628573
Vesicle coat protein	0.0239	mf	0.2655557
Other transferase	0.0247	mf	0.2670271
Extracellular matrix glycoprotein	0.0266	mf	0.2678261
Wnt signaling pathway	0.027	pw	0.2678261
Peroxidase	0.0286	mf	0.2678261
DNA topoisomerase	0.0302	mf	0.2678261
Dehydratase	0.0314	mf	0.2678261
T cell activation	0.0318	pw	0.2678261
Neurogenesis	0.0322	bp	0.2678261
5-Hydroxytryptamine degredation	0.0339	pw	0.2678261
MAPKKK cascade	0.0341	bp	0.2678261
Neuronal activities	0.0343	bp	0.2678261
Other cell cycle process	0.0344	bp	0.2678261
Myelin protein	0.0345	mf	0.2678261
Oxytocin receptor mediated signaling pathway	0.0345	pw	0.2678261
Muscle development	0.0353	bp	0.2678261
mRNA processing factor	0.0362	mf	0.2678261
Tricarboxylic acid pathway	0.0369	bp	0.2678261
Receptor	0.0373	mf	0.2678261
Vitamin_cofactor transport	0.0381	bp	0.2678261
Cysteine protease inhibitor	0.04	mf	0.2678261
Cell cycle	0.0409	bp	0.2678261
Other homeostasis activities	0.0414	bp	0.2678261
Storage protein	0.0415	mf	0.2678261
Nucleoside, nucleotide and nucleic acid transport	0.0422	bp	0.2678261
Transcription factor	0.0425	mf	0.2678261
G-protein coupled receptor	0.0431	mf	0.2678261

Steroid hormone-mediated signaling	0.0431 bp	0.2678261
Oxygenase	0.0435 mf	0.2678261
Peptide hormone	0.0438 mf	0.2678261
Huntington disease	0.0444 pw	0.2678261
Guanylate cyclase	0.0457 mf	0.2678261
Other amino acid metabolism	0.046 bp	0.2678261
Translation factor	0.0462 mf	0.2678261
Extracellular matrix	0.0484 mf	0.2738028
Ascorbate degradation	0.0486 pw	0.2738028
Axon guidance mediated by netrin	0.0514 pw	0.2855556
Regulated exocytosis	0.0533 bp	0.2920548
Phosphatase inhibitor	0.0568 mf	0.3033766
Cell structure	0.0575 bp	0.3033766
Lipid and fatty acid transport	0.0582 bp	0.3033766
Cholesterol metabolism	0.0584 bp	0.3033766
mRNA splicing	0.0606 bp	0.3093671
Axon guidance mediated by Slit_Robo	0.0611 pw	0.3093671
Transfer_carrier protein	0.064 mf	0.3172277
Cell adhesion molecule	0.0645 mf	0.3172277
Ectoderm development	0.0651 bp	0.3172277
Chaperonin	0.0662 mf	0.3172277
Damaged DNA-binding protein	0.0668 mf	0.3172277
Primase	0.0688 mf	0.3172277
Protein phosphatase	0.0688 mf	0.3172277
Zinc finger transcription factor	0.0701 mf	0.3172277
Hydrogen transporter	0.0708 mf	0.3172277
DNA helicase	0.0714 mf	0.3172277
Other transfer_carrier protein	0.0714 mf	0.3172277
Embryogenesis	0.0737 bp	0.3172277
Cell communication	0.074 bp	0.3172277
Other carbohydrate metabolism	0.0745 bp	0.3172277
Cytokine_chemokine mediated immunity	0.0753 bp	0.3172277
Phagocytosis	0.0773 bp	0.3172277
Hedgehog signaling pathway	0.0774 pw	0.3172277
Macrophage-mediated immunity	0.0791 bp	0.3172277
DNA replication	0.0793 pw	0.3172277
Oncogene	0.0794 bp	0.3172277
Inflammation mediated by chemokine and cytokine signaling pathway	0.0796 pw	0.3172277
p53 pathway feedback loops 2	0.0801 pw	0.3172277
De novo pyrmidine ribonucleotides biosythesis	0.081 pw	0.3176471
Other receptor	0.0823 mf	0.3196117
DNA glycosylase	0.0841 mf	0.3234615
Serine protease inhibitor	0.0862 mf	0.3264151
FAS signaling pathway	0.0865 pw	0.3264151
Protein modification	0.0874 bp	0.326729
Extracellular matrix protein-mediated signaling	0.0889 bp	0.3290909
Carbohydrate transport	0.0905 bp	0.3290909
Intracellular signaling cascade	0.0905 bp	0.3290909
Endocytosis	0.0934 bp	0.3346429
Other membrane traffic protein	0.0937 mf	0.3346429
Hearing	0.0962 bp	0.3356522
Cholesterol biosynthesis	0.0964 pw	0.3356522
Oxidative phosphorylation	0.0965 bp	0.3356522

Insulin_IGF pathway-mitogen activated protein kinase kinase_MAP kinase cascade	0.0981 pw	0.3364103
DNA replication	0.0984 bp	0.3364103
Calmodulin related protein	0.0995 mf	0.3372881
DNA polymerase processivity factor	0.1004 mf	0.337479
Kinase inhibitor	0.1039 mf	0.3418182
Select calcium binding protein	0.1045 mf	0.3418182
Mesoderm development	0.1054 bp	0.3418182
Other metabolism	0.1064 bp	0.3418182
Axon guidance mediated by semaphorins	0.11 pw	0.3418182
Cell cycle	0.1101 pw	0.3418182
Other immune and defense	0.1114 bp	0.3418182
Ras Pathway	0.1114 pw	0.3418182
Notch signaling pathway	0.112 pw	0.3418182
Structural protein	0.1123 mf	0.3418182
Phosphate metabolism	0.1134 bp	0.3418182
Calcium ion homeostasis	0.1136 bp	0.3418182
Cadherin	0.1159 mf	0.3418182
Amino acid biosynthesis	0.1162 bp	0.3418182
Other sulfur metabolism	0.1162 bp	0.3418182
Protein-lipid modification	0.1171 bp	0.3418182
Cell adhesion-mediated signaling	0.1179 bp	0.3418182
Amino acid catabolism	0.1193 bp	0.3418182
Chromatin packaging and remodeling	0.1196 bp	0.3418182
Integrin signalling pathway	0.1196 pw	0.3418182
Vision	0.1203 bp	0.3418182
Histamine H1 receptor mediated signaling pathway	0.1214 pw	0.3418182
Signal transduction	0.1215 bp	0.3418182
Androgen_estrogene_progesterone biosynthesis	0.1222 pw	0.3418182
Membrane traffic protein	0.1252 mf	0.3477778
Nucleic acid binding	0.1263 mf	0.3484138
Cytoskeletal regulation by Rho GTPase	0.1276 pw	0.3484393
Defense_immunity protein	0.1282 mf	0.3484393
Other defense and immunity protein	0.1316 mf	0.3484393
Other cytokine	0.1317 mf	0.3484393
Glycolysis	0.1325 bp	0.3484393
Other cell adhesion molecule	0.1329 mf	0.3484393
Pre-mRNA processing	0.1338 bp	0.3484393
Other signaling molecule	0.1356 mf	0.3484393
Other actin family cytoskeletal protein	0.1387 mf	0.3484393
Phosphate transport	0.139 bp	0.3484393
Sulfur metabolism	0.1413 bp	0.3484393
Metabotropic glutamate receptor group I pathway	0.142 pw	0.3484393
Signaling molecule	0.1421 mf	0.3484393
Phospholipid metabolism	0.1422 bp	0.3484393
Cytokinesis	0.1425 bp	0.3484393
Extracellular matrix linker protein	0.1435 mf	0.3484393
Cadherin signaling pathway	0.1441 pw	0.3484393
De novo pyrimidine deoxyribonucleotide biosynthesis	0.1442 pw	0.3484393
Other ligase	0.1453 mf	0.3484393
DNA-directed DNA polymerase	0.1459 mf	0.3484393
Adenine and hypoxanthine salvage pathway	0.1463 pw	0.3484393
Protein targeting	0.1464 bp	0.3484393
Gamma-aminobutyric acid synthesis	0.1483 pw	0.3484393

Apoptosis	0.149	bp	0.3484393
Endoribonuclease	0.1498	mf	0.3484393
Transmembrane receptor regulatory_adaptor protein	0.1502	mf	0.3484393
Protein phosphorylation	0.1502	bp	0.3484393
Serine protease	0.1507	mf	0.3484393
Cortocotropin releasing factor receptor signaling pathway	0.1531	pw	0.351954
Ion channel	0.1541	mf	0.3522286
5HT2 type receptor mediated signaling pathway	0.1578	pw	0.3524324
CREB transcription factor	0.1583	mf	0.3524324
Ion transport	0.1586	bp	0.3524324
Extracellular transport and import	0.1597	bp	0.3524324
Tumor necrosis factor receptor	0.1601	mf	0.3524324
Lipid metabolism	0.1611	bp	0.3524324
Thyrotropin-releasing hormone receptor signaling pathway	0.1619	pw	0.3524324
Cell cycle control	0.162	bp	0.3524324
Skeletal development	0.163	bp	0.3524324
Apoptosis signaling pathway	0.163	pw	0.3524324
Regulation of vasoconstriction, dilation	0.1639	bp	0.3524731
Ribosomal protein	0.166	mf	0.3550802
Hypoxia response via HIF activation	0.1707	pw	0.3574634
Meiosis	0.1708	bp	0.3574634
Other phosphatase	0.1722	mf	0.3574634
Carbohydrate metabolism	0.1722	bp	0.3574634
mRNA polyadenylation factor	0.173	mf	0.3574634
Amino acid transport	0.1737	bp	0.3574634
Insulin_IGF pathway-protein kinase B signaling cascade	0.1745	pw	0.3574634
TCA cycle	0.1747	pw	0.3574634
Phospholipase	0.1758	mf	0.3574634
Transferase	0.1767	mf	0.3574634
DNA repair	0.1771	bp	0.3574634
Muscarinic acetylcholine receptor 1 and 3 signaling pathway	0.1795	pw	0.3574634
Membrane-bound signaling molecule	0.1803	mf	0.3574634
Allantoin degradation	0.182	pw	0.3574634
Amino acid metabolism	0.1823	bp	0.3574634
Cell motility	0.1825	bp	0.3574634
VEGF signaling pathway	0.1829	pw	0.3574634
Alpha adrenergic receptor signaling pathway	0.1832	pw	0.3574634
Kinase activator	0.1855	mf	0.3584541
Cytokine and chemokine mediated signaling pathway	0.1855	bp	0.3584541
Cell surface receptor mediated signal transduction	0.1877	bp	0.3609615
Other isomerase	0.1894	mf	0.362488
Apoptotic processes	0.1939	bp	0.36625
Chemokine	0.1955	mf	0.36625
Disaccharide metabolism	0.1958	bp	0.36625
Exodeoxyribonuclease	0.1981	mf	0.36625
Voltage-gated ion channel	0.1983	mf	0.36625
Synthase and synthetase	0.1988	mf	0.36625
General transcription by RNA polymerase I	0.1996	pw	0.36625
Hydrolase	0.2002	mf	0.36625
Protein biosynthesis	0.2007	bp	0.36625
Transcription regulation by bZIP transcription factor	0.2027	pw	0.36625
Immunity and defense	0.2035	bp	0.36625
HMG box transcription factor	0.2039	mf	0.36625

Angiogenesis	0.2041 pw	0.36625
Proteolysis	0.2047 bp	0.36625
Immunoglobulin receptor family member	0.2051 mf	0.36625
Actin binding motor protein	0.2072 mf	0.3664629
Fatty acid metabolism	0.2074 bp	0.3664629
Glycosidase	0.2093 mf	0.3664629
Pentose-phosphate shunt	0.2097 bp	0.3664629
Induction of apoptosis	0.2098 bp	0.3664629
Stress response	0.214 bp	0.3721739
GABA receptor	0.2216 mf	0.3826609
P53 pathway feedback loops 1	0.2222 pw	0.3826609
Proline biosynthesis	0.2229 pw	0.3826609
Nucleotidyltransferase	0.224 mf	0.382906
Endothelin signaling pathway	0.2258 pw	0.3838983
Circadian clock system	0.2265 pw	0.3838983
Serine_threonine protein kinase receptor	0.2303 mf	0.3877311
Plasminogen activating cascade	0.2307 pw	0.3877311
Chromosome segregation	0.2318 bp	0.3879498
Glycolysis	0.2338 pw	0.3879835
Anion transport	0.2348 bp	0.3879835
Angiotensin II-stimulated signaling through G proteins and beta-arrestin	0.2356 pw	0.3879835
Extracellular matrix structural protein	0.2357 mf	0.3879835
Spermatogenesis and motility	0.2381 bp	0.3880162
Alzheimer disease-presenilin pathway	0.2384 pw	0.3880162
Other miscellaneous function protein	0.2395 mf	0.3880162
Coenzyme and prosthetic group metabolism	0.2396 bp	0.3880162
Other receptor mediated signaling pathway	0.2454 bp	0.3956226
Ligand-mediated signaling	0.2474 bp	0.3956226
Mitochondrial transport	0.2482 bp	0.3956226
Protein disulfide-isomerase reaction	0.2495 bp	0.3956226
Antioxidation and free radical removal	0.2501 bp	0.3956226
Protein metabolism and modification	0.2513 bp	0.3956226
Synthase	0.2525 mf	0.3956226
Cation transporter	0.2538 mf	0.3956226
Growth factor	0.2548 mf	0.3956226
Decarboxylase	0.2566 mf	0.3956226
Aminoacyl-tRNA synthetase	0.2575 mf	0.3956226
Natural killer cell mediated immunity	0.2578 bp	0.3956226
Protein complex assembly	0.2583 bp	0.3956226
Lysine biosynthesis	0.259 pw	0.3956226
Heterotrimeric G-protein signaling pathway-Gq alpha and Go alpha mediated pathway	0.2597 pw	0.3956226
Cell proliferation and differentiation	0.2618 bp	0.3956226
Lyase	0.262 mf	0.3956226
mRNA polyadenylation	0.2621 bp	0.3956226
Cysteine protease	0.266 mf	0.3991045
mRNA end-processing and stability	0.2668 bp	0.3991045
Aldolase	0.2674 mf	0.3991045
DNA recombination	0.2704 bp	0.4020818
Anterior_posterior patterning	0.2723 bp	0.4034074
Oncogenesis	0.2758 bp	0.4070849
Actin binding cytoskeletal protein	0.2774 mf	0.4079412
Other synthase_synthetase	0.2873 mf	0.4169444
Other lyase	0.2893 mf	0.4169444

Methylmalonyl pathway	0.2901	pw	0.4169444
Mitosis	0.2903	bp	0.4169444
CAM family adhesion molecule	0.2919	mf	0.4169444
Fatty acid beta-oxidation	0.2937	bp	0.4169444
General vesicle transport	0.2937	bp	0.4169444
Glycosyltransferase	0.2944	mf	0.4169444
Tyrosine biosynthesis	0.2946	pw	0.4169444
G-protein modulator	0.2955	mf	0.4169444
Phenylalanine biosynthesis	0.2964	pw	0.4169444
Chromatin_chromatin-binding protein	0.2979	mf	0.4169444
Other cell junction protein	0.2986	mf	0.4169444
Other transporter	0.299	mf	0.4169444
Developmental processes	0.2994	bp	0.4169444
DNA-directed RNA polymerase	0.3002	mf	0.4169444
Heart development	0.3025	bp	0.4186851
Receptor protein tyrosine kinase signaling pathway	0.3098	bp	0.4273103
Intracellular protein traffic	0.3124	bp	0.4288435
Interleukin	0.314	mf	0.4288435
Protein targeting and localization	0.3144	bp	0.4288435
Vasopressin synthesis	0.3152	pw	0.4288435
Cell structure and motility	0.3181	bp	0.43
Vitamin biosynthesis	0.3182	bp	0.43
Microtubule binding motor protein	0.3215	mf	0.4318121
Asparagine and aspartate biosynthesis	0.3217	pw	0.4318121
Lipase	0.3258	mf	0.4340199
Endoderm development	0.3258	bp	0.4340199
Protease	0.3266	mf	0.4340199
Other coenzyme and prosthetic group metabolism	0.3278	bp	0.4341722
Lipoate_biosynthesis	0.3316	pw	0.4377558
Other microtubule family cytoskeletal protein	0.3333	mf	0.4385526
Other nucleoside, nucleotide and nucleic acid metabolism	0.3365	bp	0.4394788
Chemosensory perception	0.3367	bp	0.4394788
Arginine biosynthesis	0.3373	pw	0.4394788
Other enzyme activator	0.3409	mf	0.4423077
Pyrimidine metabolism	0.3418	bp	0.4423077
Endogenous_cannabinoid_signaling	0.3441	pw	0.4423077
Protein folding	0.3445	bp	0.4423077
Kinase modulator	0.345	mf	0.4423077
Pinocytosis	0.3511	bp	0.4457321
Nicotinic acetylcholine receptor signaling pathway	0.3542	pw	0.4457321
Neurotrophic factor	0.3543	mf	0.4457321
Other select calcium binding proteins	0.3546	mf	0.4457321
Coenzyme A biosynthesis	0.3552	pw	0.4457321
Exocytosis	0.3566	bp	0.4457321
Nerve-nerve synaptic transmission	0.3568	bp	0.4457321
Interferon	0.3577	mf	0.4457321
Gluconeogenesis	0.3577	bp	0.4457321
Interleukin signaling pathway	0.363	pw	0.4509317
Helicase	0.3647	mf	0.4516409
Other apoptosis	0.3682	bp	0.4530061
Other pathways of electron transport	0.3687	bp	0.4530061
Protein kinase	0.3692	mf	0.4530061
mRNA transcription termination	0.3715	bp	0.453617

Succinate to proprionate conversion	0.3721	pw	0.453617
Non-motor actin binding protein	0.3731	mf	0.453617
DNA ligase	0.3799	mf	0.4604848
Apolipoprotein	0.3823	mf	0.461994
Complement-mediated immunity	0.3839	bp	0.4625301
Anion channel	0.3877	mf	0.4643114
Pyridoxal phosphate salvage pathway	0.3877	pw	0.4643114
Formyltetrahydroformate biosynthesis	0.3894	pw	0.4649552
Acetylcholine receptor	0.3918	mf	0.466393
Other polysaccharide metabolism	0.3954	bp	0.466393
Pterin metabolism	0.3968	bp	0.466393
Cytoskeletal protein	0.3969	mf	0.466393
Deacetylase	0.3971	mf	0.466393
Double-stranded DNA binding protein	0.3976	mf	0.466393
Select regulatory molecule	0.3992	mf	0.4669006
Muscarinic acetylcholine receptor 2 and 4 signaling pathway	0.4011	pw	0.4677551
Growth factor homeostasis	0.4051	bp	0.4703768
Phosphodiesterase	0.4057	mf	0.4703768
Alzheimer disease-amyloid secretase pathway	0.4085	pw	0.4710482
Adenylate cyclase	0.4101	mf	0.4710482
Transporter	0.411	mf	0.4710482
Asymmetric protein localization	0.4132	bp	0.4710482
Nuclear hormone receptor	0.4133	mf	0.4710482
Acyl-CoA metabolism	0.4141	bp	0.4710482
Pain sensation	0.4155	bp	0.4710482
Miscellaneous	0.4157	bp	0.4710482
Large G-protein	0.417	mf	0.4711864
MHCII-mediated immunity	0.4191	bp	0.4722254
tRNA metabolism	0.4238	bp	0.4761003
B-cell- and antibody-mediated immunity	0.4265	bp	0.4761003
Pyrophosphatase	0.4269	mf	0.4761003
Isomerase	0.4273	mf	0.4761003
Other RNA-binding protein	0.4299	mf	0.4764543
Kinase	0.43	mf	0.4764543
Thiamine metabolism	0.435	pw	0.4793583
Tyrosine protein kinase receptor	0.4355	mf	0.4793583
Phosphorylase	0.4384	mf	0.4793583
DNA methyltransferase	0.4399	mf	0.4793583
PDGF signaling pathway	0.4423	pw	0.4793583
Endodeoxyribonuclease	0.4424	mf	0.4793583
Angiogenesis	0.4431	bp	0.4793583
Translation elongation factor	0.4442	mf	0.4793583
Other intracellular protein traffic	0.4454	bp	0.4793583
Interferon-mediated immunity	0.4474	bp	0.4793583
Amino acid activation	0.4478	bp	0.4793583
Reverse transcriptase	0.4479	mf	0.4793583
Interleukin receptor	0.4482	mf	0.4793583
Toll receptor signaling pathway	0.4494	pw	0.47936
Annexin	0.4513	mf	0.4801064
Surfactant	0.4551	mf	0.4828647
Nucleotide phosphatase	0.4571	mf	0.4837037
Valine biosynthesis	0.4637	pw	0.4878351
Other cytokine receptor	0.4659	mf	0.4878351

Intermediate filament binding protein	0.4665 mf	0.4878351
T-cell mediated immunity	0.4681 bp	0.4878351
Other ligand-gated ion channel	0.4695 mf	0.4878351
Translational regulation	0.4704 bp	0.4878351
Small GTPase	0.4714 mf	0.4878351
mRNA capping	0.4717 bp	0.4878351
Isoleucine biosynthesis	0.4722 pw	0.4878351
Mutase	0.4732 mf	0.4878351
Vitamin D metabolism and pathway	0.4762 pw	0.4896658
mRNA transcription elongation	0.48 bp	0.4923077
Action potential propagation	0.4823 bp	0.4926209
Other oncogenesis	0.4831 bp	0.4926209
Other hydrolase	0.484 mf	0.4926209
Synaptic_vesicle_trafficking	0.4866 pw	0.4930633
Other cytoskeletal proteins	0.4869 mf	0.4930633
Sensory perception	0.4913 bp	0.4950126
Ubiquitin proteasome pathway	0.4913 pw	0.4950126
Intermediate filament	0.4983 mf	0.5002494
Cytokine	0.5004 mf	0.5002494
ATP synthesis	0.5004 pw	0.5002494
Glutamine glutamate conversion	0.5015 pw	0.5002494
General mRNA transcription activities	0.5028 bp	0.5002985
Histamine H2 receptor mediated signaling pathway	0.5072 pw	0.5034243
Granulocyte-mediated immunity	0.5111 bp	0.5060396
Regulation of phosphate metabolism	0.5187 bp	0.5113171
Interferon-gamma signaling pathway	0.5207 pw	0.5113171
Other developmental process	0.5226 bp	0.5113171
Centromere DNA-binding protein	0.5228 mf	0.5113171
Gut mesoderm development	0.5241 bp	0.5113171
Other intracellular signaling cascade	0.5241 bp	0.5113171
Dopamine receptor mediated signaling pathway	0.5258 pw	0.5117275
Single-stranded DNA-binding protein	0.5281 mf	0.5127184
Nuclease	0.5324 mf	0.5128741
Tumor suppressor	0.5329 bp	0.5128741
Other oxidoreductase	0.5348 mf	0.5128741
Other enzyme inhibitor	0.5352 mf	0.5128741
Gametogenesis	0.5364 bp	0.5128741
Neurotransmitter release	0.5368 bp	0.5128741
Steroid hormone metabolism	0.5388 bp	0.5128741
Enkephalin release	0.539 pw	0.5128741
Vitamin B6 metabolism	0.5398 pw	0.5128741
Ligase	0.5451 mf	0.5166825
Pheromone response	0.5484 bp	0.5174528
Beta3 adrenergic receptor signaling pathway	0.5485 pw	0.5174528
Voltage-gated sodium channel	0.5542 mf	0.5201874
Segment specification	0.5547 bp	0.5201874
Translation release factor	0.5553 mf	0.5201874
Beta2 adrenergic receptor signaling pathway	0.5609 pw	0.5225806
Fertilization	0.5615 bp	0.5225806
JAK-STAT cascade	0.5624 bp	0.5225806
Beta1 adrenergic receptor signaling pathway	0.5661 pw	0.5225806
Aspartic protease	0.5666 mf	0.5225806
Hematopoiesis	0.5667 bp	0.5225806

Synaptic transmission	0.567 bp	0.5225806
Ribonucleoprotein	0.5722 mf	0.5248182
5HT4 type receptor mediated signaling pathway	0.5756 pw	0.5248182
Purine metabolism	0.5758 bp	0.5248182
O-antigen biosynthesis	0.5758 pw	0.5248182
Heme biosynthesis	0.5767 pw	0.5248182
Methylcitrate cycle	0.5773 pw	0.5248182
RNA helicase	0.58 mf	0.5260771
Antibacterial response protein	0.5824 mf	0.5270588
Detoxification	0.5875 bp	0.5295495
Other proteases	0.5878 mf	0.5295495
Hsp 70 family chaperone	0.6019 mf	0.5383007
De novo purine biosynthesis	0.6032 pw	0.5383007
Acetyltransferase	0.604 mf	0.5383007
MHCI-mediated immunity	0.606 bp	0.5383007
Cyclic nucleotide-gated ion channel	0.6084 mf	0.5383007
Immunoglobulin	0.609 mf	0.5383007
Major histocompatibility complex antigen	0.6091 mf	0.5383007
Determination of dorsal_ventral axis	0.6109 bp	0.5383007
mRNA transcription	0.6118 bp	0.5383007
Neuromuscular synaptic transmission	0.6132 bp	0.5383007
Receptor protein serine_threonine kinase signaling pathway	0.6145 bp	0.5383007
Nitrogen metabolism	0.6149 bp	0.5383007
Tumor necrosis factor family member	0.616 mf	0.5383007
Other nucleic acid binding	0.6168 mf	0.5383007
Metabolism of cyclic nucleotides	0.6177 bp	0.5383007
RNA catabolism	0.6239 bp	0.5425217
Synthetase	0.6283 mf	0.5451627
Homeostasis	0.637 bp	0.5515152
Other blood circulation and gas exchange activity	0.6409 bp	0.552618
Peroxisome transport	0.6411 bp	0.552618
Transaminase	0.6438 mf	0.552618
p53 pathway by glucose deprivation	0.6438 pw	0.552618
Cell junction protein	0.6463 mf	0.553576
Replication origin binding protein	0.6529 mf	0.5580342
Other transport	0.6547 bp	0.5583795
ATP synthase	0.6587 mf	0.5605957
Chaperone	0.6731 mf	0.56827
Deaminase	0.6731 mf	0.56827
Histone	0.6734 mf	0.56827
Nucleotide kinase	0.6734 mf	0.56827
Translation initiation factor	0.6758 mf	0.5684034
Porphyrin metabolism	0.6764 bp	0.5684034
Non-receptor serine_threonine protein kinase	0.6792 mf	0.5693724
NF-kappaB cascade	0.6804 bp	0.5693724
Xanthine and guanine salvage pathway	0.6823 pw	0.5697704
Inhibition of apoptosis	0.687 bp	0.5718919
Pyrimidine Metabolism	0.6877 pw	0.5718919
Glucosidase	0.6903 mf	0.5728631
5-Hydroxytryptamine biosynthesis	0.6928 pw	0.5733333
Reductase	0.6948 mf	0.5733333
Olfaction	0.6961 bp	0.5733333
Ferredoxin metabolism	0.6966 bp	0.5733333

Acyltransferase	0.7005 mf	0.574664
Other signal transduction	0.7021 bp	0.574664
Other protein metabolism	0.7031 bp	0.574664
Fatty acid biosynthesis	0.7042 bp	0.574664
GABA-B_receptor_II_signaling	0.7054 pw	0.574664
Opioid prodynorphin pathway	0.7094 pw	0.5749194
Membrane traffic regulatory protein	0.7095 mf	0.5749194
Transketolase	0.7109 mf	0.5749194
Sulfur redox metabolism	0.7126 bp	0.5749194
Metalloprotease inhibitor	0.7129 mf	0.5749194
G-protein	0.7154 mf	0.5749398
Receptor mediated endocytosis	0.7158 bp	0.5749398
Voltage-gated potassium channel	0.7191 mf	0.5756693
Nicotine degradation	0.7221 pw	0.5756693
TGF-beta receptor	0.7227 mf	0.5756693
Regulation of carbohydrate metabolism	0.7231 bp	0.5756693
Guanyl-nucleotide exchange factor	0.7241 mf	0.5756693
Oxidoreductase	0.726 mf	0.5756693
Complement component	0.7283 mf	0.5756693
NO mediated signal transduction	0.7304 bp	0.5756693
Epimerase_racemase	0.7307 mf	0.5756693
Miscellaneous function	0.7311 mf	0.5756693
Nitric oxide biosynthesis	0.7329 bp	0.5759528
Basal transcription factor	0.736 mf	0.5772549
Methyltransferase	0.7437 mf	0.5810937
2-arachidonoylglycerol biosynthesis	0.7438 pw	0.5810937
DNA photolyase	0.7498 mf	0.5846394
ATP-binding cassette ABC transporter	0.7576 mf	0.5892248
Gap junction	0.7592 mf	0.5892248
Adrenaline and noradrenaline biosynthesis	0.7601 pw	0.5892248
Coenzyme metabolism	0.7653 bp	0.5896552
Exoribonuclease	0.7677 mf	0.5896552
Cytokine receptor	0.7685 mf	0.5896552
Carnitine metabolism	0.7689 pw	0.5896552
Carnitine and CoA metabolism	0.7694 pw	0.5896552
Hsp 90 family chaperone	0.7695 mf	0.5896552
Non-vertebrate process	0.7754 bp	0.591619
Blood coagulation	0.7763 pw	0.591619
N-acetylglucosamine metabolism	0.7765 pw	0.591619
Lactation, mammary development	0.7815 bp	0.5942966
Oxidase	0.7894 mf	0.5953383
Microtubule family cytoskeletal protein	0.7898 mf	0.5953383
Mitochondrial carrier protein	0.7899 mf	0.5953383
Other ion channel	0.7899 mf	0.5953383
TGF-beta signaling pathway	0.7915 pw	0.5953383
Heterotrimeric G-protein signaling pathway-Gi alpha and Gs alpha mediated pathway	0.7918 pw	0.5953383
Other chaperones	0.7948 mf	0.5957383
Other sensory perception	0.7956 bp	0.5957383
Other neuronal activity	0.7968 bp	0.5957383
Small molecule transport	0.8025 bp	0.5988806
Ionotropic glutamate receptor pathway	0.8143 pw	0.6054815
Glutamate receptor	0.8169 mf	0.6054815
Other protease inhibitor	0.8169 mf	0.6054815

Blood circulation and gas exchange	0.8174 bp	0.6054815
JAK_STAT signaling pathway	0.8236 pw	0.6084133
Carbohydrate transporter	0.8244 mf	0.6084133
Monosaccharide metabolism	0.8273 bp	0.6094291
Transcription cofactor	0.8311 mf	0.6101989
Cation transport	0.8333 bp	0.6101989
Metalloprotease	0.8362 mf	0.6101989
mRNA splicing	0.8391 pw	0.6101989
Constitutive exocytosis	0.8392 bp	0.6101989
mRNA splicing factor	0.8394 mf	0.6101989
Phosphatase	0.8398 mf	0.6101989
Protein ADP-ribosylation	0.8416 bp	0.6101989
Other steroid metabolism	0.8431 bp	0.6101989
Other G-protein modulator	0.8436 mf	0.6101989
Ubiquitin-protein ligase	0.8475 mf	0.6114387
Hydratase	0.8521 mf	0.6114387
Nuclear transport	0.8526 bp	0.6114387
Heterotrimeric G-protein signaling pathway-rod outer segment phototransduction	0.8548 pw	0.6114387
Pentose phosphate pathway	0.8551 pw	0.6114387
Voltage-gated calcium channel	0.8582 mf	0.6114387
Esterase	0.8583 mf	0.6114387
Dehydrogenase	0.8592 mf	0.6114387
Salvage pyrimidine deoxyribonucleotides	0.8593 pw	0.6114387
mRNA transcription initiation	0.8606 bp	0.6114387
Acetate utilization	0.8626 pw	0.611773
KRAB box transcription factor	0.8684 mf	0.6147965
Carbohydrate phosphatase	0.8736 mf	0.6156338
Other mRNA transcription	0.8739 bp	0.6156338
Polyphosphate biosynthesis	0.8742 bp	0.6156338
Other zinc finger transcription factor	0.8784 mf	0.6175044
Non-motor microtubule binding protein	0.8828 mf	0.6195088
RNA methyltransferase	0.8858 mf	0.6205254
Mannose metabolism	0.8874 pw	0.6205594
Other extracellular matrix	0.89 mf	0.6212914
DNA degradation	0.8918 bp	0.6214634
rRNA metabolism	0.8963 bp	0.623513
SNARE protein	0.9029 mf	0.6246632
Other kinase	0.903 mf	0.6246632
Neuropeptide	0.9033 mf	0.6246632
DNA metabolism	0.9042 bp	0.6246632
Protein methylation	0.9165 bp	0.6305155
5HT1 type receptor mediated signaling pathway	0.9166 pw	0.6305155
Purine metabolism	0.9174 pw	0.6305155
5HT3 type receptor mediated signaling pathway	0.9208 pw	0.6317667
Non-receptor tyrosine protein kinase	0.9228 mf	0.6320548
Fructose galactose metabolism	0.9246 pw	0.6322051
Leucine biosynthesis	0.9278 pw	0.6328257
Carbohydrate kinase	0.9288 mf	0.6328257
Blood clotting	0.9308 bp	0.6328257
Alanine biosynthesis	0.9335 pw	0.6328257
Sex determination	0.9349 bp	0.6328257
Folate biosynthesis	0.935 pw	0.6328257
Reverse transcription	0.9377 bp	0.6335811

RNA localization	0.9456	bp	0.6378415
General transcription regulation	0.9489	pw	0.6389899
Amino acid metabolism regulation	0.9543	bp	0.6415462
Galactosidase	0.9571	mf	0.6416054
Fatty acid desaturation	0.958	bp	0.6416054
Opioid proopiomelanocortin pathway	0.9592	pw	0.6416054
Homeobox transcription factor	0.963	mf	0.6430718
Other transcription factor	0.9669	mf	0.6431229
Opioid proenkephalin pathway	0.9672	pw	0.6431229
Metabotropic glutamate receptor group III pathway	0.9679	pw	0.6431229
Salvage pyrimidine ribonucleotides	0.973	pw	0.6454395
Metabotropic glutamate receptor group II pathway	0.9752	pw	0.6458278
Cyclase	0.9778	mf	0.6462706
Amylase	0.9791	mf	0.6462706
Glycogen metabolism	0.9839	bp	0.648369
Protein acetylation	0.9903	bp	0.6510458
Other enzyme regulator	0.9945	mf	0.6510458
Serine glycine biosynthesis	0.995	pw	0.6510458
Glucose homeostasis	0.9953	bp	0.6510458
Taste	0.9961	bp	0.6510458
Other DNA-binding protein	0.9996	mf	0.6522675

2.3 DE similarity (dot product)

col 1	col 2	col 3	col 4
Cell adhesion	0.0016	bp	0.2423071
Transport	0.002	bp	0.2423071
Wnt signaling pathway	0.0023	pw	0.2423071
Cell structure	0.0026	bp	0.2423071
Other protein targeting and localization	0.0037	bp	0.2423071
G-protein mediated signaling	0.0039	bp	0.2423071
Vitamin metabolism	0.0048	bp	0.2423071
Protease inhibitor	0.0057	mf	0.2423071
Hydroxylase	0.0059	mf	0.2423071
DNA replication	0.0074	bp	0.2423071
EGF receptor signaling pathway	0.0081	pw	0.2423071
Amino acid transporter	0.0084	mf	0.2423071
Lipid and fatty acid binding	0.0092	bp	0.2423071
FGF signaling pathway	0.0099	pw	0.2423071
G-protein coupled receptor	0.01	mf	0.2423071
Parkinson disease	0.0106	pw	0.2423071
Actin and actin related protein	0.0107	mf	0.2423071
Muscle contraction	0.0114	bp	0.2423071
Lipid and fatty acid transport	0.0115	bp	0.2423071
Amino acid biosynthesis	0.0131	bp	0.2615385
Phosphatase modulator	0.0142	mf	0.2615385
Cell adhesion-mediated signaling	0.0146	bp	0.2615385
Oogenesis	0.0162	bp	0.2615385
Tight junction	0.0165	mf	0.2615385
Vitamin_cofactor transport	0.0169	bp	0.2615385
Cytokinesis	0.017	bp	0.2615385
Steroid metabolism	0.0201	bp	0.2615385
Other lyase	0.0203	mf	0.2615385
Extracellular matrix glycoprotein	0.021	mf	0.2615385

Endocytosis	0.0214 bp	0.2615385
Steroid hormone-mediated signaling	0.0219 bp	0.2615385
Embryogenesis	0.0223 bp	0.2615385
DNA replication	0.0231 pw	0.2615385
Interferon receptor	0.0235 mf	0.2615385
Peptide hormone	0.0243 mf	0.2615385
Neurogenesis	0.0243 bp	0.2615385
Myelin protein	0.0271 mf	0.2615385
Exocytosis	0.0272 bp	0.2615385
Ectoderm development	0.0274 bp	0.2615385
Lysosome transport	0.0276 bp	0.2615385
Phenylethylamine degradation	0.0287 pw	0.2615385
Vesicle coat protein	0.0295 mf	0.2615385
DNA topoisomerase	0.03 mf	0.2615385
Proteolysis	0.0303 bp	0.2615385
Basic helix-loop-helix transcription factor	0.0309 mf	0.2615385
Cell motility	0.0315 bp	0.2615385
mRNA processing factor	0.0322 mf	0.2615385
Signal transduction	0.0328 bp	0.2615385
Tubulin	0.0331 mf	0.2615385
Zinc finger transcription factor	0.0335 mf	0.2615385
Protein glycosylation	0.0336 bp	0.2615385
Calcium mediated signaling	0.034 bp	0.2615385
Oxygenase	0.0357 mf	0.265
PI3 kinase pathway	0.0366 pw	0.265
Hedgehog signaling pathway	0.0369 pw	0.265
Amino acid transport	0.0371 bp	0.265
Electron transport	0.0391 bp	0.274386
HMG box transcription factor	0.0428 mf	0.2901695
Other carbon metabolism	0.0428 bp	0.2901695
Phagocytosis	0.0456 bp	0.2984615
Cadherin signaling pathway	0.0462 pw	0.2984615
Dehydratase	0.0463 mf	0.2984615
Other transferase	0.0476 mf	0.2984615
p53 pathway	0.0484 pw	0.2984615
Other immune and defense	0.0485 bp	0.2984615
Peroxidase	0.0507 mf	0.3068571
5-Hydroxytryptamine degredation	0.0528 pw	0.3068571
Cysteine protease inhibitor	0.053 mf	0.3068571
Guanylate cyclase	0.0531 mf	0.3068571
Other receptor	0.0537 mf	0.3068571
Extracellular matrix structural protein	0.0549 mf	0.3092958
Receptor	0.0586 mf	0.3255556
Other transfer_carrier protein	0.0595 mf	0.3260274
FAS signaling pathway	0.0617 pw	0.3293458
Extracellular matrix	0.0622 mf	0.3293458
Metabotropic glutamate receptor group I pathway	0.064 pw	0.3293458
Inflammation mediated by chemokine and cytokine signaling pathway	0.0654 pw	0.3293458
Transcription factor	0.0656 mf	0.3293458
Serine protease inhibitor	0.0659 mf	0.3293458
Aminobutyrate degradation	0.0667 pw	0.3293458
Storage protein	0.0672 mf	0.3293458
Phosphatase inhibitor	0.0696 mf	0.3293458

Huntington disease	0.0701	pw	0.3293458
mRNA splicing	0.0702	bp	0.3293458
B cell activation	0.0714	pw	0.3293458
Translation factor	0.0736	mf	0.3293458
Growth factor homeostasis	0.0741	bp	0.3293458
Cell adhesion molecule	0.0744	mf	0.3293458
T cell activation	0.0746	pw	0.3293458
General vesicle transport	0.0756	bp	0.3293458
DNA helicase	0.0763	mf	0.3293458
Neuronal activities	0.0776	bp	0.3293458
Cytokine_chemokine mediated immunity	0.0786	bp	0.3293458
Phosphate metabolism	0.0788	bp	0.3293458
Alpha adrenergic receptor signaling pathway	0.0788	pw	0.3293458
Apoptotic processes	0.0796	bp	0.3293458
Angiogenesis	0.0817	pw	0.3293458
DNA repair	0.0822	bp	0.3293458
Induction of apoptosis	0.0823	bp	0.3293458
Primase	0.0838	mf	0.3293458
Cadherin	0.0848	mf	0.3293458
Chaperonin	0.085	mf	0.3293458
Transfer_carrier protein	0.0857	mf	0.3293458
Membrane traffic protein	0.0858	mf	0.3293458
VEGF signaling pathway	0.0874	pw	0.3293458
Histamine H1 receptor mediated signaling pathway	0.088	pw	0.3293458
Chromatin packaging and remodeling	0.0881	bp	0.3293458
Carnitine and CoA metabolism	0.0898	pw	0.3300901
Fatty acid metabolism	0.09	bp	0.3300901
Methylcitrate cycle	0.091	pw	0.3300901
Carnitine metabolism	0.0916	pw	0.3300901
Actin binding motor protein	0.0945	mf	0.3375
Developmental processes	0.0957	bp	0.3387611
Hydrogen transporter	0.0972	mf	0.3389655
Damaged DNA-binding protein	0.0979	mf	0.3389655
Cholesterol metabolism	0.0983	bp	0.3389655
Other amino acid metabolism	0.1002	bp	0.342521
Actin binding cytoskeletal protein	0.1012	mf	0.342521
Other sulfur metabolism	0.1019	bp	0.342521
Cell communication	0.1031	bp	0.3436667
Other defense and immunity protein	0.107	mf	0.3511811
Acetate utilization	0.1086	pw	0.3511811
Oncogenesis	0.1091	bp	0.3511811
DNA glycosylase	0.1097	mf	0.3511811
Nitric oxide biosynthesis	0.1108	bp	0.3511811
Other homeostasis activities	0.1112	bp	0.3511811
Amino acid catabolism	0.1115	bp	0.3511811
De novo pyrmidine ribonucleotides biosythesis	0.1141	pw	0.3557447
p53 pathway feedback loops 2	0.1163	pw	0.3557447
Phospholipase	0.1184	mf	0.3557447
Regulated exocytosis	0.1193	bp	0.3557447
Select calcium binding protein	0.12	mf	0.3557447
Cell cycle	0.1201	bp	0.3557447
Pyrimidine Metabolism	0.122	pw	0.3557447
Macrophage-mediated immunity	0.1223	bp	0.3557447

Inhibition of apoptosis	0.1225	bp	0.3557447
DNA polymerase processivity factor	0.1231	mf	0.3557447
Androgen_estrogene_progesterone biosynthesis	0.1241	pw	0.3557447
Alzheimer disease-presenilin pathway	0.1243	pw	0.3557447
Proline biosynthesis	0.1248	pw	0.3557447
De novo pyrimidine deoxyribonucleotide biosynthesis	0.1254	pw	0.3557447
Calcium ion homeostasis	0.1278	bp	0.3583217
Muscarinic acetylcholine receptor 1 and 3 signaling pathway	0.1281	pw	0.3583217
Tricarboxylic acid pathway	0.1322	bp	0.3587342
Receptor protein serine_threonine kinase signaling pathway	0.1325	bp	0.3587342
Other receptor mediated signaling pathway	0.1336	bp	0.3587342
Chromosome segregation	0.1338	bp	0.3587342
Other blood circulation and gas exchange activity	0.1338	bp	0.3587342
Calmodulin related protein	0.1346	mf	0.3587342
Cell surface receptor mediated signal transduction	0.136	bp	0.3587342
Extracellular matrix protein-mediated signaling	0.1363	bp	0.3587342
Membrane-bound signaling molecule	0.1373	mf	0.3587342
Hypoxia response via HIF activation	0.1391	pw	0.3587342
Receptor protein tyrosine kinase signaling pathway	0.1395	bp	0.3587342
Receptor mediated endocytosis	0.1404	bp	0.3587342
Nerve-nerve synaptic transmission	0.1408	bp	0.3587342
Extracellular transport and import	0.1409	bp	0.3587342
GABA receptor	0.1417	mf	0.3587342
Structural protein	0.144	mf	0.3588024
Angiogenesis	0.1449	bp	0.3588024
Natural killer cell mediated immunity	0.1464	bp	0.3588024
Endothelin signaling pathway	0.1474	pw	0.3588024
Protein ADP-ribosylation	0.1476	bp	0.3588024
Angiotensin II-stimulated signaling through G proteins and beta-arrestin	0.1478	pw	0.3588024
mRNA transcription elongation	0.1487	bp	0.3588024
Transmembrane receptor regulatory_adaptor protein	0.1491	mf	0.3588024
Chemokine	0.1498	mf	0.3588024
Anion transport	0.1512	bp	0.36
Other miscellaneous function protein	0.1541	mf	0.3647337
Other actin family cytoskeletal protein	0.157	mf	0.3694118
Other membrane traffic protein	0.159	mf	0.3719298
Ion channel	0.162	mf	0.3767442
Other cell adhesion molecule	0.1632	mf	0.377341
Signaling molecule	0.1653	mf	0.3775
Circadian clock system	0.17	pw	0.3775
Endoderm development	0.1702	bp	0.3775
Tumor necrosis factor family member	0.1703	mf	0.3775
Oxytocin receptor mediated signaling pathway	0.1716	pw	0.3775
CREB transcription factor	0.1733	mf	0.3775
Other metabolism	0.1752	bp	0.3775
Apoptosis signaling pathway	0.1775	pw	0.3775
Other signaling molecule	0.1778	mf	0.3775
Transcription regulation by bZIP transcription factor	0.178	pw	0.3775
Stress response	0.1784	bp	0.3775
Neuromuscular synaptic transmission	0.1793	bp	0.3775
Thyrotropin-releasing hormone receptor signaling pathway	0.1793	pw	0.3775
Protein complex assembly	0.1799	bp	0.3775
Protein biosynthesis	0.1802	bp	0.3775

T-cell mediated immunity	0.1804	bp	0.3775
Lyase	0.1808	mf	0.3775
Microtubule binding motor protein	0.1812	mf	0.3775
Ligand-mediated signaling	0.1812	bp	0.3775
Defense_immunity protein	0.1824	mf	0.377641
DNA-directed DNA polymerase	0.1838	mf	0.377641
Pre-mRNA processing	0.1841	bp	0.377641
Transferase	0.1858	mf	0.3783838
Vision	0.187	bp	0.3783838
N-acetylglucosamine metabolism	0.1873	pw	0.3783838
De novo purine biosynthesis	0.1894	pw	0.3807035
Tumor necrosis factor receptor	0.1918	mf	0.3836
5HT2 type receptor mediated signaling pathway	0.193	pw	0.3837624
Small GTPase	0.1938	mf	0.3837624
Intracellular signaling cascade	0.1979	bp	0.3899507
Purine metabolism	0.2009	bp	0.3939216
JNK cascade	0.2019	bp	0.3939512
Glycosidase	0.2061	mf	0.3963981
Cytokine and chemokine mediated signaling pathway	0.2068	bp	0.3963981
Synthase and synthetase	0.2078	mf	0.3963981
ATP synthesis	0.2082	pw	0.3963981
Phenylalanine biosynthesis	0.2086	pw	0.3963981
Cortocotropin releasing factor receptor signaling pathway	0.2091	pw	0.3963981
Protein modification	0.2105	bp	0.3971698
Regulation of phosphate metabolism	0.2118	bp	0.3977465
Phosphate transport	0.2139	bp	0.3986977
Meiosis	0.2143	bp	0.3986977
Other nucleoside, nucleotide and nucleic acid metabolism	0.2171	bp	0.4012844
Glycosyltransferase	0.2178	mf	0.4012844
Glycolysis	0.2187	bp	0.4012844
Cation transport	0.2229	bp	0.406
Neurotrophic factor	0.2233	mf	0.406
Asymmetric protein localization	0.226	bp	0.4065517
Annexin	0.2276	mf	0.4065517
Extracellular matrix linker protein	0.2291	mf	0.4065517
Intracellular protein traffic	0.2297	bp	0.4065517
Serine protease	0.2305	mf	0.4065517
Vitamin biosynthesis	0.2321	bp	0.4065517
Other ligase	0.2327	mf	0.4065517
Exodeoxyribonuclease	0.2329	mf	0.4065517
Endoribonuclease	0.2339	mf	0.4065517
G-protein modulator	0.2347	mf	0.4065517
Protein targeting	0.2355	bp	0.4065517
Chromatin_chromatin-binding protein	0.2358	mf	0.4065517
Other cytokine	0.2403	mf	0.4125322
Ribosomal protein	0.2414	mf	0.4126496
Coenzyme A biosynthesis	0.2444	pw	0.416
Cation transporter	0.2467	mf	0.4168776
Other oncogenesis	0.247	bp	0.4168776
Single-stranded DNA-binding protein	0.2486	mf	0.4170492
Determination of dorsal_ventral axis	0.2515	bp	0.4170492
Spermatogenesis and motility	0.2517	bp	0.4170492
Other isomerase	0.2522	mf	0.4170492

Other synthase_synthetase	0.2534 mf	0.4170492
P53 pathway feedback loops 1	0.2537 pw	0.4170492
mRNA end-processing and stability	0.2544 bp	0.4170492
Other developmental process	0.2568 bp	0.4183133
Large G-protein	0.2573 mf	0.4183133
Carbohydrate transport	0.2586 bp	0.4183133
Tyrosine biosynthesis	0.2602 pw	0.4183133
Cell junction protein	0.2604 mf	0.4183133
Hydrolase	0.2623 mf	0.4193701
Antibacterial response protein	0.2655 mf	0.4193701
Other apoptosis	0.266 bp	0.4193701
Immunoglobulin receptor family member	0.2662 mf	0.4193701
Cysteine protease	0.2663 mf	0.4193701
Skeletal development	0.2687 bp	0.4214902
Kinase activator	0.273 mf	0.4249612
mRNA splicing	0.2735 pw	0.4249612
Alzheimer disease-amyloid secretase pathway	0.2741 pw	0.4249612
Plasminogen activating cascade	0.2781 pw	0.4253992
Methyltransferase	0.2788 mf	0.4253992
Other carbohydrate metabolism	0.279 bp	0.4253992
Pyrimidine metabolism	0.2793 bp	0.4253992
Lysine biosynthesis	0.2797 pw	0.4253992
Carbohydrate metabolism	0.2819 bp	0.4271212
Integrin signalling pathway	0.285 pw	0.4294424
Protein-lipid modification	0.2864 bp	0.4294424
Other phosphatase	0.2877 mf	0.4294424
B-cell- and antibody-mediated immunity	0.288 bp	0.4294424
Cholesterol biosynthesis	0.2888 pw	0.4294424
DNA recombination	0.2914 bp	0.4317037
Oncogene	0.2977 bp	0.4359124
Nucleic acid binding	0.2979 mf	0.4359124
Pyruvate metabolism	0.2979 pw	0.4359124
Other cell junction protein	0.2986 mf	0.4359124
Heterotrimeric G-protein signaling pathway-Gq alpha and Go alpha mediated pathway	0.3009 pw	0.4376727
Regulation of carbohydrate metabolism	0.303 bp	0.4377061
Other intracellular protein traffic	0.3044 bp	0.4377061
Ribonucleoprotein	0.3052 mf	0.4377061
Ion transport	0.3053 bp	0.4377061
Other select calcium binding proteins	0.3067 mf	0.4381429
NO mediated signal transduction	0.3092 bp	0.4389189
Immunity and defense	0.3113 bp	0.4389189
DNA degradation	0.3126 bp	0.4389189
Fertilization	0.3154 bp	0.4389189
Non-motor actin binding protein	0.3157 mf	0.4389189
Coenzyme and prosthetic group metabolism	0.3157 bp	0.4389189
Sulfur metabolism	0.3175 bp	0.4389189
Allantoin degradation	0.3181 pw	0.4389189
Growth factor	0.3189 mf	0.4389189
Nucleotidyltransferase	0.3191 mf	0.4389189
Aspartic protease	0.3196 mf	0.4389189
Synthase	0.322 mf	0.4389189
Steroid hormone metabolism	0.3226 bp	0.4389189
Fatty acid beta-oxidation	0.323 bp	0.4389189

Regulation of vasoconstriction, dilation	0.3246 bp	0.4389189
Mutase	0.3248 mf	0.4389189
Other steroid metabolism	0.3302 bp	0.4423607
Other pathways of electron transport	0.3306 bp	0.4423607
Tyrosine protein kinase receptor	0.3313 mf	0.4423607
Cytoskeletal regulation by Rho GTPase	0.3349 pw	0.4423607
Protease	0.3355 mf	0.4423607
mRNA transcription termination	0.3356 bp	0.4423607
Serine_threonine protein kinase receptor	0.3361 mf	0.4423607
Axon guidance mediated by semaphorins	0.3362 pw	0.4423607
Histamine H2 receptor mediated signaling pathway	0.3373 pw	0.4423607
Adenine and hypoxanthine salvage pathway	0.3397 pw	0.4440523
Interleukin	0.3435 mf	0.4458786
MAPKKK cascade	0.3447 bp	0.4458786
Oxidative stress response	0.3451 pw	0.4458786
Miscellaneous	0.3465 bp	0.4458786
p38 MAPK pathway	0.3476 pw	0.4458786
Other oxidoreductase	0.3489 mf	0.4458786
Amino acid metabolism	0.3489 bp	0.4458786
Protein phosphatase	0.3508 mf	0.446879
DNA-directed RNA polymerase	0.3522 mf	0.4472381
Apoptosis	0.3542 bp	0.4483544
Protein targeting and localization	0.3578 bp	0.4509434
PDGF signaling pathway	0.3585 pw	0.4509434
Interferon	0.3631 mf	0.4519149
Insulin_IGF pathway-mitogen activated protein kinase kinase_MAP kinase cascade	0.3656 pw	0.4519149
DNA methyltransferase	0.3657 mf	0.4519149
Membrane traffic regulatory protein	0.3664 mf	0.4519149
CAM family adhesion molecule	0.3676 mf	0.4519149
Acetylcholine receptor	0.3687 mf	0.4519149
Translation elongation factor	0.3689 mf	0.4519149
Immunoglobulin	0.3691 mf	0.4519149
Protein disulfide-isomerase reaction	0.3704 bp	0.4519149
Synaptic_vesicle_trafficking	0.3712 pw	0.4519149
Translational regulation	0.3717 bp	0.4519149
mRNA polyadenylation	0.3736 bp	0.4528485
Apolipoprotein	0.3755 mf	0.4537764
JAK-STAT cascade	0.3792 bp	0.4551642
Gametogenesis	0.3802 bp	0.4551642
Voltage-gated ion channel	0.3812 mf	0.4551642
Protein metabolism and modification	0.3812 bp	0.4551642
Vasopressin synthesis	0.3891 pw	0.4632143
Phosphodiesterase	0.3925 mf	0.4654335
Gluconeogenesis	0.3957 bp	0.4654335
Synthetase	0.397 mf	0.4654335
Constitutive exocytosis	0.3975 bp	0.4654335
mRNA transcription	0.3991 bp	0.4654335
Nucleotide phosphatase	0.3994 mf	0.4654335
Transporter	0.4003 mf	0.4654335
Galactosidase	0.4008 mf	0.4654335
Endodeoxyribonuclease	0.4023 mf	0.4654335
Olfaction	0.4026 bp	0.4654335
Protein kinase	0.4068 mf	0.4661111

Muscle development	0.4086 bp	0.4661111
TCA cycle	0.4097 pw	0.4661111
Adenylate cyclase	0.4101 mf	0.4661111
Antioxidation and free radical removal	0.4107 bp	0.4661111
Toll receptor signaling pathway	0.4112 pw	0.4661111
Interferon-gamma signaling pathway	0.4128 pw	0.4661111
Non-vertebrate process	0.4151 bp	0.4661111
Granulocyte-mediated immunity	0.4156 bp	0.4661111
Other hydrolase	0.4162 mf	0.4661111
mRNA polyadenylation factor	0.4184 mf	0.4661111
Cell proliferation and differentiation	0.4194 bp	0.4661111
Xanthine and guanine salvage pathway	0.4194 pw	0.4661111
Hearing	0.4195 bp	0.4661111
Nicotinic acetylcholine receptor signaling pathway	0.4212 pw	0.4667036
Hydratase	0.4228 mf	0.4671823
Notch signaling pathway	0.4241 pw	0.4673278
Protein folding	0.4261 bp	0.4682418
Other enzyme activator	0.4279 mf	0.4689315
Valine biosynthesis	0.4322 pw	0.4723497
Isoleucine biosynthesis	0.4337 pw	0.4726975
General transcription by RNA polymerase I	0.4386 pw	0.4747781
Acyl-CoA metabolism	0.4399 bp	0.4747781
Pentose phosphate pathway	0.4399 pw	0.4747781
MHCI-mediated immunity	0.4411 bp	0.4747781
Other transporter	0.4438 mf	0.4747781
JAK_STAT signaling pathway	0.4441 pw	0.4747781
Centromere DNA-binding protein	0.4454 mf	0.4747781
Aldolase	0.4461 mf	0.4747781
Kinase modulator	0.4464 mf	0.4747781
Gamma-aminobutyric acid synthesis	0.4477 pw	0.4747781
Deacetylase	0.4505 mf	0.4747781
Aminoacyl-tRNA synthetase	0.4512 mf	0.4747781
Lipase	0.4518 mf	0.4747781
Esterase	0.4523 mf	0.4747781
Other cytoskeletal proteins	0.4536 mf	0.4747781
Lipoate_biosynthesis	0.4546 pw	0.4747781
Protein phosphorylation	0.4619 bp	0.481134
Carbohydrate kinase	0.4646 mf	0.481134
Other coenzyme and prosthetic group metabolism	0.4649 bp	0.481134
Other protein metabolism	0.4659 bp	0.481134
Nicotine degradation	0.4667 pw	0.481134
Homeostasis	0.4689 bp	0.481596
Coenzyme metabolism	0.4723 bp	0.481596
Sensory perception	0.4724 bp	0.481596
Interleukin receptor	0.4748 mf	0.481596
Monosaccharide metabolism	0.4748 bp	0.481596
Ascorbate degradation	0.4759 pw	0.481596
Asparagine and aspartate biosynthesis	0.4767 pw	0.481596
Other neuronal activity	0.4783 bp	0.481596
Reverse transcriptase	0.4801 mf	0.481596
Mannose metabolism	0.4804 pw	0.481596
Oxidoreductase	0.4816 mf	0.481596
Helicase	0.4821 mf	0.481596

NF-kappaB cascade	0.4828	bp	0.481596
Thiamine metabolism	0.4866	pw	0.482233
5-Hydroxytryptamine biosynthesis	0.4871	pw	0.482233
Phosphorylase	0.4931	mf	0.482233
Pterin metabolism	0.4932	bp	0.482233
Metabolism of cyclic nucleotides	0.494	bp	0.482233
Interleukin signaling pathway	0.494	pw	0.482233
Cell cycle	0.4954	pw	0.482233
Transaminase	0.4956	mf	0.482233
Isomerase	0.4958	mf	0.482233
Endogenous_cannabinoid_signaling	0.4958	pw	0.482233
Ras Pathway	0.4967	pw	0.482233
Other intracellular signaling cascade	0.4983	bp	0.482615
Peroxisome transport	0.5027	bp	0.4842206
Salvage pyrimidine deoxyribonucleotides	0.5037	pw	0.4842206
Other microtubule family cytoskeletal protein	0.5044	mf	0.4842206
Other zinc finger transcription factor	0.5048	mf	0.4842206
Fatty acid biosynthesis	0.5078	bp	0.485933
Neurotransmitter release	0.5116	bp	0.488401
Select regulatory molecule	0.5146	mf	0.4900952
Nucleoside, nucleotide and nucleic acid transport	0.5242	bp	0.4975059
Intermediate filament binding protein	0.5253	mf	0.4975059
Cytoskeletal protein	0.5274	mf	0.4975059
Heterotrimeric G-protein signaling pathway-rod outer segment phototransduction	0.5274	pw	0.4975059
Surfactant	0.5286	mf	0.4975059
Decarboxylase	0.5319	mf	0.4994366
Nuclear transport	0.5357	bp	0.5018267
Pyrophosphatase	0.5397	mf	0.5043925
Other ligand-gated ion channel	0.5421	mf	0.5054545
Intermediate filament	0.5454	mf	0.5070998
Gut mesoderm development	0.5464	bp	0.5070998
Methylmalonyl pathway	0.5548	pw	0.5134562
Anion channel	0.5561	mf	0.5134562
Kinase	0.5571	mf	0.5134562
Hsp 90 family chaperone	0.5596	mf	0.5140367
Chemosensory perception	0.5603	bp	0.5140367
Disaccharide metabolism	0.5616	bp	0.5140503
Pentose-phosphate shunt	0.5643	bp	0.5153425
Pyridoxal phosphate salvage pathway	0.5671	pw	0.5167198
Cytokine	0.5697	mf	0.5173696
Cell cycle control	0.5704	bp	0.5173696
Voltage-gated sodium channel	0.5764	mf	0.521629
Nuclear hormone receptor	0.5779	mf	0.5218059
Double-stranded DNA binding protein	0.5819	mf	0.5242342
Oxidative phosphorylation	0.5834	bp	0.5243946
Epimerase_racemase	0.5847	mf	0.5243946
DNA ligase	0.5869	mf	0.5244444
Acyltransferase	0.5893	mf	0.5244444
tRNA metabolism	0.5898	bp	0.5244444
Transketolase	0.59	mf	0.5244444
Other enzyme inhibitor	0.5916	mf	0.5247007
Pain sensation	0.6062	bp	0.5364602
Guanyl-nucleotide exchange factor	0.6087	mf	0.5374834

Interferon-mediated immunity	0.6132 bp	0.538421
MHCII-mediated immunity	0.6136 bp	0.538421
Heart development	0.6138 bp	0.538421
Hematopoiesis	0.6186 bp	0.540524
Formyltetrahydroformate biosynthesis	0.6189 pw	0.540524
Other sensory perception	0.6219 bp	0.5419608
Blood clotting	0.6256 bp	0.54329
GABA-B_receptor_II_signaling	0.6269 pw	0.54329
Enkephalin release	0.6275 pw	0.54329
SNARE protein	0.6289 mf	0.5433261
Lipid metabolism	0.6363 bp	0.5484979
Heterotrimeric G-protein signaling pathway-Gi alpha and Gs alpha mediated pathway	0.6384 pw	0.5484979
Ligase	0.639 mf	0.5484979
RNA helicase	0.6411 mf	0.5491221
Neuropeptide	0.6437 mf	0.5501709
Axon guidance mediated by netrin	0.647 pw	0.5518124
Blood coagulation	0.651 pw	0.5532203
Transcription cofactor	0.6528 mf	0.5532203
Mitosis	0.6528 bp	0.5532203
Major histocompatibility complex antigen	0.6551 mf	0.5539958
Cyclic nucleotide-gated ion channel	0.658 mf	0.5552743
Non-motor microtubule binding protein	0.6617 mf	0.5568067
Metalloprotease inhibitor	0.6626 mf	0.5568067
Translation release factor	0.6673 mf	0.5578333
Vitamin B6 metabolism	0.6684 pw	0.5578333
Other cell cycle process	0.6692 bp	0.5578333
Axon guidance mediated by Slit_Robo	0.6694 pw	0.5578333
Ubiquitin proteasome pathway	0.6758 pw	0.560911
Hsp 70 family chaperone	0.6767 mf	0.560911
Protein methylation	0.6773 bp	0.560911
Lactation, mammary development	0.6853 bp	0.5655258
Other proteases	0.6857 mf	0.5655258
Reverse transcription	0.6901 bp	0.5674743
Detoxification	0.6909 bp	0.5674743
Dehydrogenase	0.6942 mf	0.5678528
Muscarinic acetylcholine receptor 2 and 4 signaling pathway	0.6942 pw	0.5678528
Arginine biosynthesis	0.6981 pw	0.5694165
Gap junction	0.7007 mf	0.5694165
Blood circulation and gas exchange	0.702 bp	0.5694165
Other RNA-binding protein	0.704 mf	0.5694165
Phospholipid metabolism	0.7047 bp	0.5694165
p53 pathway by glucose deprivation	0.7067 pw	0.5694165
Synaptic transmission	0.7072 bp	0.5694165
Other cytokine receptor	0.7075 mf	0.5694165
2-arachidonoylglycerol biosynthesis	0.709 pw	0.5694779
Purine metabolism	0.7118 pw	0.56968
Deaminase	0.7121 mf	0.56968
G-protein	0.72 mf	0.5748503
Nuclease	0.7229 mf	0.5757002
Miscellaneous function	0.7251 mf	0.5757002
Mitochondrial transport	0.7264 bp	0.5757002
Histone	0.7273 mf	0.5757002
Cytokine receptor	0.7286 mf	0.5757002

Acetyltransferase	0.7297	mf	0.5757002
Other nucleic acid binding	0.7334	mf	0.5774803
Ferredoxin metabolism	0.7366	bp	0.5776998
Glutamate receptor	0.7374	mf	0.5776998
Anterior_posterior patterning	0.7387	bp	0.5776998
Action potential propagation	0.7402	bp	0.5776998
Microtubule family cytoskeletal protein	0.7409	mf	0.5776998
Succinate to proprionate conversion	0.7457	pw	0.5790366
Carbohydrate transporter	0.7458	mf	0.5790366
Complement-mediated immunity	0.7481	bp	0.5790366
ATP synthase	0.749	mf	0.5790366
Insulin_IGF pathway-protein kinase B signaling cascade	0.7501	pw	0.5790366
Glucosidase	0.7513	mf	0.5790366
Cell structure and motility	0.756	bp	0.5815385
Complement component	0.7602	mf	0.5836468
Other protease inhibitor	0.7659	mf	0.5858779
Heme biosynthesis	0.7665	pw	0.5858779
Pheromone response	0.7675	bp	0.5858779
mRNA splicing factor	0.7729	mf	0.5879087
Chaperone	0.7731	mf	0.5879087
Small molecule transport	0.7751	bp	0.5883112
Other polysaccharide metabolism	0.779	bp	0.5901515
Voltage-gated potassium channel	0.7806	mf	0.5902457
Other mRNA transcription	0.7825	bp	0.590566
Translation initiation factor	0.785	mf	0.5913371
Folate biosynthesis	0.7914	pw	0.5942222
Carbohydrate phosphatase	0.7937	mf	0.5942222
Metalloprotease	0.7959	mf	0.5942222
mRNA transcription initiation	0.7964	bp	0.5942222
Other extracellular matrix	0.7968	mf	0.5942222
mRNA capping	0.7986	bp	0.5942222
Mesoderm development	0.8007	bp	0.5942222
Glutamine glutamate conversion	0.801	pw	0.5942222
Other G-protein modulator	0.8022	mf	0.5942222
Ionotropic glutamate receptor pathway	0.8056	pw	0.5956377
Pinocytosis	0.8092	bp	0.5971956
Dopamine receptor mediated signaling pathway	0.8124	pw	0.598453
Adrenaline and noradrenaline biosynthesis	0.8212	pw	0.6032117
Sulfur redox metabolism	0.8232	bp	0.6032117
RNA catabolism	0.8234	bp	0.6032117
Segment specification	0.8256	bp	0.6032117
5HT1 type receptor mediated signaling pathway	0.8264	pw	0.6032117
Vitamin D metabolism and pathway	0.829	pw	0.6040073
ATP-binding cassette ABC transporter	0.8341	mf	0.6066182
O-antigen biosynthesis	0.8401	pw	0.608913
Opioid prodynorphin pathway	0.8403	pw	0.608913
DNA photolyase	0.8517	mf	0.6160579
Other transport	0.855	bp	0.6171532
Reductase	0.8563	mf	0.6171532
Polyphosphate biosynthesis	0.8606	bp	0.6179211
Other chaperones	0.8618	mf	0.6179211
Ubiquitin-protein ligase	0.862	mf	0.6179211
TGF-beta receptor	0.8636	mf	0.6179606

Replication origin binding protein	0.8697	mf	0.6211765
Glycolysis	0.8712	pw	0.6211765
5HT4 type receptor mediated signaling pathway	0.8752	pw	0.6216312
KRAB box transcription factor	0.8754	mf	0.6216312
Voltage-gated calcium channel	0.8765	mf	0.6216312
Exoribonuclease	0.8787	mf	0.6220885
Other signal transduction	0.8826	bp	0.6229982
Amino acid metabolism regulation	0.8831	bp	0.6229982
Amino acid activation	0.8889	bp	0.6252921
Nucleotide kinase	0.8899	mf	0.6252921
Beta3 adrenergic receptor signaling pathway	0.8931	pw	0.6252921
Beta1 adrenergic receptor signaling pathway	0.8934	pw	0.6252921
Other ion channel	0.895	mf	0.6252921
Phosphatase	0.8999	mf	0.6252921
Fructose galactose metabolism	0.9004	pw	0.6252921
Porphyrin metabolism	0.9019	bp	0.6252921
Salvage pyrimidine ribonucleotides	0.9057	pw	0.6252921
Alanine biosynthesis	0.9064	pw	0.6252921
Leucine biosynthesis	0.9076	pw	0.6252921
Basal transcription factor	0.908	mf	0.6252921
Nitrogen metabolism	0.909	bp	0.6252921
Mitochondrial carrier protein	0.9092	mf	0.6252921
Beta2 adrenergic receptor signaling pathway	0.9098	pw	0.6252921
Tumor suppressor	0.9114	bp	0.6253173
Oxidase	0.913	mf	0.6253425
Other transcription factor	0.9148	mf	0.6255043
Other kinase	0.9179	mf	0.6265529
DNA metabolism	0.9222	bp	0.6280952
Homeobox transcription factor	0.9233	mf	0.6280952
Non-receptor tyrosine protein kinase	0.925	mf	0.6281834
General transcription regulation	0.9318	pw	0.6317288
RNA methyltransferase	0.9379	mf	0.6347885
Fatty acid desaturation	0.9406	bp	0.6355405
General mRNA transcription activities	0.943	bp	0.6360877
Non-receptor serine_threonine protein kinase	0.9485	mf	0.6387205
RNA localization	0.9501	bp	0.6387227
5HT3 type receptor mediated signaling pathway	0.9521	pw	0.6389933
Sex determination	0.9541	bp	0.639263
rRNA metabolism	0.9604	bp	0.642408
Cyclase	0.9667	mf	0.6455426
Metabotropic glutamate receptor group III pathway	0.9792	pw	0.6500984
Glycogen metabolism	0.9809	bp	0.6500984
Opioid proopiomelanocortin pathway	0.9823	pw	0.6500984
Glucose homeostasis	0.9824	bp	0.6500984
Metabotropic glutamate receptor group II pathway	0.9836	pw	0.6500984
Amylase	0.9845	mf	0.6500984
Opioid proenkephalin pathway	0.987	pw	0.6500984
Kinase inhibitor	0.9877	mf	0.6500984
Other enzyme regulator	0.9896	mf	0.6500984
Serine glycine biosynthesis	0.9911	pw	0.6500984
Protein acetylation	0.9914	bp	0.6500984
Taste	0.9948	bp	0.6508497
TGF-beta signaling pathway	0.9958	pw	0.6508497

Other DNA-binding protein	0.9975 mf	0.6508972

3. Broad functional categories of E2F target-cohorts
LEGEND:
col1- gene group
col 2- gene group type
col 3- metric(s)
col 4- broad category

col 1	col 2	col 3	col 4
Calcium mediated signaling	bp	de,dp,cov	Signaling related
G-protein mediated signaling	bp	de,dp,cov	Signaling related
Steroid hormone-mediated signaling	bp	de,dp,cov	Signaling related
Cell adhesion-mediated signaling	bp	de,dp	Signaling related
JNK cascade	bp	de,cov	Signaling related
MAPKKK cascade	bp	de,cov	Signaling related
Receptor protein tyrosine kinase signaling pathway	bp	de	Signaling related
Cell communication	bp	cov	Signaling related
Extracellular matrix protein-mediated signaling	bp	cov	Signaling related
Intracellular signaling cascade	bp	cov	Signaling related
Signal transduction	bp	dp	Signaling related
EGF receptor signaling pathway	pw	de,dp,cov	Signaling related
FAS signaling pathway	pw	de,dp,cov	Signaling related
FGF signaling pathway	pw	de,dp,cov	Signaling related
Hedgehog signaling pathway	pw	de,dp,cov	Signaling related
Wnt signaling pathway	pw	de,dp,cov	Signaling related
PI3 kinase pathway	pw	dp,cov	Signaling related
Oxytocin receptor mediated signaling pathway	pw	de,cov	Signaling related
p38 MAPK pathway	pw	de,cov	Signaling related
Insulin_IGF pathway-mitogen activated protein kinase kinase_	pw	cov	Signaling related
Alpha adrenergic receptor signaling pathway	pw	de,dp	Signaling related
Cadherin signaling pathway	pw	de,dp	Signaling related
Histamine H1 receptor mediated signaling pathway	pw	de,dp	Signaling related
Metabotropic glutamate receptor group I pathway	pw	de,dp	Signaling related
VEGF signaling pathway	pw	de,dp	Signaling related
G-protein coupled receptor	mf	de,dp,cov	Signaling related
Other carbon metabolism	bp	de,dp,cov	Energy and other metabolism
Regulation of phosphate metabolism	bp	de	Energy and other metabolism
Oxidative phosphorylation	bp	cov	Energy and other metabolism
Phosphate metabolism	bp	de,dp	Energy and other metabolism
Tricarboxylic acid pathway	bp	de,cov	Energy and other metabolism
Vitamin metabolism	bp	de,dp,cov	Energy and other metabolism
Carnitine and CoA metabolism	pw	de,dp	Energy and other metabolism
Carnitine metabolism	pw	de,dp	Energy and other metabolism
Methylcitrate cycle	pw	de,dp	Energy and other metabolism
Ascorbate degradation	pw	de,cov	Energy and other metabolism
Pyruvate metabolism	pw	de,cov	Energy and other metabolism
5-Hydroxytryptamine degredation	pw	dp,cov	Energy and other metabolism
Phenylethylamine degradation	pw	dp,cov	Energy and other metabolism
ATP synthesis	pw	de	Energy and other metabolism
Acetate utilization	pw	de	Energy and other metabolism
Aminobutyrate degradation	pw	de,dp,cov	Energy and other metabolism
Electron transport	bp	de,dp,cov	Energy and other metabolism
Dehydratase	mf	de,dp,cov	Energy and other metabolism
Hydroxylase	mf	de,dp,cov	Energy and other metabolism
Oxygenase	mf	de,dp,cov	Energy and other metabolism
Peroxidase	mf	de,dp,cov	Energy and other metabolism
Other transferase	mf	de,dp,cov	Energy and other metabolism

Guanylate cyclase	*mf*	de,dp,cov	Energy and other metabolism
Other lyase	*mf*	de,dp	Energy and other metabolism
Hydrogen transporter	mf	dp,cov	Energy and other metabolism
Phospholipase	mf	de	Energy and other metabolism
Cell cycle	bp	cov	Cell cycle related
Other cell cycle process	bp	cov	Cell cycle related
Cytokinesis	bp	de,dp	Cell cycle related
DNA replication	bp	dp,cov	Cell cycle related
DNA repair	bp	dp	Cell cycle related
Cell structure	bp	de,dp,cov	Cell cycle related
De novo pyrimidine deoxyribonucleotide biosynthesis	pw	de	Cell cycle related
DNA replication	pw	de,dp,cov	Cell cycle related
De novo pyrmidine ribonucleotides biosythesis	pw	de,cov	Cell cycle related
Damaged DNA-binding protein	mf	dp,cov	Cell cycle related
DNA glycosylase	mf	cov	Cell cycle related
DNA helicase	mf	de,dp,cov	Cell cycle related
DNA topoisomerase	mf	de,dp,cov	Cell cycle related
Actin binding cytoskeletal protein	mf	de	Cell cycle related
Actin binding motor protein	mf	de,dp	Cell cycle related
Actin and actin related protein	mf	de,dp,cov	Cell cycle related
Tubulin	mf	de,dp,cov	Cell cycle related
Primase	mf	de,dp,cov	Cell cycle related
Protein glycosylation	bp	dp,cov	Others
mRNA splicing	bp	dp,cov	Others
Muscle contraction	bp	de,dp,cov	Others
Neuronal activities	bp	de,dp,cov	Others
Other protein targeting and localization	bp	de,dp,cov	Others
Growth factor homeostasis	bp	de,dp	Others
Proteolysis	bp	de,dp	Others
Vision	bp	de	Others
Hearing	bp	cov	Others
Other homeostasis activities	bp	cov	Others
Protein modification	bp	cov	Others
Parkinson disease	pw	dp,cov	Others
Oxidative stress response	pw	de,cov	Others
Hypoxia response via HIF activation	pw	de	Others
Axon guidance mediated by Slit_Robo	pw	cov	Others
Axon guidance mediated by netrin	pw	cov	Others
Other immune and defense	bp	de,dp	Others
Macrophage-mediated immunity	bp	cov	Others
Cytokine_chemokine mediated immunity	bp	dp,cov	Others
Phagocytosis	bp	dp,cov	Others
B cell activation	pw	de,dp,cov	Others
Inflammation mediated by chemokine and cytokine signaling pa	pw	de,dp,cov	Others
T cell activation	pw	de,dp,cov	Others
Basic helix-loop-helix transcription factor	mf	de,dp,cov	Others
Chaperonin	mf	de,dp,cov	Others
Cysteine protease inhibitor	mf	de,dp,cov	Others
Interferon receptor	mf	de,dp,cov	Others
Myelin protein	mf	de,dp,cov	Others
Other receptor	mf	de,dp,cov	Others
Peptide hormone	mf	de,dp,cov	Others
Protease inhibitor	mf	de,dp,cov	Others
Receptor	mf	de,dp,cov	Others
Storage protein	mf	de,dp,cov	Others
Tight junction	mf	de,dp,cov	Others
Transcription factor	mf	de,dp,cov	Others

Vesicle coat protein	mf	de,dp,cov	Others
Zinc finger transcription factor	mf	de,dp,cov	Others
mRNA processing factor	mf	de,dp,cov	Others
Translation factor	mf	de,dp,cov	Others
Calmodulin related protein	mf	cov	Others
Other miscellaneous function protein	mf	de	Others
Small GTPase	mf	de	Others
Serine protease inhibitor	mf	de,dp,cov	Others
Phosphatase inhibitor	mf	de,dp,cov	Others
Phosphatase modulator	mf	de,dp,cov	Others
Protein phosphatase	mf	cov	Others
Lipid and fatty acid transport	bp	de,dp,cov	Transport related
Lysosome transport	bp	de,dp,cov	Transport related
Transport	bp	de,dp,cov	Transport related
Vitamin_cofactor transport	bp	de,dp,cov	Transport related
Amino acid transport	bp	de,dp	Transport related
Carbohydrate transport	bp	cov	Transport related
Nucleoside, nucleotide and nucleic acid transport	bp	cov	Transport related
General vesicle transport	bp	de,dp	Transport related
Endocytosis	bp	dp,cov	Transport related
Exocytosis	bp	de,dp	Transport related
Regulated exocytosis	bp	cov	Transport related
Other membrane traffic protein	mf	cov	Transport related
Membrane traffic protein	mf	de,dp	Transport related
Transfer_carrier protein	mf	de,dp,cov	Transport related
Other transfer_carrier protein	mf	de,dp,cov	Transport related
Amino acid transporter	mf	de,dp,cov	Transport related
Other amino acid metabolism	bp	cov	Protein/lipid/carbohydrate metabolism
Other carbohydrate metabolism	bp	cov	Protein/lipid/carbohydrate metabolism
Steroid metabolism	bp	de,dp,cov	Protein/lipid/carbohydrate metabolism
Amino acid biosynthesis	bp	de,dp	Protein/lipid/carbohydrate metabolism
Fatty acid metabolism	bp	de,dp	Protein/lipid/carbohydrate metabolism
Cholesterol metabolism	bp	dp,cov	Protein/lipid/carbohydrate metabolism
Lipid and fatty acid binding	bp	de,dp,cov	Protein/lipid/carbohydrate metabolism
Cholesterol biosynthesis	pw	cov	Protein/lipid/carbohydrate metabolism
Cadherin	mf	de,dp	Cell adhesion
Extracellular matrix structural protein	mf	de,dp	Cell adhesion
Extracellular matrix	mf	de,dp,cov	Cell adhesion
Extracellular matrix glycoprotein	mf	de,dp,cov	Cell adhesion
Cell adhesion molecule	mf	de,dp,cov	Cell adhesion
Cell adhesion	bp	de,dp,cov	Cell adhesion
Ectoderm development	bp	de,dp,cov	Development related
Embryogenesis	bp	de,dp,cov	Development related
Neurogenesis	bp	de,dp,cov	Development related
Muscle development	bp	cov	Development related
Developmental processes	bp	dp	Development related
Oogenesis	bp	de,dp,cov	Development related
Chromatin packaging and remodeling	bp	de,dp	Development related
HMG box transcription factor	mf	de,dp	Development related
Angiogenesis	bp	de	Oncogene related
Apoptotic processes	bp	de,dp	Oncogene related
Induction of apoptosis	bp	de,dp	Oncogene related
Oncogene	bp	cov	Oncogene related
Cell motility	bp	de,dp	Oncogene related
p53 pathway	pw	dp,cov	Oncogene related
Angiogenesis	pw	de,dp	Oncogene related
p53 pathway feedback loops 2	pw	cov	Oncogene related
